# Genome-scale metabolic modeling of responses to polymyxins in *Pseudomonas aeruginosa*

**DOI:** 10.1093/gigascience/giy021

**Published:** 2018-03-13

**Authors:** Yan Zhu, Tobias Czauderna, Jinxin Zhao, Matthias Klapperstueck, Mohd Hafidz Mahamad Maifiah, Mei-Ling Han, Jing Lu, Björn Sommer, Tony Velkov, Trevor Lithgow, Jiangning Song, Falk Schreiber, Jian Li

**Affiliations:** 1 Monash Biomedicine Discovery Institute, Department of Microbiology, Faculty of Medicine, Nursing and Health Sciences, Monash University, Melbourne 3800, Australia; 2 Faculty of Information Technology, Monash University, Melbourne 3800, Australia; 3 Faculty of Pharmacy and Pharmaceutical Sciences, Monash University, Melbourne 3052, Australia; 4 Monash Institute of Cognitive and Clinical Neurosciences, Department of Anatomy and development biology, Faculty of Medicine, Nursing and Health Sciences, Monash University, Melbourne 3800, Australia; 5 Department of Computer and Information Science, University of Konstanz, Konstanz 78457, Germany; 6 Department of Pharmacology and Therapeutics, University of Melbourne, Melbourne 3010, Australia

**Keywords:** genome-scale metabolic model, *Pseudomonas aeruginosa*, polymyxin, lipid A modification, outer membrane

## Abstract

**Background:**

*Pseudomonas aeruginosa* often causes multidrug-resistant infections in immunocompromised patients, and polymyxins are often used as the last-line therapy. Alarmingly, resistance to polymyxins has been increasingly reported worldwide recently. To rescue this last-resort class of antibiotics, it is necessary to systematically understand how *P. aeruginosa* alters its metabolism in response to polymyxin treatment, thereby facilitating the development of effective therapies. To this end, a genome-scale metabolic model (GSMM) was used to analyze bacterial metabolic changes at the systems level.

**Findings:**

A high-quality GSMM *i*PAO1 was constructed for *P. aeruginosa* PAO1 for antimicrobial pharmacological research. Model *i*PAO1 encompasses an additional periplasmic compartment and contains 3022 metabolites, 4265 reactions, and 1458 genes in total. Growth prediction on 190 carbon and 95 nitrogen sources achieved an accuracy of 89.1%, outperforming all reported *P. aeruginosa* models. Notably, prediction of the essential genes for growth achieved a high accuracy of 87.9%. Metabolic simulation showed that lipid A modifications associated with polymyxin resistance exert a limited impact on bacterial growth and metabolism but remarkably change the physiochemical properties of the outer membrane. Modeling with transcriptomics constraints revealed a broad range of metabolic responses to polymyxin treatment, including reduced biomass synthesis, upregulated amino acid catabolism, induced flux through the tricarboxylic acid cycle, and increased redox turnover.

**Conclusions:**

Overall, *i*PAO1 represents the most comprehensive GSMM constructed to date for *Pseudomonas*. It provides a powerful systems pharmacology platform for the elucidation of complex killing mechanisms of antibiotics.

## Introduction

*Pseudomonas aeruginosa* is a common multidrug-resistant (MDR) pathogen in immune-compromised patients, cystic fibrosis patients, and burns victims [1–6]. It possesses a large genome (5.5–7.0 Mb), complex regulatory networks, remarkable metabolic versatility, and an extraordinary ability to survive extremely harsh conditions such as prolonged antibiotic exposure [7, 8]. Polymyxins (i.e., polymyxin B and colistin) have been increasingly used as a last-line therapy to treat infections caused by MDR *P. aeruginosa* [9]. Alarmingly, the prevalence of polymyxin resistance in *P. aeruginosa* has increased worldwide over the past few years [3, 10, 11].

The exact mode of action of polymyxins is not clear except for the initial electrostatic and hydrophobic interactions with lipid A, a component of the lipopolysaccharide (LPS) in the bacterial outer membrane (OM). Subsequently, the cell envelope is disorganized, cellular contents leak, oxidative stress increases, and finally cell death occurs [2, 9, 12, 13]. After polymyxin treatment, *P. aeruginosa* modifies its lipid A structure to attenuate the aforementioned electrostatic interactions [14]. Our recent metabolomics data demonstrated that, apart from lipid A modifications, numerous biochemical pathways are perturbed by polymyxin treatment, indicating that the development of polymyxin resistance by *P. aeruginosa* involves a complicated interplay of multiple cellular processes [15]. There are significant gaps in the knowledge base of the mechanisms of polymyxin activity and bacterial responses in *P. aeruginosa*, thereby necessitating comprehensive investigations using systems pharmacology approaches.

With the rapid development of genome-scale metabolic models (GSMMs) and the associated flux balance analysis (FBA) methods, systematic investigations into the metabolic changes in response to external nutrient alterations, genetic perturbations, and antibiotic treatments become feasible [16–24]. Several studies used transcriptomics data as constraints to compute condition-specific metabolic flux changes in response to antibiotic treatments in MDR bacteria, including *Acinetobacter baumannii* [25], *Mycobacterium tuberculosis* [26], and *Yersinia pestis* [27]. For *P. aeruginosa*, 4 GSMMs have been constructed, iMO1056 [28], Opt208964 [29], iMO1086 [30], and the latest iPae1146 [31]. iMO1056, Opt208964, and iPae1146 used SEED metabolite and reaction names; iMO1056 and Opt208964 are fully accessible via Model SEED [29, 31, 32]; iMO1086 used different identifiers (IR/RR plus 5 digits for reactions and C/EC plus 4 digits for metabolites) [30]. The previous applications of these models have included simulating the metabolic dynamics in cystic fibrosis patients [33], elucidating the mechanisms of biofilm formation [34, 35], predicting potential drug targets [36–38], and identifying the key genes that control virulence factors [31]. As important as they have been, these models have several overarching limitations. Those past models do not include a major cellular component, the periplasmic space; have poor representation of glycerophospholipid (GPL) biosynthesis; and lack lipid A modification reactions. Considering the pathogenesis of *P. aeruginosa*, these major limitations significantly compromise the modeling functions. In particular, the power of the 4 reported GSMMs to predict metabolic responses to antibiotic treatment is very limited, as periplasmic GPL and LPS biogenesis plays critical roles in responses to anti-pseudomonal antibiotics such as polymyxins [15, 39–42].

Here, we describe *i*PAO1, a newly developed GSMM for *P. aeruginosa* PAO1 based upon Opt208964 [29] and iMO1056 [28] but with intensive manual curation using several major databases and the literature. Most notably, *i*PAO1 is the first GSMM for *P. aeruginosa* where the periplasmic space compartment is incorporated to comprehensively represent cross-membrane transport, GPL metabolism, and LPS biosynthesis. To the best of our knowledge, *i*PAO1 represents the most comprehensive metabolic reconstruction for *Pseudomonas* thus far. Modeling with *i*PAO1 revealed that the lipid A modifications might exert limited impact on cell growth and metabolism but change the physiochemical properties of bacterial OM. Constrained by gene expression levels, the model was used to elucidate the metabolic responses to polymyxin B treatment. Together, *i*PAO1 provides a powerful systems platform for antimicrobial pharmacological research to combat the rapidly increasing resistance.

## Data Description

The genome sequence and annotation of *P. aeruginosa* PAO1 were obtained from GenBank (accession NC_002516.2). Models iMO1056 and Opt208964 were retrieved from Model SEED [32]. The gas chromatography–mass spectrometry (GC-MS) metabolomics data were collected from the literature [43]. Metabolites, reactions, and pathways were obtained from databases KEGG (Kyoto Encyclopaedia of Genes and Genomes) [44], MetaCyc [45], TCBD (Transporter Classification Database) [46], TransporterDB [47], and Pseudomonas Genome DB [48]. Growth phenotypes on 190 carbon sources and 95 nitrogen sources were determined using BIOLOG Phenotypic Microarrays. Nonessential gene lists were collected from 2 previously reported transposon mutant libraries for PAO1 [49, 50]. Lipid A of wild-type *P. aeruginosa* PAK was extracted using a mild acid hydrolysis method, and the structural analysis of lipid A was conducted using mass spectrometry [42]. RNA was extracted and used to construct cDNA libraries for RNA-sequencing (RNA-seq) on the Illumina MiSeq platform [51]. The raw reads were quality trimmed and aligned to the PAO1 reference genome using SubRead [52]. Counts were normalized, and the differential gene expression was determined using voom/limma packages with Degust [53]. Whole-cell lipids and intracellular metabolites were extracted and analyzed using liquid chromatography–mass spectrometry [14, 42]. Raw lipidomics and metabolomics data were processed with IDEOM software followed by bioinformatic analysis [54].

## Materials and Methods

### Strain, media, and BIOLOG experiments

*Pseudomonas aeruginosa* PAO1 was cultured in LB media and subcultured on nutrient agar. Cells were swapped into sterile capped tubes containing 16 ml IF-0 solution (Cell Biosciences, West Heidelberg, Australia) until the turbidity achieved 42% transmittance in a Turbidimeter (Pacificlab, Blackburn, Australia). The cell suspension was then diluted 5 times with IF-0 solution and dye (Cell Biosciences) to final 85% transmittance. BIOLOG PM 1–3 (Cell Biosciences) were used to investigate the carbon and nitrogen utilization with 2 independent biological replicates. Sodium succinate was used as the carbon source for examining nitrogen utilization. Growth was detected after 24-h incubation at 37°C, using an Infinite M200 microplate reader (Tecan, Mannedorf, Switzerland) at 595 nm. Readings that were ≥1.5-fold of the negative control (i.e., growth media without bacteria) indicated the utilization of nutrients.

### Development of a GSMM for *P. aeruginosa* PAO1

To expedite model development, 2 curated models for PAO1 with the same identifier systems from Model SEED [32], iMO1056 [28], and Opt20896434 [29] were merged. Databases including KEGG [44], MetaCyc [45], and Pseudomonas Genome DB [48] and the literature were used to complete the model with missing components. The identifiers of metabolites and reactions were kept consistent with Model SEED [29] and cross-referred to MetaCyc, KEGG, PubChem [55], ChEBI [56], ChemSpider [57], and BiGG [58]. The PAO1 genome annotation from Pseudomonas Genome DB [48] was used to construct “gene to protein to reaction” associations [59]. A periplasm compartment was incorporated into the model. Reactions and metabolites were then assigned to cytoplasm, periplasm, and external environment according to the localization prediction of metabolic enzymes by PSORTb 3.0 [60]. Transport reactions were generated to enable material exchange across membranes according to TCBD [46] and TransporterDB [47]. The model was constructed using the Systems Biology Markup Language [61, 62]. VANTED [63] was used for visualization and analysis of the metabolic network. For each metabolite in the model, specific features, including compartment localization, mass, charge, formula, formation free energy, database identifiers, and source, were added (Additional file 14). Each reaction entered into the model was checked with elementary and charge balance. Reversibility was determined first from the primary literature for each particular enzyme or reaction, if available. Further curation on reaction reversibility and directions was conducted based on change of free energy and knowledge about the physiological direction of a reaction in a pathway.

The Gapfind function from the COBRA toolbox [64] was used to identify the isolated and dead-end metabolites in the model. Candidate reactions from KEGG, MetaCyc, and BiGG were manually inspected for relevance and homology evidence using BLASTp; reactions catalyzed by homologous enzymes (E-value <1 × 10^−5^, identity ≥35%, coverage ≥50%) were added to the model to eliminate the gaps. Mispredictions of BIOLOG growth phenotypes were used to refine the draft model (*i*PAO1_draft2). Further curation was performed to represent the complex biosynthesis pathways of macromolecules (e.g., peptidoglycan, GPL, and LPS).

The biomass formation equation that consisted of the necessary building blocks for bacterial growth was created using the one from iMO1086 [30], with slight modifications on compositions of ions, peptidoglycans, GPL, and LPS (Additional file 17). The growth- and nongrowth-associated maintenance were from iMO1086 [30].

### Growth prediction in BIOLOG media

*i*PAO1 was used to predict the growth phenotypes on chemically defined media with 190 carbon and 95 nitrogen sources (Additional file 18) using the FBA method [24]. The objective function of biomass formation was maximized with the specific nutrient uptake rate set at 10 mmol ⋅ gDW^−1^ ⋅ h^−1^ under aerobic condition, as follows:

\begin{equation*}\begin{array}{@{}*{1}{c}@{}} 
{{\rm{max\ }}{v_{{\rm{biomass}}}}}\\ 
{s.t.{{\bf Sv}} = 0}\\ 
{{a_i} \le {v_i} \le {b_i}, i = 1,2, \cdots ,n} 
\end{array}
\end{equation*}

where *v*_biomass_ denotes the biomass formation flux and S represents the stoichiometric matrix; each metabolic flux *v_i_* was constrained by lower and upper bound *a_i_* and *b_i_*, respectively. All modeling procedures were performed with the COBRA toolbox [64] in MATLAB. The calculated specific growth rates *v*_biomass_ were then compared to the BIOLOG PM data to assess the prediction accuracy using Fisher's exact test.

### Gene essentiality prediction

*In silico* single-gene deletion was performed using the COBRA toolbox; then, the mutant models were used to predict the specific growth rate in LB broth [32] using FBA. Genes with 99% reduction of the specific growth rate relative to the wild type were defined as essential for cell growth; otherwise, they were considered as semi-essential (1–99% reduction) and nonessential (<1% reduction). Two existing PAO1 transposon insertion mutant libraries, 2-allele mutant library [50, 65] and mini-Tn5 insertion mutant library [49], were used to assess the overall prediction accuracy with Fisher's exact test. To determine polymyxin-specific essential genes, transcriptomic constrains were imposed (below) before conducting *in silico* single-gene deletion simulations. The calculated essential genes identified in polymyxin treatment alone but not in the control were considered as polymyxin specific.

### Simulation of bacterial growth and metabolic phenotype changes in response to lipid A modifications

The LPS stoichiometric coefficients in the biomass formula under the control and lipid A modification conditions were set according to the measured lipid A compositions in the wild-type *P. aeruginosa* PAK in the absence and presence of polymyxin B treatment (Table 1) [14]. Aerobic growth was simulated on minimal media with glucose uptake at 10 mmol ⋅ gDW^−1^ ⋅ h^−1^. For each simulation, the solution space was sampled with 10000 random points using the ll-ACHRB algorithm [66]. Flux samples of the control and lipid A modification were then compared. Significantly perturbed metabolic fluxes were identified using a *Z*-score based approach [67].

**Table 1:** Lipid A composition (%) in the outer leaflet of the OM in PAK [14]

Lipid A species	Control	Polymyxin B treated
Hexa-lipid A	42.5 ± 0.46	11.7 ± 1.13
Penta-lipid A	57.5 ± 0.46	67.7 ± 3.16
L-Aminoarabinosylated hexa-LA	0	1.24 ± 0.31
L-Aminoarabinosylated penta-LA	0	19.4 ± 3.44
Total	100	100

To further analyze the metabolic impact of lipid A modifications, the proportions of all types of LPS in the biomass formula were randomly assigned and the process was repeated 1000 times. For each repetition, the specific growth rates were calculated and solution space was sampled using the methods described above. For each type of lipid A, specific physiochemical properties (*f*) including total atom number, partition coefficient (logP), average charge, and molecular polarity were predicted at pH 7 using the cxcalc tool from ChemAxon (Budapest, Hungary). The overall apparent properties *F* of the OM were estimated by calculating the weighted sum, as follows:

\begin{equation*}F\ = \mathop \sum \limits_{j = 1}^n {w_j}{f_j}\
\end{equation*}

where *w_j_* represents the stoichiometric coefficient of the *j*-th of 288 heterogeneous LPS molecules in the biomass formula. Pairwise correlation analysis was conducted between lipid A modifications, physiochemical properties changes, bacterial growth, and metabolism alterations.

### Predict metabolic responses to polymyxin treatment by constraining fluxes with transcriptomics data

The RNA-seq data from 1-h 1 mg·L^−1^ polymyxin B treatment experiment using PAO1 were used as flux constraints for modeling [51]. For each gene under every condition, the reads per kilobase million (RPKM) value was calculated from the aligned reads using the edgeR package [68] and normalized to constrain flux upper bounds (*b_i_*) using the E-Flux algorithm [26]. Specifically, for each reaction catalyzed by a single enzyme, the upper flux bound was set to the determined RPKM value under the respective condition. For a reaction catalyzed by an enzyme complex, the upper bound was set to the minimum RPKM value of the associated genes. For a reaction catalyzed by isozymes, the upper bound was set to the sum of RPKM values of the associated genes. The maximum of upper bounds was then normalized to 10000 mmol ⋅ gDW^−1^ ⋅ h^−1^. The lower bounds *a_i_* were set to 0 for irreversible and −*b_i_* mmol ⋅ gDW^-1^ ⋅ h^−1^ for reversible reactions, respectively. CAMHB was used in the RNA-seq experiment; it is known as an undefined medium that contains mainly amino acids and oligopeptides [69]. The maximum uptake rates of amino acids in *P. aeruginosa* vary between 0.26 and 1.44 mmol ⋅ gDW^−1^ ⋅ h^−1^ [70, 71, 73]. Therefore, the upper bounds $(b_i^{{\rm{CAMHB}}})$ of uptake rates of amino acids, vitamins, and dipeptides in *i*PAO1 were constrained to 1 mmol ⋅ gDW^−1^ ⋅ h^−1^ without loss of generality. For each condition, the solution space was sampled with 10000 points using ll-ACHRB as described above. Statistical significance of differential flux distributions was computed using the *Z*-score method described above. The turnover rate for key metabolites was calculated by summing up all influxes or effluxes [74]. To assess the impact of changing nutrient uptake bounds, sensitivity analysis was conducted by randomly sampling solution space as above while varying $b_i^{{\rm{CAMHB}}}$ from 0.26 to 1.44 mmol ⋅ gDW^−1^ ⋅ h^−1^.

## Results

### Development of a superior GSMM for *P. aeruginosa* PAO1

Initially, a draft model (*i*PAO1_draft1) containing 1991 reactions, 1579 metabolites, and 1021 genes was created based upon iMO1056 [28] and Opt208964 [29] (Additional files 2–4). To obtain a high-quality GSMM, extensive manual curation was conducted. First, *i*PAO1_draft1 was complemented using databases and the literature. Specifically, the following additional information was incorporated into the draft model, 285 metabolites and 36 reactions from KEGG [44], 225 metabolites and 50 reactions from MetaCyc [45], and 7 metabolites and 20 reactions obtained by previous GC–MS-based quantification [43] (Additional files 5 and 6).

Second, a periplasmic compartment was built to incorporate 698 periplasmic metabolites, 509 transport reactions across the inner membrane (IM), 441 transport reactions across the outer membrane (OM), and 403 periplasmic reactions. The resulting intermediate model was designated as *i*PAO1_draft2.

Third, the major pathway gaps were filled. GapFind [75] identified 109 dead-end metabolites (Additional file 7). The growth phenotypes on 190 carbon and 95 nitrogen nutrients were predicted using *i*PAO1_draft2, and compared with our experimental BIOLOG phenotypic microarray (PM) results (Additional file 8). As a result, 162 false-negative predictions (i.e., the prediction indicated nongrowth whereas the BIOLOG experiment demonstrated valid growth on a specific nutrient) were determined, indicating the lack of associated transport or catabolic reactions for these nutrients. To link the dead-end metabolites back to the metabolic network and eliminate inconsistencies with the BIOLOG PM results, several modifications were made, including adjustment of the reversibility settings of 180 reactions and change in the directions of 87 reactions (Additional file 9); removal of 14 metabolites and 96 reactions (Additional files 10 and 11), which were either duplicated (e.g., β-D-glucose was duplicated with D-glucose) or representing general metabolite classes (e.g., protein, mRNA, DNA); and addition of 98 boundary reactions, 677 transport reactions, and 252 metabolic reactions (Additional file 12). Resolving the false-negative predictions of the BIOLOG growth phenotypes substantially improved the model. For example, predictions using *i*PAO1_draft2 showed that PAO1 was unable to grow with formic acid as a sole carbon source due to lack of the corresponding transport reaction. Interrogation of the Pseudomonas Genome Database [48] and Pfam [76] identified PA2777, a hypothetical protein in the National Center for Biotechnology Information (NCBI) and UniProt that may encode formic/nitrite transporter (Pfam01226, *P* = 7e-34). Subsequent addition of the transport reaction (rxn08526) enabled *in silico* growth of PAO1 on formic acid. Another example is that initially *i*PAO1_draft2 failed to predict utilization of 1,2-propanediol for growth owing to the exiting gap in dehydrogenation of 1,2-propanediol to lactaldehyde. Using the Basic Local Alignment Search Tool for Proteins (BLASTp) with the query sequence of lactaldehyde reductase (*fucO, b2799*) from *Escherichia coli* K12 MG1655, we identified a candidate homologous gene PA1991 (Identity = 35%, Eval = 2e-75, BLASTp). PA1991 encodes an iron-containing alcohol dehydrogenase and has more than 300 orthologues in Gram-negative bacteria that encode lactaldehyde oxidoreductases or 1,2-propanediol dehydrogenases according to OrthoDB [77]. Inactivation of PA1991 resulted in an 8-fold prolonged lag phase when *P. aeruginosa* grew on 1,2-propanediol [78]. Therefore, reaction rxn01615 oxidizing 1,2-propanediol to lactaldehyde was added into *i*PAO1_draft2. A very large number of such labor-intensive manual curations were conducted to improve the model. This enabled *in silico* growth on a number of nutrients from BIOLOG PMs, including 4-hydroxyphenylacetate, tyramine, quinic acid, itaconic acid, citramalic acid, L-pyroglutamic acid, carnidine, glycinebetaine, L-methylsuccinate, and D-amino acids (Additional file 8).

**Figure 1:**
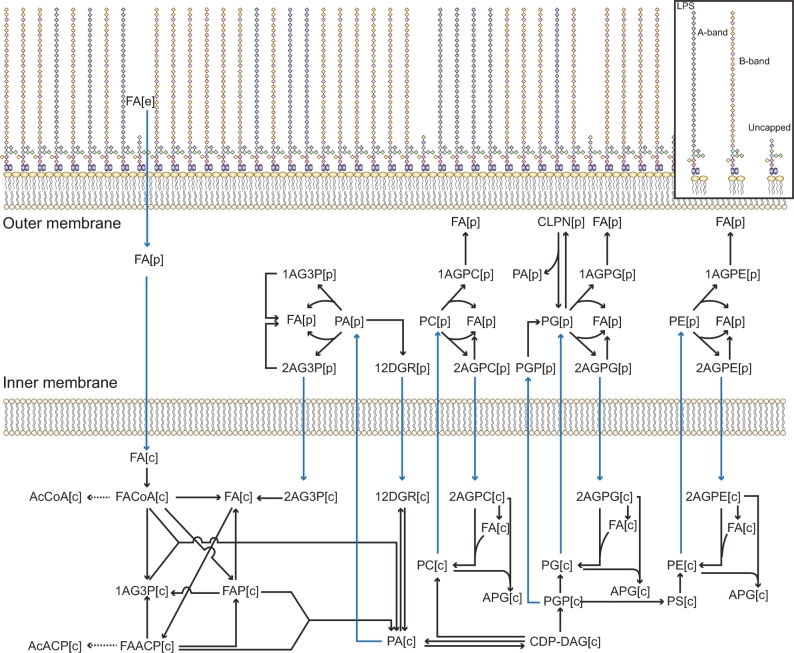
The curated GPL biosynthesis in *i*PAO1. [c], intracellular metabolites; [p], periplasmic metabolites; [e], external metabolites. Blue arrows indicate transport reactions. Full names of metabolite classes are listed in Additional file 27.

**Figure 2:**
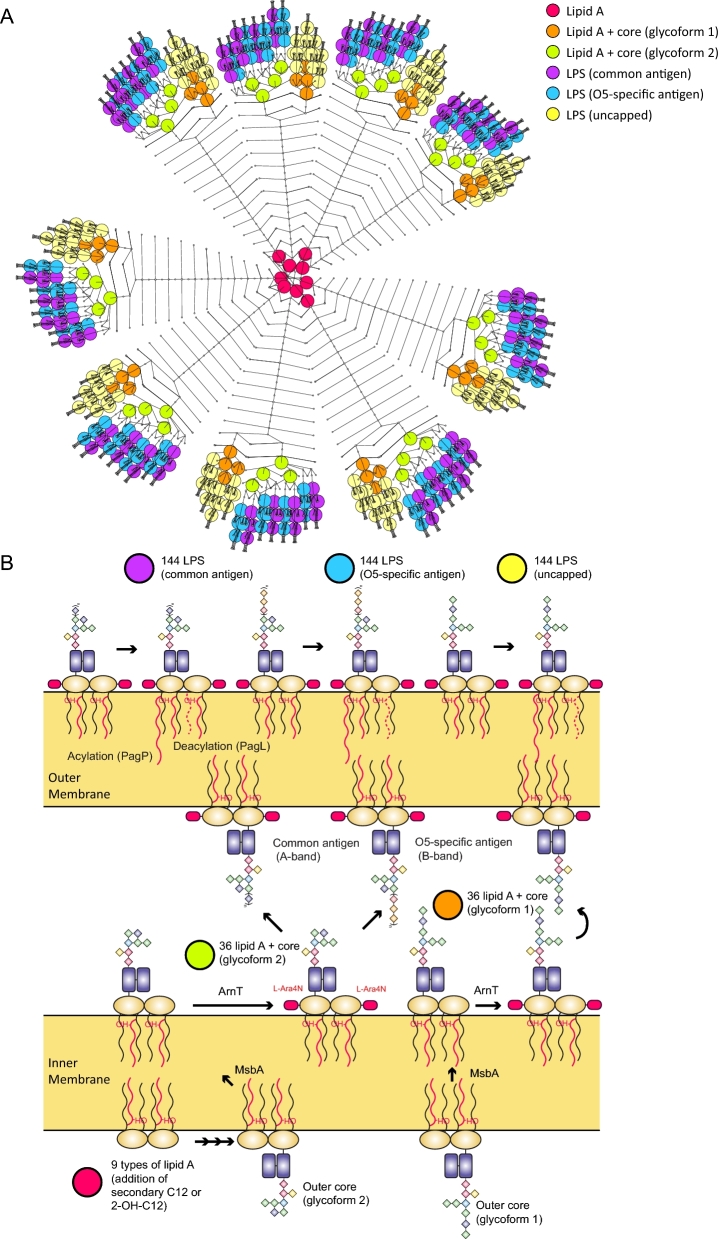
LPS biosynthesis and modification in *i*PAO1. A) VANTED diagram showing the biosynthesis of different LPS molecules. B) LPS biosynthesis pathway; lipid A and LPS are indicated in the same color as in (A).

Fourth, the biogenesis of the bacterial envelope was delineated. Cross-linking between amino acids residues among peptidoglycan chains results in a rigid network structure in *P. aeruginosa* [79]. In total, 17 reactions representing peptidoglycan cross-linking and hydrolysis were incorporated by searching for homologues of glycosyltransferases, transpeptidases, carboxypeptidases, and endopeptidases in PAO1 [80]. Overall, a detailed peptidoglycan biosynthesis pathway was constructed with 60 reactions. GPL compositions in the bacterial membranes can change in response to antibiotic treatment [39, 81]. Previous studies [82] and our own lipidomics results [14] showed a great diversity in GPL species in *P. aeruginosa*. Overall, 386 unique metabolites (i.e., 66.2% of the 583 metabolites in the GPL metabolism pathway) and 367 reactions (66.7% of the 550 reactions in the GPL metabolism pathway) were incorporated into *i*PAO1_draft2 (Additional files 1, 13, and 14; Fig. 1). LPS consists of lipid A, core oligosaccharide, and *O*-antigen polysaccharide [40] and plays key roles in the host–pathogen interaction and resistance to antibiotics such as polymyxins [13, 83]. A detailed synthesis and interconversion network was generated with 432 types of LPS and 1169 reactions (Fig. 2; Additional file 1). Notably, our GSMM is the most comprehensive to date in lipid A biosynthesis and modifications.

**Figure 3:**
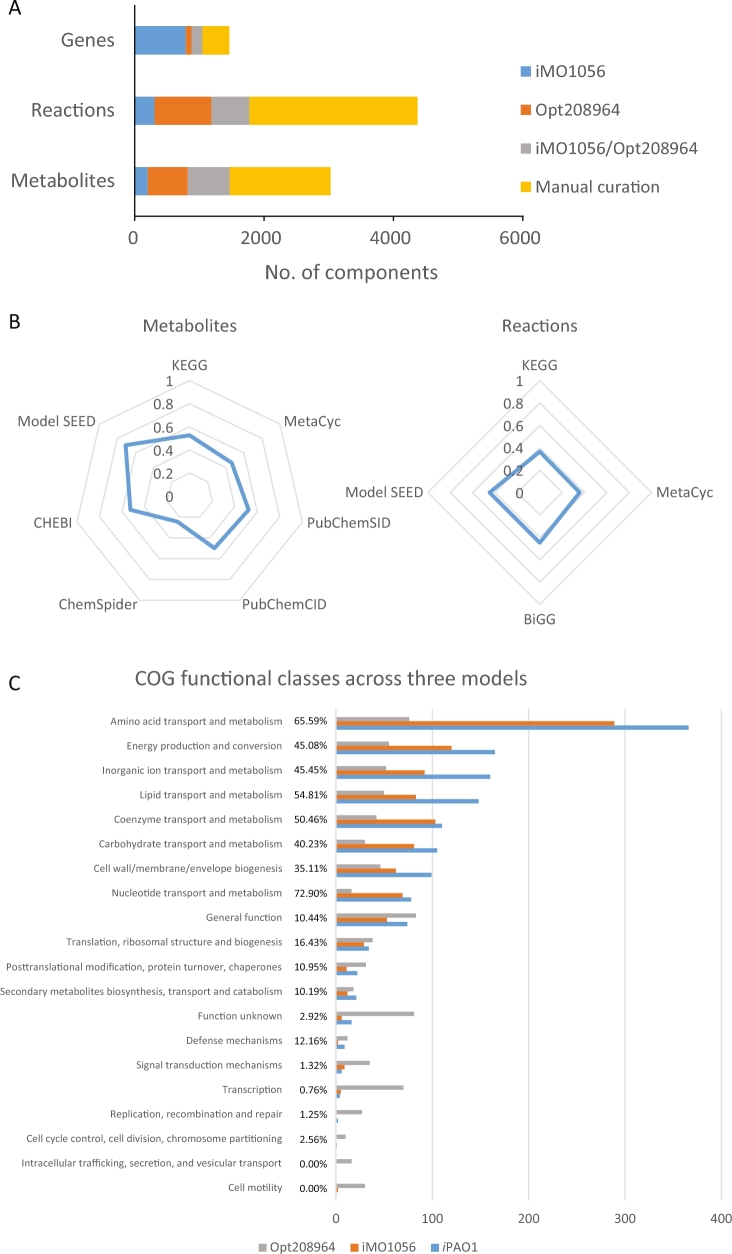

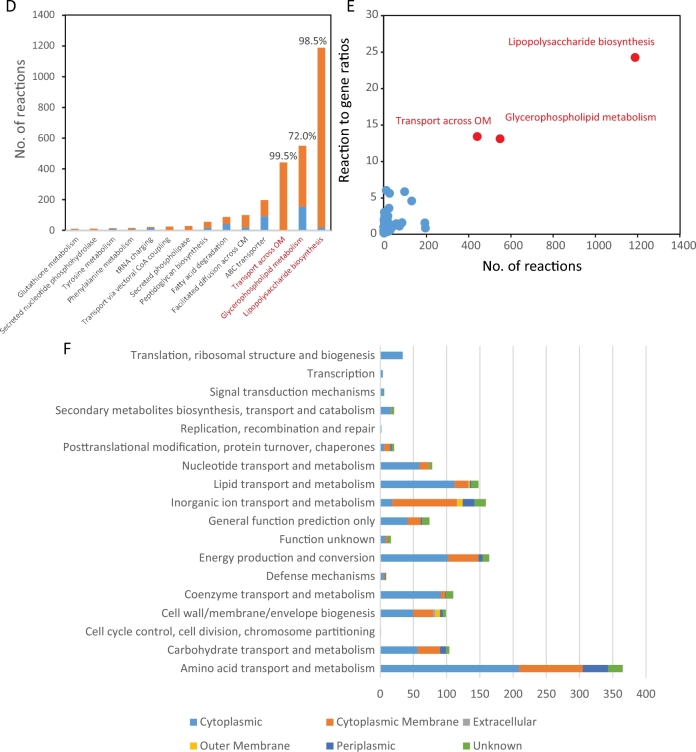
Constitutional genes, reactions, and metabolites in *i*PAO1. A) Sources of *i*PAO1 components. B) Radar map showing the percentages of metabolites and reactions with valid database identifiers. C) The COG functional classification of the involved genes in iMO1056, Opt208964, and *i*PAO1. Percentages given in the middle indicate the coverages of COG groups. The proportions of the curated reactions (D), reaction-to-gene ratio (E), and predicted subcellular localizations of the involved proteins (F) are shown for each pathway or COG group. In panel D, red bars indicate the curated reactions; blue bars indicate the reactions from the previous model. In panels D and E, pathways with the highest curation proportion or reaction-to-gene ratio are highlighted in red.

The resulting final *i*PAO1 model consists of 3022 metabolites, 4365 reactions, and 1458 genes (25.8% of the PAO1 genome; Additional files 15–17), representing, respectively, 252%, 340%, and 40% increases of the components in iMO1056 and 125%, 171%, and 43% increases of the components in Opt208964 (Table 2). The significant expansion in *i*PAO1 includes cross-membrane transport, GPL/LPS biosynthesis, peptidoglycan biosynthesis, and fatty acid degradation (Additional files 15–17). The reactions from *i*PAO1 were categorized into 109 pathways mainly based on classifications in MetaCyc and KEGG. In *i*PAO1, 27.9%/43.7%/51.6% metabolites, 20.3%/33.5%/59.5% reactions, and 65.3%/17.6%/28.5% genes are originated from iMO1056, Opt208964, and our manual curation, respectively (Fig. 3A).

**Table 2:** Components in model iMO1056, Opt208964, and *i*PAO1

Class	Subclass	*i*PAO1	iMO1056	Opt208964
Genes		1458	1042	1021
Reactions		4365	992	1609
	Cytoplasmic metabolic reactions	1716	730	1132
	Periplasmic metabolic reactions	403	0	0
	External metabolic reactions	40	0	0
	Transport reactions	960	150	253
	Transport across IM	519	0	0
	Transport across OM	441	0	0
	Transport from cytoplasm to extracellular space	0	150	253
	Boundary reactions	352	112	223
	Reactions without associated genes	628	159	374
	Sink reactions	0	0	1
Metabolites		3022	858	1344
	Cytosol	1519	746	1121
	Periplasm	698	0	0
	Extracellular space	805	112	223
Pathways		109	-^a^	117

^a^Pathway information is not available in iMO1056 from the Model SEED database.

Components in *i*PAO1 were aligned with databases including KEGG [44], MetaCyc [45], PubChem [55], ChemSpider [57], ChEBI [56], Model SEED [32], and BiGG [58] (Additional files 15 and 16). Consequently, 1404 (46.5%), 1590 (52.6%), and 2142 (70.9%) metabolites have corresponding identifiers in MetaCyc, KEGG, and Model SEED, respectively; 1556 (35.6%), 1596 (36.6%), and 1964 (45.0%) reactions were computationally mapped to the reactions from MetaCyc, KEGG, and Model SEED, accordingly (Fig. 3B). A significant portion of mismatches were caused by the incorporation of specific types of metabolites in the GPL metabolism and LPS biosynthesis pathway, which in databases are usually lumped as general compound classes. The properties of metabolites, including mass, charge, and formula, were included in *i*PAO1. The standard Gibbs free energy changes of formation (*Δ_f_G°*) and reaction (*Δ_r_G°*) were obtained from MetaCyc and Model SEED for 1877 metabolites (62.1%) and 1355 reactions (31.0%) (Additional files 15 and 16).

A breakdown of genes involved in *i*PAO1 (Additional file 17) using the clusters of orthologous groups (COGs) showed remarkable improvement compared to previous reconstructions (Fig. 3C). The largest increase in the coverage compared to iMO1056 is lipid transport and metabolism (24.1%), followed by inorganic ion transport and metabolism (19.3%); whereas compared to Opt208964, the largest increase in the coverage is nucleotide transport and metabolism (57.9%), followed by amino acid transport and metabolism (52.0%). Overall, the transport and metabolism of nucleotides and amino acids showed the highest percent coverage of COG functional categories in *i*PAO1 (72.9% and 65.6%, respectively). Notably, the reactions in categories not apparently related to metabolism were dramatically reduced in *i*PAO1 compared to Opt208964, including translation, ribosomal structure and biogenesis, post-translational modification, protein turnover, chaperones and signal transduction mechanisms, and undetermined categories, including function unknown class.

In *i*PAO1, GPL metabolism, LPS biosynthesis, and transport across OM were ranked the 3 largest pathways and also contained the largest proportion of curated reactions (Fig. 3D). Additionally, these 3 pathways have high reaction-to-gene ratios (13.1–24.2; Fig. 3E), indicating that enzymes in these pathways are capable of acting on a broad range of substrates. As kinetic parameters are usually not involved in a GSMM, constraint-based analyses (e.g., FBA) of a GSMM do not directly account for enzyme levels, intracellular metabolic concentrations, or substrate-level regulation. Accordingly, the affinity difference of various substrates was not considered in our *i*PAO1 modeling effort.

We used the biomass formation equation from iMO1086 to construct *i*PAO1 with modifications on LPS and ion species (Additional file 18). In addition, to take into account the extra energy consumption caused by charging tRNAs, the original amino acids in the biomass formation reaction were replaced by aminoacyl-tRNA, followed by addition of specific charging reactions to the model. Taken together, *i*PAO1 represents the most comprehensive metabolic reconstruction thus far for *P. aeruginosa* PAO1.

### Growth capability on various nutrients

**Figure 4:**
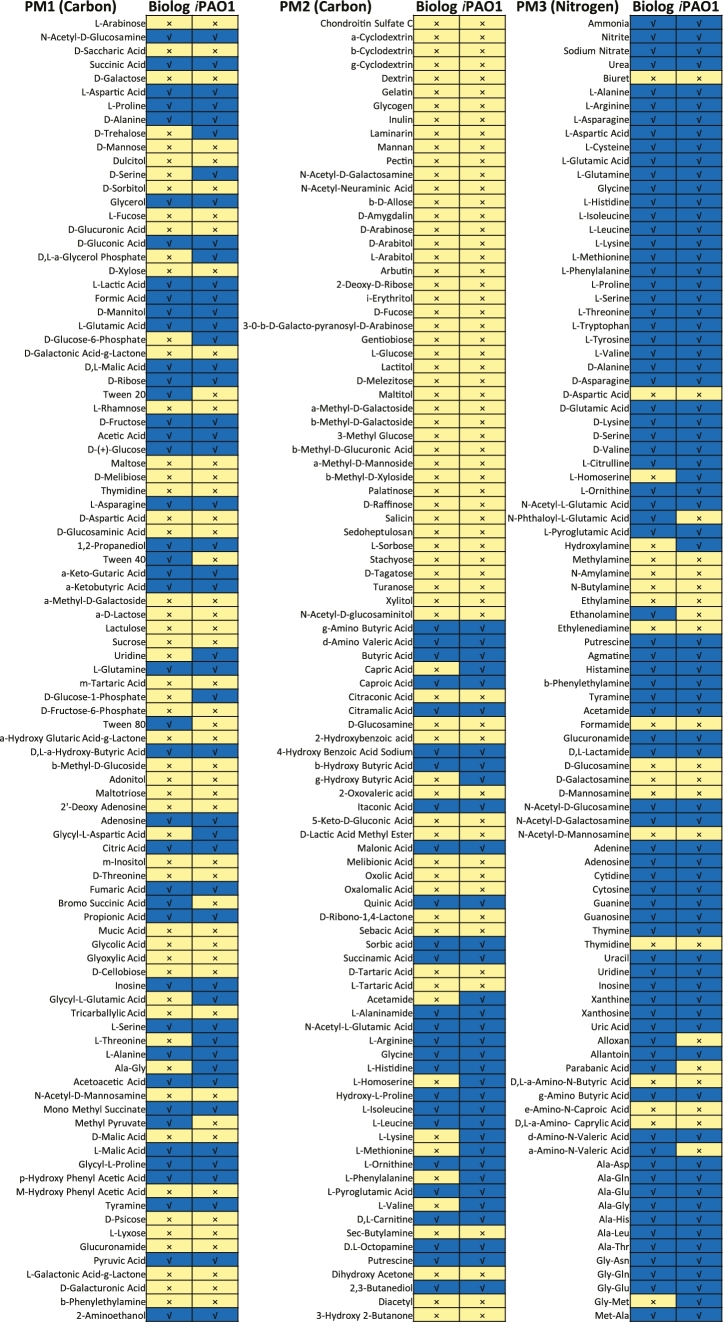
Comparison of the BIOLOG result (left columns) and model prediction (right columns). Blue indicates growth and yellow indicates no growth.

Investigation of nutrient utilization using BIOLOG PMs showed that PAO1 could utilize a broad range of nutrient sources, indicated by the observed growth on 68 of 190 (35.8%) carbon and 76 of 95 (80.0%) nitrogen substrates (Fig. 4). Growth simulation with *i*PAO1 achieved an overall accuracy of 89.1% (254 of 285), which substantially outperformed previous models (81.5% for Opt208964 [29], 77.9% for iMO1056 and iMO1086 [30], and 80% for iPae1146 [31]). Twenty-one false-positive and 10 false-negative (Fig. 4, Additional file 8) disagreements were observed, possibly due to the complexity of regulatory mechanisms and missing annotation of nutrient transport and/or catabolism pathways in PAO1.

### Prediction and validation of gene essentiality

*In silico* single-gene deletion with *i*PAO1 showed 143 essential genes (*μ*_mut_ < 0.01 *μ*_wt_), 40 semi-essential genes (0.01 *μ*_wt_* < μ*_mut_ < 0.99 *μ*_wt_), and 1275 nonessential genes (0.99 *μ*_wt_* < μ*_mut_< *μ*_wt_) when growing in Luria-Bertani (LB) media (Additional file 19). Among the essential metabolic genes, the largest COG proportion (46 of 143, 32.1%) is cell envelope biogenesis, indicating that there are relatively fewer alternative reactions in this pathway. For nonessential genes, amino acid transport and metabolism (352 of 1315, i.e., 26.7%) represents the largest group, suggesting the existence of large metabolic redundancy.

The predicted gene essentiality was further verified by 2 independent genome-scale transposon mutant libraries [49, 50, 65]. The overall prediction accuracy achieved 87.9%, which is higher than iMO1056 (85.0%) [28] and iMO1086 (84.2%) [30] but slightly lower than Opt208964 (92.9%) [29] and iPae1146 (91.46%) [31]. The higher accuracy in Opt208964 is partially due to errors in the annotation of essential genes. For instance, 351 genes in Opt208964 were grouped as experimentally validated essential; however, 145 of the 351 genes are nonessential as their corresponding mutants were found in the transposon mutant library [50]. In iPae1146, removal of 16 isozymes increased the prediction accuracy of essential genes; e.g., 3-ketoacyl-ACP reductase (EC 1.1.1.100) reactions in iPae1146 were associated with PA2967 only [31], whereas in *i*PAO1, these reactions were associated with another 8 highly conserved isozymes (PA0182, PA1470, PA1827, PA3387, PA4089, PA4389, PA4786, and PA5524). Furthermore, condition-specific essential genes were predicted in *i*PAO1 by imposing transcriptomics constraints. Modification of lipid A with 4-amino-4-deoxy-L-arabinose (L-Ara4N) leads to polymyxin resistance in *P. aeruginosa*, and deficiency in *arn* genes reverses the susceptibility [84]. Seven additional essential genes (*arnABCDEFT*, PA3552-3558, encoding L-Ara4N biosynthesis) were predicted by *i*PAO1 under polymyxin treatment.

### Impact of lipid A modifications on bacterial growth and metabolism

*Pseudomonas aeruginosa* modifies lipid A components in the OM in response to polymyxin treatment [85]. The LPS stoichiometric coefficients in the biomass formula of *i*PAO1 were configured based on our lipid A profiling data [14] (Table 1), and the metabolic impact of lipid A modifications was predicted by randomly sampling the metabolic solution space with 10000 points (see Methods section). Overall, 273 fluxes were significantly affected (*Z*-score, false discovery rate [FDR] <0.01; >0.1 mmol ⋅ gDW^−1^ ⋅ h^−1^ under at least 1 condition; Additional file 20). The specific growth rate remained unchanged. A 0.026 mmol ⋅ gDW^−1^ ⋅ h^−1^ flux from glucose via glucose 6-phosphate, uridine diphosphate glucose, and consequently L-Ara4N biosynthesis was identified due to lipid A modifications. The overall fluxes through lipid A deacylation reactions were increased (from 0.007 mmol ⋅ gDW^−1^ ⋅ h^−1^ to 0.011 mmol ⋅ gDW^−1^ ⋅ h^−1^); the generated (*R*)-3-hydroxydecanoate was fuelled into β-oxidation to produce octanoyl-CoA, which was subsequently salvaged for fatty acid biosynthesis.

**Figure 5:**
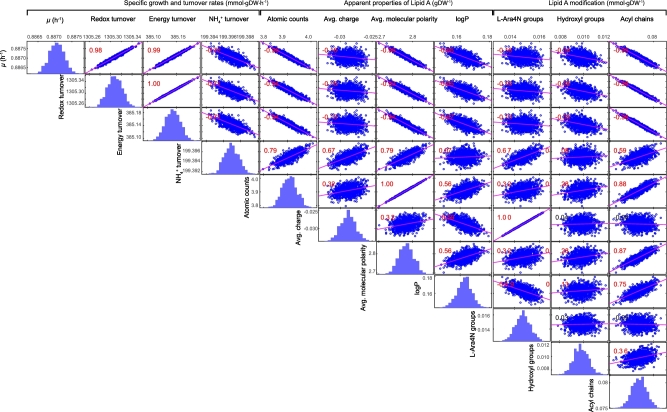
Simulation of the impact of lipid A modifications on bacterial growth, metabolism, and OM physiochemical properties. The significant correlation (*P* < 0.05) of paired items is indicated in red.

To further investigate the impact of lipid A modifications on bacterial growth, 1000 sets of the compositions of 288 heterogeneous LPS molecules were randomly generated with the total proportion of LPS unchanged in the biomass formation formula (Additional file 21). The metabolic fluxes were calculated for each of the 1000 sets of LPS compositions by randomly sampling the solution space with 10000 points. Across the 1000 sets of metabolic fluxes (Additional file 23), the specific growth rate varied between 0.8812 and 0.8897 mmol ⋅ gDW^−1^ ⋅ h^−1^. Correlative analysis of the apparent overall physiochemical properties of lipid A (Additional file 22) with the predicted growth phenotypes showed 3 interesting findings. First, addition of L-Ara4N reduced the negative charge of lipid A (*ρ* = 1.00), decreased the hydrophobicity of the OM (represented by logP, *ρ* = −0.59) but required assimilation of more ammonia (represented by ammonia turnover, *ρ* = 0.57). Second, hydroxylation on acyl chains of lipid A exerted minor effects over either bacterial growth or physiochemical properties. Third, addition of acyl chains resulted in large lipid A molecules (represented by the atomic counts, *ρ* = 0.88), enhanced molecular polarity of lipid A (*ρ* = 0.87), increased OM hydrophobicity (*ρ* = 0.75), and, notably, retarded growth (*ρ* = −0.95), reduced redox and energy turnover (*ρ* = −0.98 for both), and increased requirement of ammonia (*ρ* = 0.59) (Fig. 5). It is evident that none of the three aforementioned modifications produced a dramatic impact on bacterial growth or metabolism (Additional file 23).

### Elucidating the mechanisms of metabolic responses to polymyxin treatment

RNA-seq data were utilized as model constraints (Additional file 24) with an E-Flux method [67] to calculate the metabolic fluxes in the absence and presence of polymyxin B (see the Methods section). The exchange fluxes were constrained based on the maximum uptake rates of the media ingredients (see the Methods section and Additional file 1). Comparison of the flux distributions revealed that 1392 reactions were differentially regulated (FDR <0.01, Additional file 25). A range of metabolic pathways were significantly disturbed, including central metabolism, amino acid metabolism, purine biosynthesis, fatty acid biosynthesis and metabolism, LPS and GPL biosynthesis, and transport reactions. Polymyxin B treatment reduced the growth rate (18.2%) and increased oxygen uptake (6.9%) and CO_2_ emission (6.0%); however, the respiration quotient remained roughly unchanged (Table 3).

**Table 3:** Specific growth rate, significantly altered major exchange fluxes (>1 mmol ⋅ gDW^−1^ ⋅ h^-1^), respiration quotient, and the fluxes through F_0_F_1_-ATPase calculated using the RNA-seq data [51] as flux constraints

Exchange flux (mmol ⋅ gDW^−1^ ⋅ h^-1^)	Control	Polymyxin B treatment	*Z-*score	FDR^a^
Specific growth rate (h^−1^)	0.82 ± 0.00	0.67 ± 0.00	10 201.3	0.00
H_2_O	46.9 ± 21.8	53.0 ± 19.0	20.37	0.00
O_2_	−106.0 ± 23.0	−113.4 ± 19.8	24.30	0.00
CO_2_	109.2 ± 22.6	115.8 ± 19.3	22.62	0.00
NH_4_^+^	36.6 ± 9.29	38.0 ± 8.77	10.94	0.00
Glycine	2.15 ± 4.76	1.92 ± 4.46	3.05	0.00
L-Alanine	1.21 ± 5.01	−0.52 ± 2.20	31.77	0.00
Succinate	2.08 ± 4.19	2.52 ± 4.42	7.27	0.00
H^+^	−41.5 ± 14.1	−40.4 ± 11.9	6.44	0.00
Methanethiol	1.53 ± 0.82	1.34 ± 1.11	12.62	0.00
H_2_S	1.66 ± 1.74	1.41 ± 2.18	9.29	0.00
Respiration quotient	1.03 ± 0.10	1.02 ± 0.10	7.63	0.00
ATPase (mmol ⋅ gDW^−1^ ⋅ h^−1^)	−188.6 ± 52.4	−167.6 ± 48.4	29.62	0.00

^a^FDR was calculated using the Benjamini-Hochberg method [72].

**Figure 6:**
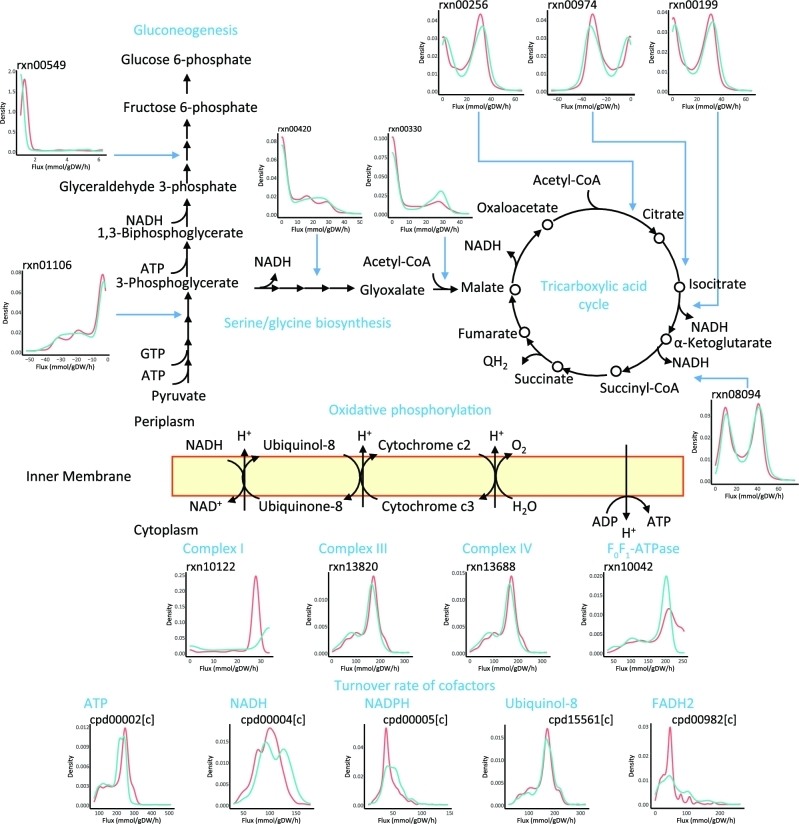
Polymyxin B–induced metabolic perturbations. The distributions of metabolic fluxes and metabolite turnover rates are shown in subgraphs with red indicating control and blue indicating polymyxin B treatment.

As the major carbon sources, the amino acids and oligopeptides from cation-adjusted Mueller-Hinton broth (CAMHB) were utilized to generate intermediate metabolites, redox, and energy equivalents for biomass formation. In response to polymyxin treatment, the gluconeogenesis pathway was significantly induced from pyruvate to 3-phosphoglycerate but suppressed from 3-phosphoglycerate toward glucose 6-phosphate. The extra flux from 3-phosphoglycerate was shunt to serine and glycine biosynthesis (Fig. 6) via 3-phospho-D-glycerate:NAD^+^ oxidoreductase (rxn01101), 3-phosphoserine:2-oxoglutarate aminotransferase (rxn02914), *O*-phospho-L-serine phosphohydrolase (rxn00420), and 5,10-methylenetetrahydrofolate:glycine hydroxymethyltransferase (rxn00692), through which more reduced nicotinamide adenine dinucleotide (NADH) equivalents were generated compared to the control (i.e., growth in CAMHB without polymyxin treatment). The resulting 1-carbon unit in 5,10-methylenetetrahydrofolate was oxidized to formic acid via 10-formyltetrahydrofolate amidohydrolase (rxn00691); the generated glycine was fuelled into tricarboxylic acid (TCA) cycle via glycine:oxygen oxidoreductase (rxn00269) and acetyl-CoA:glyoxylate C-acetyltransferase (rxn00330). In addition, the metabolic flux via TCA cycle was upregulated from citrate to fumarate, with increased NADH production. Within oxidative phosphorylation, the mean fluxes through NADH dehydrogenase (Complex I, rxn10122), cytochrome bc1 complex (Complex III, rxn13820), and cytochrome c oxidase (Complex IV, rxn13688) decreased by 6.6%, 7.2%, and 7.8%, respectively. The flux via F_0_F_1_-ATPase (Complex V, rxn10042) was downregulated by 11.1%. The overall fluxes via biosynthesis of macromolecules including LPS, GPL, and peptidoglycan decreased due to the significantly reduced biomass formation. The biosynthesis of spermidine increased by 38.3% in response to polymyxin treatment that was also indicated by upregulated expression of *speD* (PA4773; encoding the *S*-adenosyl-L-methionine decarboxylase, log_2_FC = 3.62, FDR <0.01) and *speE* (PA4774; encoding spermidine synthase, log_2_FC = 3.54, FDR <0.01).

Calculating the flux-sum of critical cofactors revealed 13.1% increase of redox turnover and 8.2% decline of energy turnover after 1 mg ⋅ L^−1^ polymyxin B treatment for 1 h. Breaking down the cofactors showed that the turnover of major redox equivalents NADH, Reduced nicotinamide adenine dinucleotide phosphate (NADPH), ubiquinol-8, and FADH2 substantially increased by 12.6%, 13.9%, 3.9%, and 35.9%, respectively; whereas the turnover of ATP, the major contributor to energy, significantly decreased by 8.52% after 1 mg ⋅ L^−1^ polymyxin treatment for 1 h (Fig. 6, Additional file 26). Overall, metabolic flux analysis using *i*PAO1 integrated with our transcriptomics data revealed a significant global impact on bacterial metabolism due to polymyxin B treatment.

## Discussion

The emergence of Gram-negative ‘superbugs’ that are resistant to the last-resort polymyxins highlights the urgent need for novel approaches such as GSMMs to understand the mechanisms of antibacterial activity and resistance. The main utility of GSMMs is their ability to bridge critical gaps between genomics and metabolic phenotypes through the prediction of metabolic responses to antimicrobial treatments at the network level. Here, we report the development, optimization, validation, and application of a high-quality GSMM designated *i*PAO1 for a type strain *P. aeruginosa* PAO1. Importantly, *i*PAO1 was used to understand the complicated effect of polymyxin treatment on bacterial metabolism. Simulation with *i*PAO1 showed that lipid A modifications in response to polymyxin treatment only exert minor effects on bacterial growth and metabolism. Further calculations that integrate transcriptomics data as model constraints revealed that polymyxin treatment may reduce growth and affect a broad range of pathways.

*i*PAO1 represents the most comprehensive metabolic model for *P. aeruginosa* to date and incorporates 1458 genes, accounting for approximately 25.8% of the PAO1 genome. Among the 4 GSMMs developed for *P. aeruginosa* PAO1, iMO1086 and iPae1146 were constructed on the basis of iMO1056 with moderate increase of metabolites, reactions, and genes [28, 30, 31]; Opt208964 is also in a medium size, which limits modeling capacity [29]. In contrast, *i*PAO1 is significantly expanded in model scale by doubling or even tripling the numbers of metabolites and reactions (Fig. 3A). *i*PAO1 achieved an unprecedented prediction accuracy of 89.1% for growth on various nutrients, outperforming all of the previously reported GSMMs for *P. aeruginosa* [28–31]. The *i*PAO1 model was also used to predict gene essentiality with a high accuracy of 87.9%. Given the extensive curation and significant expansion, *i*PAO1 will serve as the primary reference for future development of metabolic models, particularly for other *P. aeruginosa* strains.

Unlike *i*PAO1, none of the previous *P. aeruginosa* GSMMs incorporated the periplasm. As polymyxins initially target LPS in the OM and can cause substantial changes in the cell envelope, the periplasmic space is a major component in *i*PAO1. The periplasmic space of *E. coli* is estimated to constitute up to 16% of total cell volume [86]. It contains a thin cell wall composed of peptidoglycan and a variety of ions and proteins, which are involved in transport, folding, cell envelope biogenesis, electron transport, and xenobiotic metabolism [87]. *i*PAO1 is the first *P. aeruginosa* GSMM to incorporate the periplasmic compartment, enabling accurate representation of metabolic machinery, especially for those reactions that occur exclusively in this important cellular space, and transport of substrates across the IM and OM. Furthermore, *i*PAO1 provides detailed representations of GPL and LPS biosynthesis that allows the precise mapping of GPL and LPS responses from experimental metabolomics and lipidomics data (Fig. 1 and 2).

In response to polymyxin treatment, Gram-negative bacteria modify their lipid A with cationic moieties (i.e., phosphoethanolamine and L-Ara4N) that act to repel the like-charge of the polymyxin molecule [40]. Based on our simulations (Additional file 20), we purport that such lipid A modifications exerted a limited impact on cellular metabolism and growth. Most of the flux changes were insignificant; the remaining significant flux changes mainly resulted from futile cycles that contain sets of reactions using redox equivalents, whereas the net carbon flow remained unchanged. Simulation using randomized lipid A compositions further consolidated our hypothesis that lipid A modifications cause only moderate variations of bacterial growth and metabolism (Fig. 5, Additional file 23). Our simulation results revealed that lipid A modifications result in substantial physiochemical changes in the OM of *P. aeruginosa*, including neutralizing the surface negative charge by addition of positively changed L-Ara4N and altering the polarity and hydrophobicity by acylation and deacylation. The general mode of action of polymyxin involves the initial electrostatic interaction between the cationic side chains of the polymyxin molecule with the anionic lipid A head groups [83]. These events are subsequently followed by hydrophobic interactions between the *N*-terminal fatty acyl chain and position 6/7 hydrophobic side chains of the polymyxin with the hydrophobic fatty acyls of lipid A [83]. Therefore, in concept, both the addition of L-Ara4N and deacylation of lipid A should contribute to polymyxin resistance. Indeed, in our recent transcriptomic and neutron reflectometry studies, we discovered that deletion of the corresponding gene *pagL* (PA4661) resulted in an increased susceptibility to polymyxins in a polymyxin-resistant mutant PAK*pmrB6* derived from *P. aeruginosa* PAK [14, 88], demonstrating that the lipid A deacylation also plays a key role in the response of *P. aeruginosa* to polymyxin treatment.

Also, in our recent transcriptomics and metabolomics studies, we discovered that polymyxin treatment leads to remarkable growth reduction and metabolic perturbations in Gram-negative bacteria [41, 42, 89–91]. The integration of transcriptomics results into GSMMs allows for more accurate predictions of metabolic responses to either environmental (i.e., antibiotic treatment) or genetic perturbations (i.e., mutations) [92]. In the present study, we used the E-Flux method to integrate transcriptomics data as flux constraints [26]. E-Flux can map continuous gene expression levels to the metabolic network and uses the transcript abundance to determine the degree to which a reaction is active or inactive [26]. Therefore, E-Flux provides a more physiologically relevant description of the continuous nature of the reaction activity and avoids use of any artificial thresholds to binarize gene expression data [93]. In the present study, metabolic fluxes with and without antibiotic treatment were not calculated with minimization of metabolic adjustment (MOMA), as MOMA was developed to predict the metabolic flux redistributions in gene knockout mutants [94]. MOMA hypothesizes that metabolism of the mutant tends to approximate the wild type [94], which is distinct from the antibiotic treatment scenario. For instance, our metabolomics data have demonstrated that the antibiotic treatment caused dramatic metabolic changes in bacteria [41].

Comparison of the calculated flux distributions revealed that a broad range of metabolic perturbations occur in response to polymyxin treatment (Fig. 6), ranging from central carbon metabolism to oxidative phosphorylation and amino acid metabolism. Reduced growth, increased redox turnover, and decreased energy turnover due to polymyxin treatment were evident (Fig. 6), indicating that bacterial cells regulated their metabolism to produce more redox power to cope with the oxidative stress. This is consistent with previous findings that showed bactericidal antibiotics induced lethal oxidative damages by generating highly deleterious free radicals with subsequent culmination of cellular death [95]. In addition, our simulations revealed that polymyxin treatment induced an uptake of L-alanine, which was catabolized to generate more NADH (Fig. 6). This indicates that rich media (e.g., CAMHB) may provide abundant amino acids and peptides that can be utilized by bacterial cells to generate sufficient redox equivalents to cope with the oxidative damage caused by polymyxin treatment. Furthermore, our simulation results also showed an upregulated metabolic flux toward L-spermidine biosynthesis upon polymyxin B treatment (rxn00127 and rxn01406; Additional file 25). Previous studies showed that polyamines (e.g., spermidine) could protect *P. aeruginosa* from antimicrobial peptide killing [96]. It is assumed that the cationic spermidine could interact with the anionic LPS, mask the negative cell surface, and reduce the electrostatic interactions between polymyxin B and bacterial OM. Therefore, the enhanced biosynthesis of spermidine might increase its abundance at the cell surface and contribute to polymyxin resistance.

The constructed *i*PAO1 provides a detailed presentation of LPS biogenesis (Fig. 2), in particular, lipid A modifications. Further integration with specific regulatory modules will enable dynamic simulation of metabolic responses to polymyxin treatment. Previous studies revealed that various two-component regulatory systems (TCSs), including PhoPQ, PmrAB, ParRS, CprRS, and ColRS, play key roles in regulating polymyxin resistance [84, 97–100]. Among them, the PmrAB and PhoPQ systems are able to sense the depletion of external cations (e.g., Mg^2+^ and Ca^2+^) and upregulate the expression of the *arnBCADTEF-ugd* operon, which is responsible for the modification of lipid A with L-Ara4N [101]. Moreover, the fatty acylation of lipid A by PagP is under the control of PhoPQ [102, 103]. ParRS and CprRS are independent TCSs that mediate the upregulation of *pmrAB, arnBCADTEF-ugd* operon, *pagL*, and adaptive resistance in response to polymyxin treatment [97, 104]. In overview, lipid A modifications due to polymyxin treatment are strictly controlled by very complex regulatory networks that involve signal sensors, transcriptional regulators, and metabolic enzymes. Therefore, future studies are warranted to integrate these regulatory modules into the GSMM to enable simulating bacterial response dynamics to polymyxin treatment and analyzing adaptive resistance mechanisms in *P. aeruginosa*.

Overall, we have constructed, optimized, and validated a high-quality genome-scale metabolic model *i*PAO1 for *P. aeruginosa* PAO1. This comprehensive model incorporates metabolic pathways, particularly the biogenesis of membrane components, and enables delineation of the complex metabolic responses to antibiotics. *i*PAO1 provides a valuable systems tool for quantitative simulation of bacterial metabolic responses to antibiotics, elucidation of the molecular mechanisms of antimicrobial killing and resistance, and facilitation of designing rational antimicrobial combination therapy. To the best of our knowledge, this study is the first to integrate antimicrobial pharmacology, computational biology, a metabolic network, and systems pharmacology to analyze large-scale datasets in order to better understand the dynamic and complex nature of polymyxin killing and resistance. Combined with antibiotic pharmacokinetics and pharmacodynamics, *i*PAO1 offers an *in silico* platform for precision polymyxin chemotherapy.

## Conclusion

The generated collection of transcriptomics, metabolomics, lipidomics, and lipid A profiling data provides comprehensive datasets of *P. aeruginosa* for future integrative analysis of polymyxin systems pharmacology. As the largest curated GSMM thus far for *Pseudomonas, i*PAO1 represents all aspects of the cellular metabolism and may serve as the platform for integrative analysis of multiomics data. Simulation with transcriptomics constraints in this study revealed metabolic flux changes in amino acid catabolism, tricarboxylic acid cycle, and redox turnover caused by polymyxin treatment. Correlative analysis of metabolomics and transcriptomics with the constraint-based modeling is necessary for delineating the regulatory effects on metabolism. The methodology of using GSMMs to analyze multilevel omics data is applicable to other areas beyond antimicrobial pharmacology. Further integration with antimicrobial pharmacokinetics and pharmacodynamics will not only provide better pharmacological understanding but also empower the model to quantitatively predict the bacterial responses to antimicrobial therapy in the context of complex interplay of signaling, transcriptional regulation, and metabolism. In summary, our GSMM approach provides a powerful systems tool to elucidate the complex mode of action of antibiotics and will paradigm shift antimicrobial pharmacology.

## Availability of supporting data

The raw RNA-seq data have been deposited in the NCBI sequence read archive database under the BioProject accession number PRJNA414673. The metabolomics and lipidomics data have been deposited in the Metabolight database with accession number MTBLS630. Supporting data, including the scripts used in this project, are available via the *GigaScience* repository GigaDB [105].

## Additional files

Figure S1: Sensitivity analysis of the mean metabolic fluxes (A) and metabolite turnover rates to the variation of nutrient uptake upper bounds. Red indicates the control and blue indicates polymyxin B treatment.

Additional file 1 (additionalFile1.docx): Manual curation of GPL biosynthesis, LPS biosynthesis and modification pathways, and sensitivity analysis of nutrient uptake bounds.

Additional file 2 (additionalFile2.xlsx): Metabolites in the constructed draft model *i*PAO1_draft1.

Additional file 3 (additionalFile3.xlsx): Reactions in the constructed draft model *i*PAO1_draft1.

Additional file 4 (additionalFile4.xlsx): Genes in the constructed draft model *i*PAO1_draft1.

Additional file 5 (additionalFile5.xlsx): Supplemented metabolites according to previous GC-MS based metabolomics data.

Additional file 6 (additionalFile6.xlsx): Supplemented reactions according to previous GS-MS based metabolomics data.

Additional file 7 (additionalFile7.xlsx): Root gap metabolites identified using GapFind from the COBRA toolbox.

Additional file 8 (additionalFile8.xlsx): Comparison of the predicted growth phenotypes with the BIOLOG PM results.

Additional file 9 (additionalFile9.xlsx): Reactions with changed reversibility and directionality during manual curation.

Additional file 10 (additionalFile10.xlsx): Deleted metabolites during manual curation.

Additional file 11 (additionalFile11.xlsx): Deleted reactions during manual curation.

Additional file 12 (additionalFile12.xlsx): Added reactions during manual curation.

Additional file 13 (additionalFile13.xlsx): Added intermediate metabolites in GPL biosynthesis pathway.

Additional file 14 (additionalFile14.xlsx): Added reactions in GPL biosynthesis pathway.

Additional file 15 (additionalFile15.xlsx): Metabolites in the constructed model *i*PAO1.

Additional file 16 (additionalFile16.xlsx): Reactions in the constructed model *i*PAO1.

Additional file 17 (additionalFile17.xlsx): Genes in the constructed model *i*PAO1.

Additional file 18 (additionalFile18.xlsx): Biomass formation formula.

Additional file 19 (additionalFile19.xlsx): Comparison of the predicted gene essentiality with the information derived from two transposon insertion mutant libraries.

Additional file 20 (additionalFile20.xlsx): Metabolic flux changes in response to lipid A modifications using lipidomics data as stoichiometric constraints.

Additional file 21 (additionalFile21.xlsx): Randomized stoichiometric coefficients of LPS species.

Additional file 22 (additionalFile22.xlsx): Predicted physiochemical properties of lipid A molecules.

Additional file 23 (additionalFile23.xlsx): Metabolic flux changes in response to lipid A modifications with randomly assigned lipid A compositions as stoichiometric constraints.

Additional file 24 (additionalFile24.xlsx): Metabolic flux constraints calculated based on RNA-seq data.

Additional file 25 (additionalFile25.xlsx): Metabolic flux changes in response to polymyxin treatment using RNA-seq data as flux constraints.

Additional file 26 (additionalFile26.xlsx): Metabolite turnover rates.

Additional file 27 (additionalFile27.xlsx): Full names of the metabolite abbreviations in Figure 1.

## Abbreviations

TCS: two-component regulatory system; BLASTp: Basic Local Alignment Search Tool for Proteins; CAMHB: cation-adjusted Mueller-Hinton broth; COG: clusters of orthologous groups; FBA: flux balance analysis; FDR: false discovery rate; GC–MS: gas chromatography–mass spectrometry; GPL: glycerolphospholipid; GSMM: genome-scale metabolic models; IM: inner membrane; KEGG: Kyoto Encyclopaedia of Genes and Genomes; L-Ara4N: 4-amino-4-deoxy-L-arabinose; LB: Luria-Bertani; LPS: lipopolysaccharide; MDR: multidrug-resistant; MOMA: minimization of metabolic adjustment; NADH: reduced nicotinamide adenine dinucleotide; NCBI: National Center for Biotechnology Information; OM: outer membrane; PM: phenotypic microarray; RNA-seq: RNA-sequencing; RPKM: reads per kilobase million; TCA: tricarboxylic acid; TCDB: transporter classification database.

## Competing interests

The authors declare that they have no competing interest.

## Funding

This study was partially supported by a major interdisciplinary research grant from Monash University and a project grant by the Australian National Health and Medical Research Council (NHMRC; APP1127948). J.L., T.V., and J.S. are supported by the National Institute of Allergy and Infectious Diseases (NIAID) of the National Institutes of Health (NIH; R01 AI111965). The content is solely the responsibility of the authors and does not necessarily represent the official views of the NIAID or the NIH. T.V. is an Australian NHMRC Career Development Research Fellow. T.L. is an Australian Laureate Fellow supported by Australian Research Council. J.L. is an Australian NHMRC Senior Research Fellow.

## Author contributions

J.L. and F.S. conceived the project. Y.Z. developed the GSMM and conducted most analyses. T.C. and M.K. validated the model. J.Z., J.Lu, and B.S. curated the model. T.V., T.L., and J.S. helped supervise the project. M.H. and M.H.M.M. provided the lipidomics and transcriptomics data, respectively.

## Supplementary Material

GIGA-D-17-00272_Original_Submission.pdf

GIGA-D-17-00272_Revision_1.pdf

Response_to_Reviewer_Comments_Original_Submission.pdf

Reviewer_1_Report_(Original_Submission) -- Emanuele Bosi 28 Nov 2017 Reviewed

Reviewer_1_Report_(Revision_1) -- Emanuele Bosi 05 Feb 2018 Reviewed

Reviewer_2_Report_(Original_Submission) -- Jonathan Monk 12 Dec 2017 Reviewed

Supplemental material

## Notes

Part of this work was presented at the 27th European Congress of Clinical Microbiology and Infectious Diseases, 22–25 April 2017, Vienna, Austria.
