# Supplementary material for: Genome-scale metabolic modeling of responses to polymyxins in Pseudomonas aeruginosa
Source: Gigascience. 2018 Mar 13;7(4):giy021. doi: 10.1093/gigascience/giy021 (PMC6333913; doi:10.1093/gigascience/giy021)
Supplement: GIGA-D-17-00272_Revision_1.pdf [file giy021_giga-d-17-00272_revision_1.pdf]

GigaScience

Genome-scale metabolic modelling of responses to polymyxins in Pseudomonas aeruginosa

--Manuscript Draft--

**Manuscript Number:**	GIGA-D-17-00272R1
**Full Title:**	Genome-scale metabolic modelling of responses to polymyxins in Pseudomonas aeruginosa
**Article Type:**	Research
**Funding Information:**	Monash University (Major Interdisciplinary Research Grant) — Prof Jian Li<br>National Health and Medical Research Council (APP1127948) — Prof Jian Li<br>National Institute of Allergy and Infectious Diseases (R01 AI111965) — Prof Jian Li<br>National Health and Medical Research Council (APP1086825) — Dr Tony Velkov<br>National Health and Medical Research Council (APP1063069) — Prof Jian Li<br>Australian Research Council (FL130100038) — Prof Trevor Lithgow
**Abstract:**	Background: Pseudomonas aeruginosa often causes multidrug-resistant infections in immunocompromised patients and polymyxins are often used as the last-line therapy. Alarmingly, resistance to polymyxins has been increasingly reported worldwide recently. To rescue this last-resort class of antibiotics, it is necessary to systematically understand how P. aeruginosa alters its metabolism in response to polymyxin treatment, thereby facilitating the development of effective therapies. To this end, a genome-scale metabolic model (GSMM) was employed to analyse bacterial metabolic changes at the systems level.<br>Findings: A high-quality GSMM iPAO1 was constructed for P. aeruginosa PAO1 for antimicrobial pharmacological research. Model iPAO1 encompasses an additional periplasmic compartment and contains 3,022 metabolites, 4,265 reactions and 1,458 genes in total. Growth prediction on 190 carbon and 95 nitrogen sources achieved an accuracy of 89.1%, outperforming all reported P. aeruginosa models. Notably, prediction of the essential genes for growth achieved a high accuracy of 87.9%. Metabolic simulation showed that lipid A modifications associated with polymyxin resistance exert a limited impact on bacterial growth and metabolism, but remarkably change the physiochemical properties of the outer membrane. Modelling with transcriptomics constraints revealed a broad range of metabolic responses to polymyxin treatment, including reduced biomass synthesis, upregulated amino acids catabolism, induced flux through the tricarboxylic acid cycle, and increased redox turnover.<br>Conclusions: Overall, iPAO1 represents the most comprehensive GSMM constructed to date for Pseudomonas. It provides a powerful systems pharmacology platform for the elucidation of complex killing mechanisms of antibiotics.
**Corresponding Author:**	Jian Li<br><br>AUSTRALIA
**Corresponding Author Secondary Information:**	
**Corresponding Author's Institution:**	
**Corresponding Author's Secondary Institution:**	
**First Author:**	Yan Zhu

Powered by Editorial Manager® and ProduXion Manager® from Aries Systems Corporation

**First Author Secondary Information:**	
**Order of Authors:**	Yan Zhu
	Tobias Czauderna
	Jinxin Zhao
	Matthias Klapperstueck
	Mohd Hafidz Mahamad Maifiah
	Mei-Ling Han
	Jing Lu
	Björn Sommer
	Tony Velkov
	Trevor Lithgow
	Jiangning Song
	Falk Schreiber
	Jian Li
**Order of Authors Secondary Information:**	
**Response to Reviewers:**	Dr Laurie Goodman<br>Editor-in-Chief<br>GigaScience<br><br>Dear Dr Goodman,<br>RE: Manuscript ID: GIGA-D-17-00272: “Genome-scale metabolic modelling of responses to polymyxins in Pseudomonas aeruginosa"<br><br>Thank you for the opportunity to revise our manuscript. Please find below a point-by-point response to the reviewers’ comments. All major changes have been highlighted in yellow in the ‘marked’ version of the revised manuscript.<br><br>The raw data have been submitted to Sequence Read Archive (SRA) and MetaboLights databases and will be made publicly available hopefully by 31 Jan, 2018. We look forward to your correspondence and thank you very much.<br><br>Best regards,<br><br>Jian Li<br>Falk Schreiber<br><br>Monash Biomedicine Discovery Institute, Department of Microbiology, Faculty of Medicine, Nursing and Health Sciences, Monash University, Melbourne 3800, Australia<br><br>Department of Computer and Information Science, University of Konstanz, Konstanz 78457, Germany<br><br><br>Reviewer #1:<br><br>1. Introduction section. I would specify that the specific approach of applying gene expression constraints to obtain condition-specific GEMs have been previously used for other MDR bacteria (e.g. A. baumannii doi:10.1038/s41598-017-03416-2).<br><br>Response: The manuscript has been revised accordingly (Pages 3-4, Lines 74-77).<br><br>2. Lines 217-218: I would remove the sentence "Therefore, iPAO1 is a well-defined, metabolism-dedicated model.", in that it is included in the definition of "metabolic reconstruction". The presence of genes associated to non-metabolic COG categories is, in my opinion, due to the presence of misannotated (for what concerns the COG

Powered by Editorial Manager® and ProduXion Manager® from Aries Systems Corporation

categories) genes. Honestly, I wouldn't use the distribution of such categories as a measure of a model goodness, especially considering that some genes can be associated to multiple categories. All the other comparisons the authors made already highlighted how this reconstruction is the best one.

Response: The sentence was removed in the revised manuscript as suggested (Page 9, Line 221).

3. Lines 253-258: "this is possibly due to the incorporation of new genes (30.5% increase compared to Opt208964; 27.2% increase compared to iPae1146) whose metabolic functions were previously misannotated." This is not clear... do the authors mean that the addition of new genes brought alternative routes to bypass previously essential gene deletion? This should be rephrased, and, if possible, the proposed explanation should be tested.

Response: The sentence was rephrased as suggested. In the revised manuscript an example was provided to delineate that incorporating isozymes altered previous essentiality prediction results. Please refer Page 11, Lines 256-264 in the revised manuscript.

4. Section "Elucidating the mechanisms of metabolic responses to polymyxin treatment":
In this section the authors use the previously presented model to describe the changes at a systems level of the metabolism in presence of polymyxin treatment. I have two issues concerning this section: The way the authors computed the flux distribution in presence of antibiotics. Given the non-optimal state of such condition, I feel that MOMA is more appropriate. I suggest the authors to test this and compare the results with the current ones.

Response: We respectfully disagree with the reviewer. Minimisation Of Metabolic Adjustment (MOMA) was developed to predict the metabolic flux redistributions in gene knockout mutants. MOMA hypothesises that metabolism of the mutant tends to approximate the wild-type (Segre et al., 2002, Proc Natl Acad Sci. 99(23):15112-7), which is distinct from the antibiotic treatment scenario. For instance, our metabolomics data have demonstrated that polymyxin treatment caused dramatic metabolic changes in bacteria (e.g. Maifiah et al., 2017, Sci Rep, 7: 45527). Therefore, metabolic fluxes with and without antibiotic treatment should not be calculated with MOMA, but FBA (see e.g. Colijn et al., 2009, PLoS Comput Biol, 5(8): e10004). Please refer Pages 17, Lines 414-420 in our revised manuscript.

5. Although a description of the systemic changes induced by antibiotics is important, I think that the authors are missing an important point, that is the condition-specific essential genes. In my opinion this is very important and interesting, also considering that a selling point of the manuscript is that "iPAO1 offers an in silico platform for precision antimicrobial pharmacology therapy".

Response: We appreciate reviewer's suggestion. The methods and results on the condition-specific essential genes were included in the revised manuscript (Page 23, Lines 552-555; Page 11, Lines 264-269).

Reviewer #2:
1. On page 13 line 260 in the section on lipid A modification the authors mention changes in fluxes. They state that fluxes were calculated using FBA. However, in the Methods section I see that the authors used sampling to explore the solution space. The authors must use sampling to compare fluxes between conditions. If the author's used sampling here to the authors must specify so in the main text.

Response: We employed sampling in our original study and have specified the sampling methods in the revised manuscript as suggested. Please refer Page 12, Lines 274, 286-287.

2. Page 14 line 286 - the authors must state how the RNAseq was used to constrain the model. They mention it in the Discussion section (E-FLUX method). However, this must be stated in the Results section as well.

	Response: E-Flux method was employed to constrain the model with the RNAseq data, which has been specified in the Methods (Pages 24-25, Lines 586-592) and Results sections as suggested (Page 13, Line 302).<br><br>3. Page 14 line 295 - I have a major question about how the authors simulate for growth in CAMHB media? This is an undefined media type and in the Methods section they describe that they set the uptake rates to 1 mmol*gDW*hr^-1 for major carbon sources. The authors must explain why this uptake rate is justified. Did the authors perform any sensitivity analysis on these uptake rates? It's very reasonable to assume that changes in these rates would dramatically affect the fluxes described by the authors in this section. Some justification and or sensitivity analysis must be added here to explain the validity of these uptake rates for growth in this condition.<br><br>Response: Previous measurements showed that P. aeruginosa cells uptake amino acids at a rate ranging from 0.26 to 1.44 mmol·gDW^-1·h^-1 (J Bacteriol, 105(3): 1039-46; J Bacteriol, 152(2): 636-42). The import of CAMHB ingredients was thus constrained to 1 mmol·gDW^-1·h^-1 without loss of generality. Sensitivity analysis was conducted as suggested (Methods section, Page 25, Lines 592-597 and 600-602) and the results showed that the changes in nutrient uptake bounds did not dramatically affect the key metabolic fluxes. Our sensitivity analysis results have been provided in the Results section (Page 13, Lines 304-305), Additional File 1 and Figure S1.<br><br>4. Page 15 line 303 - the authors must state what the "control" condition is. Is this compared to PAO1 growing in CAMHB without polymyxin treatment? Or compared to growth on a different media type, i.e. M9 minimal media + glucose?<br><br>Response: The control condition was specified in the revised manuscript as suggested (Page 13 Line 321).<br><br>5. The authors state that their model is "the most comprehensive for a gram-negative organism to date". On what basis is this claim made? We would recommend tempering this statement or perhaps limiting it to Pseudomonas models.<br><br>Response: The statement was limited to Pseudomonas and was modified in the revised manuscript (Page 2, Line 46; Page 4, Line 98; Page 20, Line 472).
**Additional Information:**	
**Question**	**Response**
Are you submitting this manuscript to a special series or article collection?	No
**Experimental design and statistics**<br><br>Full details of the experimental design and statistical methods used should be given in the Methods section, as detailed in our Minimum Standards Reporting Checklist. Information essential to interpreting the data presented should be made available in the figure legends.<br><br>Have you included all the information requested in your manuscript?	Yes
**Resources**<br><br>A description of all resources used,	Yes

Powered by Editorial Manager® and ProduXion Manager® from Aries Systems Corporation

including antibodies, cell lines, animals and software tools, with enough information to allow them to be uniquely identified, should be included in the Methods section. Authors are strongly encouraged to cite Research Resource Identifiers (RRIDs) for antibodies, model organisms and tools, where possible.<br><br>Have you included the information requested as detailed in our Minimum Standards Reporting Checklist?	
**Availability of data and materials**<br><br>All datasets and code on which the conclusions of the paper rely must be either included in your submission or deposited in publicly available repositories (where available and ethically appropriate), referencing such data using a unique identifier in the references and in the “Availability of Data and Materials” section of your manuscript.<br><br>Have you have met the above requirement as detailed in our Minimum Standards Reporting Checklist?	Yes

Powered by Editorial Manager® and ProduXion Manager® from Aries Systems Corporation

Manuscript

Click here to download Manuscript MetabolicModeling22-2.docx

Genome-scale metabolic modelling of responses to polymyxins in *Pseudomonas aeruginosa*

Yan Zhu[1], Tobias Czauderna[2], Jinxin Zhao[1], Matthias Klapperstueck[2], Mohd Hafidz Mahamad Maifiah[3], Mei-Ling Han[3], Jing Lu[4], Björn Sommer[5], Tony Velkov[6], Trevor Lithgow[1], Jiangning Song[1], Falk Schreiber[2,5*], Jian Li[1*]

[1]Monash Biomedicine Discovery Institute, Department of Microbiology, Faculty of Medicine, Nursing and Health Sciences, Monash University, Melbourne 3800, Australia; [2]Faculty of Information Technology, Monash University, Melbourne 3800, Australia; [3]Faculty of Pharmacy and Pharmaceutical Sciences, Monash University, Melbourne 3052, Australia; [4]Monash Institute of Cognitive and Clinical Neurosciences, Department of Anatomy and development biology, Faculty of Medicine, Nursing and Health Sciences, Monash University, Melbourne 3800, Australia; [5]Department of Computer and Information Science, University of Konstanz, Konstanz 78457, Germany; [6]Department of Pharmacology and Therapeutics, University of Melbourne, Melbourne 3010, Australia.

Email addresses: YZ, yan.zhu@monash.edu; TC, tobias.czauderna@monash.edu; JZ, jinxin.zhao@monash.edu; MK, matthias.klapperstueck@monash.edu; MHMM, hafidz.maifiah@monash.edu; MLH, meiling.han@monash.edu; JLU, jing.lu2@monash.edu; BS, bjoern.sommer@uni-konstanz.de; TV, tony.velkov@unimelb.edu.au; TL, trevor.lithgow@monash.edu; JS, jiangning.song@monash.edu; FS, falk.schreiber@uni-konstanz.de; JL, jian.li@monash.edu.

Running title: Metabolic modelling polymyxins responses

*Corresponding authors:

Falk Schreiber, Email: falk.schreiber@uni-konstanz.de

Jian Li, 19 Innovation Walk, Monash University, VIC 3800, Australia. Tel: +61 3 9903 9702, Fax: +61 3 9902 9222, Email: jian.li@monash.edu.

Part of this work was presented at the 27th European Congress of Clinical Microbiology and Infectious Diseases, 22-25 April 2017, Vienna, Austria.

Abstract

Background: *Pseudomonas aeruginosa* often causes multidrug-resistant infections in immunocompromised patients and polymyxins are often used as the last-line therapy. Alarmingly, resistance to polymyxins has been increasingly reported worldwide recently. To rescue this last-resort class of antibiotics, it is necessary to systematically understand how *P. aeruginosa* alters its metabolism in response to polymyxin treatment, thereby facilitating the development of effective therapies. To this end, a genome-scale metabolic model (GSMM) was employed to analyse bacterial metabolic changes at the systems level.

Findings: A high-quality GSMM *i*PAO1 was constructed for *P. aeruginosa* PAO1 for antimicrobial pharmacological research. Model *i*PAO1 encompasses an additional periplasmic compartment and contains 3,022 metabolites, 4,265 reactions and 1,458 genes in total. Growth prediction on 190 carbon and 95 nitrogen sources achieved an accuracy of 89.1%, outperforming all reported *P. aeruginosa* models. Notably, prediction of the essential genes for growth achieved a high accuracy of 87.9%. Metabolic simulation showed that lipid A modifications associated with polymyxin resistance exert a limited impact on bacterial growth and metabolism, but remarkably change the physiochemical properties of the outer membrane. Modelling with transcriptomics constraints revealed a broad range of metabolic responses to polymyxin treatment, including reduced biomass synthesis, upregulated amino acids catabolism, induced flux through the tricarboxylic acid cycle, and increased redox turnover.

Conclusions: Overall, *i*PAO1 represents the most comprehensive GSMM constructed to date for *Pseudomonas*. It provides a powerful systems pharmacology platform for the elucidation of complex killing mechanisms of antibiotics.

Keywords: Genome-scale metabolic model; *Pseudomonas aeruginosa*; polymyxin; lipid A modification; outer membrane

Background

Pseudomonas aeruginosa is a common multidrug-resistant (MDR) pathogen in immune-compromised patients, cystic fibrosis patients and burns victims [1-6]. It possesses a large genome (5.5-7.0 Mb), complex regulatory networks, remarkable metabolic versatility and an extraordinary ability to survive extremely harsh conditions such as prolonged antibiotic exposure [7, 8]. Polymyxins (i.e. polymyxin B and colistin) have been increasingly used as a last-line therapy to treat infections caused by MDR *P. aeruginosa* [9]. Alarmingly, the prevalence of polymyxin resistance in *P. aeruginosa* has increased worldwide over the past few years [3, 10, 11].

The exact mode of action of polymyxins is not clear except the initial electrostatic and hydrophobic interactions with lipid A, a component of the lipopolysaccharide (LPS) in the bacterial outer membrane (OM). Subsequently, cell envelope is disorganised, cellular contents leak, oxidative stress increases, and finally cell death occurs [2, 9, 12, 13]. After polymyxin treatment, *P. aeruginosa* modifies its lipid A structure to attenuate the aforementioned electrostatic interactions [14]. Our recent metabolomics data demonstrated that, apart from lipid A modifications, numerous biochemical pathways are perturbed by polymyxin treatment, indicating that the development of polymyxin resistance by *P. aeruginosa* involves a complicated interplay of multiple cellular processes [15]. There are significant gaps in the knowledge-base of the mechanisms of polymyxin activity and bacterial responses in *P. aeruginosa*, thereby necessitating comprehensive investigations using systems pharmacology approaches.

With the rapid development of genome-scale metabolic models (GSMMs) and the associated

flux balance analysis (FBA) methods, systematic investigations into the metabolic changes in response to external nutrient alterations, genetic perturbations, and antibiotic treatments become feasible [16-24]. Several studies employed transcriptomics data as constraints to compute condition-specific metabolic flux changes in response to antibiotic treatments in MDR bacteria, including *Acinetobacter baumannii* [25], *Mycobacterium tuberculosis* [26] and *Yersinia pestis* [27]. For *P. aeruginosa*, four GSMMs have been constructed, iMO1056 [28], Opt208964 [29], iMO1086 [30] and the latest iPae1146 [31]. iMO1056, Opt208964 and iPae1146 employed SEED metabolite and reaction names; iMO1056 and Opt208964 are fully accessible via Model SEED [29, 31, 32]; iMO1086 employed different identifiers (IR/RR plus five digits for reactions and C/EC plus four digits for metabolites) [30]. The previous applications of these models have included simulating the metabolic dynamics in cystic fibrosis patients [33], elucidating the mechanisms of biofilm formation [34, 35], predicting potential drug targets [36-38] and identifying the key genes controlling virulence factors [31]. As important as they have been, these models have several overarching limitations. Those past models (i) do not include a major cellular component, the periplasmic space; (ii) have poor representation of glycerophospholipid (GPL) biosynthesis; and (iii) lack lipid A modification reactions. Considering the pathogenesis of *P. aeruginosa*, these major limitations significantly compromise the modelling functions. In particularly, the power of the four reported GSMMs to predict metabolic responses to antibiotic treatment is very limited, as periplasmic GPL and LPS biogenesis play critical roles in responses to anti-pseudomonal antibiotics such as polymyxins [15, 39-42].

Here we describe *i*PAO1, a newly developed GSMM for *P. aeruginosa* PAO1 based upon Opt208964 [29] and iMO1056 [28] but with intensive manual curation using several major databases and the literature. Most notably, *i*PAO1 is the first GSMM for *P. aeruginosa* where the periplasmic space compartment is incorporated to comprehensively represent cross-

membrane transport, GPL metabolism and LPS biosynthesis. To the best of our knowledge *i*PAO1 represents the most comprehensive metabolic reconstruction for *Pseudomonas* thus far. Modelling with *i*PAO1 revealed that the lipid A modifications might exert limited impact on cell growth and metabolism but change the physiochemical properties of bacterial OM. Constrained by gene expression levels, the model was employed to elucidate the metabolic responses to polymyxin B treatment. Together, *i*PAO1 provides a powerful systems platform for antimicrobial pharmacological research to combat the rapidly increasing resistance.

Data Description

The genome sequence and annotation of *P. aeruginosa* PAO1 were obtained from GenBank (Accession NC_002516.2). Models iMO1056 and Opt208964 were retrieved from Model SEED [32]. The gas chromatography–mass spectrometry (GC-MS) metabolomics data were collected from the literature [43]. Metabolites, reactions and pathways were obtained from databases KEGG (Kyoto Encyclopaedia of Genes and Genomes) [44], MetaCyc [45], TCBD (Transporter Classification Database) [46], TransporterDB [47] and Pseudomonas Genome DB [48]. Growth phenotypes on 190 carbon sources and 95 nitrogen sources were determined using BIOLOG Phenotypic Microarrays. Non-essential gene lists were collected from two previously reported transposon mutant libraries for PAO1 [49, 50]. Lipid A of wild-type *P. aeruginosa* PAK and its polymyxin-resistant mutant PAK*pmrB6* was extracted using mild acid hydrolysis method and the structural analysis of lipid A was conducted using mass spectrometry [42]. RNA was extracted and employed to construct cDNA libraries for RNA-Seq on Illumina MiSeq platform [51]. The raw reads were quality trimmed and aligned to PAO1 reference genome using SubRead [52]. Counts were normalised and the differential gene expression was determined using voom/limma packages with Degust [53]. Whole-cell lipids and intracellular metabolites were extracted using the single-phase Bligh-Dyer method as previously described

and analysed by liquid chromatography-mass spectrometry (LC-MS) [14, 42]. Raw metabolomics data were processed with IDEOM software followed by bioinformatics analysis [54].

Analyses

Development of a superior GSMM for *P. aeruginosa* PAO1

Initially, a draft model (*i*PAO1_draft1) containing 1,991 reactions, 1,579 metabolites and 1,021 genes was created based upon iMO1056 [28] and Opt208964 [29] (**Additional files 2-4**). To obtain a high-quality GSMM, extensive manual curation was conducted. Firstly, *i*PAO1_draft1 was complemented using databases and the literature. Specifically, the following additional information was incorporated into the draft model, 285 metabolites and 36 reactions from KEGG [44], 225 metabolites and 50 reactions from MetaCyc [45], and 7 metabolites and 20 reactions obtained by previous GC-MS-based quantification [43] (**Additional files 5 and 6**).

Secondly, a periplasmic compartment was built to incorporate 698 periplasmic metabolites, 509 transport reactions across the inner membrane (IM), 441 transport reactions across the outer membrane (OM), and 403 periplasmic reactions. The resulting intermediate model was designated as *i*PAO1_draft2.

Thirdly, the major pathway gaps were filled. GapFind [55] identified 109 dead-end metabolites (**Additional file 7**). The growth phenotypes on 190 carbon and 95 nitrogen nutrients were predicted using *i*PAO1_draft2, and compared with our experimental BIOLOG Phenotypic Microarray (PM) results (**Additional file 8**). As a result, 162 false negative predictions (i.e. the prediction indicated non-growth whereas the BIOLOG experiment demonstrated valid growth on a specific nutrient) were determined, indicating the lack of associated transport or catabolic reactions for these nutrients. To link the dead-end metabolites back to the metabolic network

and eliminate inconsistencies with the BIOLOG PM results, several modifications were made including (i) adjustment of the reversibility settings of 180 reactions and changing the directions of 87 reactions (**Additional file 9**); (ii) removal of 14 metabolites and 96 reactions (**Additional files 10 and 11**), which were either duplicated (e.g. β-D-glucose was duplicated with D-glucose) or representing general metabolite classes (e.g. protein, mRNA, DNA); and (iii) addition of 98 boundary reactions, 677 transport reactions, and 252 metabolic reactions (**Additional file 12**). Resolving the false negative predictions of the BIOLOG growth phenotypes substantially improved the model. For example, predictions using *i*PAO1_draft2 showed that PAO1 was unable to grow with formic acid as a sole carbon source due to the lack of the corresponding transport reaction. Interrogating the Pseudomonas Genome Database [48] and Pfam [56] identified PA2777, a hypothetical protein in NCBI and UniProt which may encode formic/nitrite transporter (Pfam01226, *P*=7e-34). Subsequent addition of the transport reaction (rxn08526) enabled *in silico* growth of PAO1 on formic acid. Another example is that initially *i*PAO1_draft2 failed to predict utilisation of 1,2-propanediol for growth owing to the exiting gap in dehydrogenation of 1,2-propanediol to lactaldehyde. Using BLASTp with the query sequence of lactaldehyde reductase (*fucO*, *b2799*) from *Escherichia coli* K12 MG1655 identified a candidate homologous gene PA1991 (Identity=35%, Eval=2e-75, BLASTp). PA1991 encodes an iron-containing alcohol dehydrogenase and has over 300 orthologues in Gram-negative bacteria which encode lactaldehyde oxidoreductases or 1,2-propanediol dehydrogenases according to OrthoDB [57]. Inactivation of PA1991 resulted in 8-fold prolonged lag phase when *P. aeruginosa* grew on 1,2-propanediol [58]. Therefore, reaction rxn01615 oxidising 1,2-propanediol to lactaldehyde was added into *i*PAO1_draft2. A very large number of such labour intensive manual curations were conducted to improve the model. This enabled *in silico* growth on a number of nutrients from BIOLOG PM, including 4-hydroxyphenylacetate, tyramine, quinic acid, itaconic acid, citramalic acid, L-pyroglutamic

acid, carnidine, glycinebetaine, L-methylsuccinate, and D-amino acids (**Additional file 8**).

Fourthly, the biogenesis of bacterial envelope was delineated. Cross-linking between amino acids residues among peptidoglycan chains results in a rigid network structure in *P. aeruginosa* [59]. In total, 17 reactions representing peptidoglycan cross-linking and hydrolysis were incorporated by searching for homologues of glycosyltransferases, transpeptidases, carboxypeptidases and endopeptidases in PAO1 [60]. Overall, a detailed peptidoglycan biosynthesis pathway was constructed with 60 reactions. GPL compositions in the bacterial membranes can change in response to antibiotic treatment [39, 61]. Previous studies [62] and our own lipidomics results [14] showed a great diversity in GPL species in *P. aeruginosa*. Overall, 386 unique metabolites (i.e. 66.2% of the 583 metabolites in the GPL metabolism pathway) and 367 reactions (66.7% of the 550 reactions in the GPL metabolism pathway) were incorporated into *i*PAO1_draft2 (**Additional files 1, 13 and 14, Fig 1**). LPS consists of lipid A, core oligosaccharide, and *O*-antigen polysaccharide [40], and plays key roles in the host-pathogen interaction and the resistance to antibiotics such as polymyxins [13, 63]. A detailed synthesis and interconversion network was generated with 432 types of LPS and 1,169 reactions (**Fig 2, Additional file 1**). Notably, our GSMM is the most comprehensive to date in lipid A biosynthesis and modifications.

The resulting final *i*PAO1 model consisted of 3,022 metabolites, 4,365 reactions and 1,458 genes (25.8% of the PAO1 genome, **Additional files 15-17**), representing, respectively, (i) 252%, 340% and 40% increase of the components in iMO1056; and (ii) 125%, 171% and 43% increase of the components in Opt208964 (**Table 1**). The significant expansion in *i*PAO1 includes cross-membrane transport, GPL/LPS biosynthesis, peptidoglycan biosynthesis, and fatty acid degradation (**Additional files 15-17**). The reactions from *i*PAO1 were categorised into 109 pathways mainly based on classifications in MetaCyc and KEGG. In *i*PAO1, 27.9%/43.7%/51.6% metabolites, 20.3%/33.5%/59.5% reactions and 65.3%/17.6%/28.5%

genes are originated from iMO1056, Opt208964, and our manual curation, respectively (**Fig 3A**).

Components in *i*PAO1 were aligned with databases including KEGG [44], MetaCyc [45], PubChem [64], ChemSpider [65], ChEBI [66], Model SEED [32], and BiGG [67] (**Additional files 15 and 16**). Consequently, 1,404 (46.5%), 1,590 (52.6%) and 2,142 (70.9%) metabolites have corresponding identifiers in MetaCyc, KEGG and Model SEED, respectively; 1,556 (35.6%), 1,596 (36.6%) and 1,964 (45.0%) reactions were computationally mapped to the reactions from MetaCyc, KEGG and Model SEED, accordingly (**Fig 3B**). A significant portion of mismatches were caused by the incorporation of specific types of metabolites in the GPL metabolism and LPS biosynthesis pathway, which in databases are usually lumped as general compound classes. The properties of metabolites, including mass, charge and formula were included in *i*PAO1. The standard Gibbs free energy change of formation ($\Delta_f G^o$), and reaction ($\Delta_r G^o$) were obtained from MetaCyc and Model SEED for 1,877 metabolites (62.1%) and 1,355 reactions (31.0%) (**Additional files 15 and 16**).

A breakdown of genes involved in *i*PAO1 (**Additional file 17**) using the clusters of orthologous groups (COGs) showed remarkable improvement compared to previous reconstructions (**Fig 3C**). The largest increase in the coverage compared to iMO1056 is lipid transport and metabolism (24.1%), followed by inorganic ion transport and metabolism (19.3%); whereas compared to Opt208964, the largest increase in the coverage is nucleotide transport and metabolism (57.9%), followed by amino acid transport and metabolism (52.0%). Overall, the transport and metabolism of nucleotides and amino acids showed the highest percent coverage of COG functional categories in *i*PAO1 (72.9% and 65.6%, respectively). Notably, the reactions in categories not apparently related to metabolism were dramatically reduced in *i*PAO1 compared to Opt208964, including translation, ribosomal structure and biogenesis, posttranslational modification, protein turnover, chaperones and signal transduction

mechanisms, or undetermined categories including function unknown class.

In *i*PAO1, GPL metabolism, LPS biosynthesis and transport across OM were ranked the three largest pathways and also contained the highest proportion of curated reactions (**Fig 3D**). Additionally, these three pathways have high reaction-to-gene ratios (13.1-24.2, **Fig 3E**), indicating that enzymes in these pathways are capable of acting on a broad range of substrates. As kinetic parameters are usually not involved in a GSMM, constraint-based analyses (e.g. FBA) of a GSMM do not directly account for enzyme levels, intracellular metabolic concentrations or substrate-level regulation. Accordingly, the affinity difference of various substrates was not considered in our *i*PAO1 modelling effort.

We employed the biomass formation equation from iMO1056 to construct *i*PAO1 with modifications on LPS and ion species (**Additional file 18**). In addition, to take into account the extra energy consumption caused by charging tRNAs, the original amino acids in the biomass formation reaction were replaced by aminoacyl-tRNA, followed by addition of specific charging reactions to the model. Taken together, *i*PAO1 represents the most comprehensive metabolic reconstruction thus far for *P. aeruginosa* PAO1.

Growth capability on various nutrients

Investigation of nutrient utilisation using BIOLOG PM showed PAO1 could utilise a broad range of nutrient sources, indicated by the observed growth on 68 of 190 (35.8%) carbon and 76 of 95 (80.0%) nitrogen substrates (**Fig 4**). Growth simulation with *i*PAO1 achieved an overall accuracy of 89.1% (254 of 285), which substantially outperformed previous models (81.5% for Opt208964 [29], 77.9% for iMO1056 and iMO1086 [30] and 80% for iPae1146 [31]. Twenty-one false-positive and 10 false-negative (**Fig 4**, **Additional file 8**) disagreements were observed, possibly due to the complexity of regulatory mechanisms and missing annotation of nutrient transport and/or catabolism pathways in PAO1.

Prediction and validation of gene essentiality

In silico single-gene deletion with *i*PAO1 showed 143 essential genes (μ_{mut}<0.01 μ_{wt}), 40 semi-essential genes (0.01 μ_{wt}<μ_{mut}<0.99 μ_{wt}), and 1,275 non-essential genes (0.99 μ_{wt}<μ_{mut}< μ_{wt}) when growing in Luria-Bertani (LB) media (**Additional file 19**). Among the essential metabolic genes, the largest COG proportion (46 of 143, 32.1%) is cell envelope biogenesis, indicating that there are relatively less alternative reactions in this pathway. For non-essential genes, amino acid transport and metabolism (352 of 1,315, i.e. 26.7%) represents the largest group, suggesting the existence of large metabolic redundancy.

The predicted gene essentiality was further verified by two independent genome-scale transposon mutant libraries [49, 50, 68]. The overall prediction accuracy achieved 87.9%, which is higher than iMO1056 (85.0%) [28] and iMO1086 (84.2%) [30], but slightly lower than Opt208964 (92.9%) [29] and iPae1146 (91.46%) [31]. The higher accuracy in Opt208964 is partially due to errors in the annotation of essential genes. For instance, 351 genes in Opt208964 were grouped as experimentally validated essential; however, 145 out of the 351 genes are non-essential as their corresponding mutants were found in the transposon mutant library [50]. In iPae1146, removal of 16 isozymes increased the prediction accuracy of essential genes; for example, 3-ketoacyl-ACP reductase (EC 1.1.1.100) reactions in iPae1146 were associated with PA2967 only [31], whereas in *i*PAO1, these reactions were associated with another eight highly conserved isozymes (PA0182, PA1470, PA1827, PA3387, PA4089, PA4389, PA4786, PA5524). Furthermore, condition-specific essential genes were predicted in *i*PAO1 by imposing transcriptomics constraints. Modification of lipid A with 4-amino-4-deoxy-L-arabinose (L-Ara4N) leads to polymyxin resistance in *P. aeruginosa* and deficiency in *arn* genes reverses the susceptibility [69]. Seven additional essential genes (*arnABCDEFT*, PA3552-3558, encoding L-Ara4N biosynthesis) were predicted by *i*PAO1 under polymyxin treatment.

Impact of lipid A modifications on bacterial growth and metabolism

P. aeruginosa modifies lipid A components in the OM in response to polymyxin treatment [70]. The LPS stoichiometric coefficients in the biomass formula of *i*PAO1 were configured based on our lipidomics data (**Table 2**, [14]), and the metabolic impact of lipid A modifications was predicted by randomly sampling the metabolic solution space with 10,000 points (*cf.* Methods). Overall, 273 fluxes were significantly affected (*Z*-Score, false discovery rate (FDR) <0.01; >0.1 mmol·gDW^{-1}·h^{-1} under at least one condition, **Additional file 20**). The specific growth rate remained unchanged. A 0.026 mmol·gDW^{-1}·h^{-1} flux from glucose via glucose 6-phosphate, uridine diphosphate glucose, and consequently L-Ara4N biosynthesis was identified due to lipid A modifications. The overall fluxes through lipid A deacylation reactions were increased (from 0.007 mmol·gDW^{-1}·h^{-1} to 0.011 mmol·gDW^{-1}·h^{-1}); the generated (*R*)-3-hydroxydecanoate was fuelled into β-oxidation to produce octanoyl-CoA, which was subsequently salvaged for fatty acid biosynthesis.

To further investigate the impact of lipid A modifications on bacterial growth, 1,000 sets of the compositions of 288 heterogeneous LPS molecules were randomly generated with the total proportion of LPS unchanged in the biomass formation formula (**Additional file 21**). The metabolic fluxes were calculated for each of the 1,000 sets of LPS compositions by randomly sampling the solution space with 10,000 points. Across the 1,000 sets of metabolic fluxes (**Additional file 23**), the specific growth rate varied between 0.8812 and 0.8897 mmol·gDW^{-1}·h^{-1}. Correlative analysis of the apparent overall physiochemical properties of lipid A (**Additional file 22**) with the predicted growth phenotypes showed three interesting findings. Firstly, addition of L-Ara4N reduced the negative charge of lipid A (ρ=1.00), decreased the hydrophobicity of the OM (represented by logP, ρ=-0.59), but required assimilation of more ammonia (represented by ammonia turnover, ρ=0.57). Secondly, hydroxylation on acyl chains

of lipid A exerted minor effects over either bacterial growth or physiochemical properties. Thirdly, addition of acyl chains resulted in large lipid A molecules (represented by the atomic counts, ρ=0.88), enhanced molecular polarity of lipid A (ρ=0.87), increased OM hydrophobicity (ρ=0.75), and notably, retarded growth (ρ=-0.95), reduced redox and energy turnover (ρ=-0.98 for both), and increased requirement of ammonia (ρ=0.59) (**Fig 5**). It is evident that none of the three aforementioned modifications produced a dramatic impact on bacterial growth or metabolism (**Additional file 23**).

Elucidating the mechanisms of metabolic responses to polymyxin treatment

RNA-Seq data were utilised as model constraints (**Additional file 24**) with an E-Flux method [71] to calculate the metabolic fluxes in the absence and presence of polymyxin B (*cf*. Methods). The exchange fluxes were constrained based on the maximum uptake rates of the media ingredients (*cf*. Methods and **Additional file 1**). Comparison of the flux distributions revealed that 1,392 reactions were differentially regulated (FDR<0.01, **Additional file 25**). A range of metabolic pathways were significantly disturbed, including central metabolism, amino acid metabolism, purine biosynthesis, fatty acid biosynthesis and metabolism, LPS and GPL biosynthesis and transport reactions. Polymyxin B treatment reduced the growth rate (18.2%), increased oxygen uptake (6.9%) and CO_2 emission (6.0%); however, the respiration quotient remained roughly unchanged (**Table 3**).

As the major carbon sources, the amino acids and oligopeptides from cation-adjusted Mueller-Hinton broth (CAMHB) were utilised to generate intermediate metabolites, redox and energy equivalents for biomass formation. In response to polymyxin treatment, the gluconeogenesis pathway was significantly induced from pyruvate to 3-phosphoglycerate, but suppressed from 3-phosphoglycerate towards glucose 6-phosphate. The extra flux from 3-phosphoglycerate was shunt to serine and glycine biosynthesis (**Fig 6**) via 3-phospho-D-glycerate:NAD^+

oxidoreductase (rxn01101), 3-phosphoserine:2-oxoglutarate aminotransferase (rxn02914), *O*-phospho-L-serine phosphohydrolase (rxn00420), and 5,10-methylenetetrahydrofolate:glycine hydroxymethyltransferase (rxn00692), through which more NADH equivalent was generated compared to the control (i.e. growth in CAMHB without polymyxin treatment). The resulting one-carbon unit in 5,10-methylenetetrahydrofolate was oxidised to formic acid via 10-formyltetrahydrofolate amidohydrolase (rxn00691); the generated glycine was fuelled into TCA cycle via glycine:oxygen oxidoreductase (rxn00269) and acetyl-CoA:glyoxylate C-acetyltransferase (rxn00330). In addition, the metabolic flux via TCA cycle was upregulated from citrate to fumarate, with increased NADH production. Within oxidative phosphorylation, the mean fluxes through NADH dehydrogenase (Complex I, rxn10122), cytochrome bc1 complex (Complex III, rxn13820), and cytochrome c oxidase (Complex IV, rxn13688) decreased by 6.6%, 7.2% and 7.8%, respectively. The flux via F_0F_1-ATPase (Complex V, rxn10042) was downregulated by 11.1%. The overall fluxes via biosynthesis of macromolecules including LPS, GPL and peptidoglycan decreased due to the significantly reduced biomass formation. The biosynthesis of spermidine increased by 38.3% in response to polymyxin treatment which was also indicated by upregulated expression of *speD* (PA4773; encoding the *S*-adenosyl-L-methionine decarboxylase, $\log_2$FC=3.62, FDR<0.01) and *speE* (PA4774; encoding spermidine synthase, $\log_2$FC = 3.54, FDR<0.01).

Calculating the flux-sum of critical cofactors revealed 13.1% increase of redox turnover and 8.2% decline of energy turnover after 1 mg·L^{-1} polymyxin B treatment for 1 h. Breaking down the cofactors showed the turnover of major redox equivalents NADH, NADPH, ubiquinol-8 and FADH2 substantially increased by 12.6%, 13.9%, 3.9% and 35.9%, respectively; whereas the turnover of ATP, the major contributor to energy significantly decreased by 8.52% after 1 mg·L^{-1} polymyxin treatment for 1 h (**Fig 6**, **Additional file 26**). Overall, metabolic flux analysis using *i*PAO1 integrated with our transcriptomics data revealed a significant global impact on

bacterial metabolism due to polymyxin B treatment.

Discussion

The emergence of Gram-negative 'superbugs' that are resistant to the last-resort polymyxins highlights the urgent need for novel approaches such as GSMMs to understand the mechanisms of antibacterial activity and resistance. The main utility of GSMMs is their ability to bridge critical gaps between genomics and metabolic phenotypes through the prediction of metabolic responses to antimicrobial treatments at the network level. Here, we report the development, optimisation, validation and application of a high-quality GSMM designated *i*PAO1 for a type strain *P. aeruginosa* PAO1; and importantly, *i*PAO1 was employed to understand the complicated effect of polymyxin treatment on bacterial metabolism. Simulation with *i*PAO1 showed that lipid A modifications in response to polymyxin treatment only exert minor effects on bacterial growth and metabolism. Albeit, further calculations that integrate transcriptomics data as model constraints revealed that polymyxin treatment may reduce growth and affect a broad range of pathways.

*i*PAO1 represents the most comprehensive metabolic model for *P. aeruginosa* to date and incorporates 1,458 genes, accounting for ~25.8% of the PAO1 genome. Among the four GSMMs developed for *P. aeruginosa* PAO1, iMO1086 and iPae1146 were constructed on the basis of iMO1056 with moderate increase of metabolites, reactions and genes [28, 30, 31]; Opt208964 is also in a medium size, which limits modelling capacity [29]. In contrast, *i*PAO1 is significantly expanded in model scale, by doubling or even tripling the numbers of metabolites and reactions (**Fig 3A**). *i*PAO1 achieved an unprecedented prediction accuracy of 89.1% for growth on various nutrients, outperforming all of the previously reported GSMMs for *P. aeruginosa* [28-31]. The *i*PAO1 model was also employed to predict gene essentiality

with a high accuracy of 87.9%. Given the extensive curation and significant expansion, *i*PAO1 will serve as the primary reference for future development of metabolic models, particularly for other *P. aeruginosa* strains.

Unlike *i*PAO1, none of the previous *P. aeruginosa* GSMMs incorporated the periplasm. As polymyxins initially target LPS in the OM and can cause substantial changes in the cell envelope, the periplasmic space is a major component in *i*PAO1. The periplasmic space of *E. coli* is estimated to constitute up to 16% of total cell volume [72]. It contains a thin cell wall composed of peptidoglycan and a variety of ions and proteins, which are involved in transport, folding, cell envelope biogenesis, electron transport and xenobiotic metabolism [73]. *i*PAO1 is the first *P. aeruginosa* GSMM to incorporate the periplasmic compartment, enabling accurate representation of metabolic machinery, especially for those reactions that occur exclusively in this important cellular space and transport of substrates across the IM and OM. Furthermore, *i*PAO1 provides detailed representations of GPL and LPS biosynthesis which allows the precise mapping of GPL and LPS responses from experimental metabolomics and lipidomics data (**Figs 1 and 2**).

In response to polymyxin treatment, Gram-negative bacteria modify their lipid A with cationic moieties (i.e. phosphoethanolamine and L-Ara4N) that act to repel the like-charge of the polymyxin molecule [40]. Based on our simulations (**Additional file 20**), we purport that such lipid A modifications exerted a limited impact on cellular metabolism and growth. Most of the flux changes were insignificant; the remaining significant flux changes mainly resulted from futile cycles containing sets of reactions using redox equivalents, whereas the net carbon flow remained unchanged. Simulation using randomised lipid A compositions further consolidated our hypothesis that lipid A modifications cause only moderate variations of bacterial growth and metabolism (**Fig 5, Additional file 23**). Notwithstanding, our simulation results revealed that lipid A modifications result in substantial physiochemical changes in the OM of *P.*

aeruginosa, including (i) neutralising the surface negative charge by addition of positively changed L-Ara4N; and (ii) altering the polarity and hydrophobicity by acylation and deacylation. The general mode of action of polymyxin involves the initial electrostatic interaction between the cationic side chains of the polymyxin molecule with the anionic lipid A head groups [63]. These events are subsequently followed by hydrophobic interactions between the *N*-terminal fatty acyl chain and position 6/7 hydrophobic side chains of the polymyxin with the hydrophobic fatty acyls of lipid A [63]. Therefore, in concept both the addition of L-Ara4N and deacylation of lipid A should contribute to polymyxin resistance. Indeed, our recent transcriptomic and neutron reflectometry studies discovered that deletion of the corresponding gene *pagL* (PA4661) resulted in an increased susceptibility to polymyxins, in a polymyxin-resistant mutant PAK*pmrB6* derived from *P. aeruginosa* PAK [14, 74], demonstrating that the lipid A deacylation also plays a key role in the response of *P. aeruginosa* to polymyxin treatment.

Our recent transcriptomics and metabolomics studies discovered that polymyxin treatment leads to remarkable growth reduction and metabolic perturbations in Gram-negative bacteria [41, 42, 75-77]. The integration of transcriptomics results into GSMMs allow for more accurate predictions of metabolic responses to either environmental (i.e. antibiotic treatment) or genetic perturbations (i.e. mutations) [78]. In the present study, we employed the E-Flux method to integrate transcriptomics data as flux constraints [26]. E-Flux can map continuous gene expression levels to the metabolic network and uses the transcript abundance to determine the degree to which a reaction is active or inactive [26]. Therefore, E-Flux provides a more physiologically relevant description of the continuous nature of the reaction activity and avoids to use any artificial thresholds to binarise gene expression data [79]. In the present study, metabolic fluxes with and without antibiotic treatment were not be calculated with Minimization Of Metabolic Adjustment (MOMA), as MOMA was developed to predict the

metabolic flux redistributions in gene knockout mutants [80]. MOMA hypothesises that metabolism of the mutant tends to approximate the wild-type [80], which is distinct from the antibiotic treatment scenario. For instance, our metabolomics data have demonstrated that the antibiotic treatment caused dramatic metabolic changes in bacteria [41].

Comparison of the calculated flux distributions revealed that a broad range of metabolic perturbations occur in response to polymyxin treatment (**Fig 6**), ranging from central carbon metabolism to oxidative phosphorylation and amino acid metabolism. Reduced growth, increased redox turnover and decreased energy turnover due to polymyxin treatment were evident (**Fig 6**), indicating that bacterial cells regulated their metabolism to produce more redox power to cope with the oxidative stress. This is consistent with previous findings that showed bactericidal antibiotics induced lethal oxidative damages via generating highly deleterious free radicals with subsequent culmination of cellular death [81]. In addition, our simulations revealed that polymyxin treatment induced an uptake of L-alanine, which was catabolised to generate more NADH (**Fig 7**). This indicates that rich media (e.g. CAMHB) may provide abundant amino acids and peptides that can be utilised by bacterial cells to generate sufficient redox equivalents to cope with the oxidative damage caused by polymyxin treatment. Furthermore, our simulation results also showed an upregulated metabolic flux towards L-spermidine biosynthesis upon polymyxin B treatment (rxn00127 and rxn01406, **Additional file 25**). Previous studies showed that polyamines (e.g. spermidine) could protect *P. aeruginosa* from antimicrobial peptide killing [82]. It is assumed that the cationic spermidine could interact with the anionic LPS, mask the negative cell surface, and reduce the electrostatic interactions between polymyxin B and bacterial OM. Therefore, the enhanced biosynthesis of spermidine might increase its abundance at the cell surface and contribute to polymyxin resistance.

The constructed *i*PAO1 provides a detailed presentation of LPS biogenesis (**Fig 2**), in particular lipid A modifications. Further integration with specific regulatory modules will enable

dynamic simulation of metabolic responses to polymyxin treatment. Previous studies revealed that various two-component regulatory systems (2CSs), including PhoPQ, PmrAB, ParRS, CprRS and ColRS, play key roles in regulating polymyxin resistance [69, 83-86]. Among them, the PmrAB and PhoPQ systems are able to sense the depletion of external cations (e.g. Mg^{2+} and Ca^{2+}) and upregulate the expression of the *arnBCADTEF-ugd* operon which is responsible for the modification of lipid A with L-Ara4N [87]. Moreover, the fatty acylation of lipid A by PagP is under the control of PhoPQ [88, 89]. ParRS and CprRS are independent 2CSs that mediate the upregulation of *pmrAB*, *arnBCADTEF-ugd* operon, *pagL* and adaptive resistance in response to polymyxin treatment [83, 90]. In overview, lipid A modifications due to polymyxin treatment are strictly controlled by very complex regulatory networks involving signal sensors, transcriptional regulators, and metabolic enzymes. Therefore, future studies are warranted to integrate these regulatory modules into the GSMM to enable simulating bacterial response dynamics to polymyxin treatment and analysing adaptive resistance mechanisms in *P. aeruginosa*.

Overall, we have constructed, optimised and validated a high-quality genome-scale metabolic model *i*PAO1 for *P. aeruginosa* PAO1. This comprehensive model incorporates metabolic pathways, particularly the biogenesis of membrane components, and enables delineating the complex metabolic responses to antibiotics. *i*PAO1 provides a valuable systems tool for quantitative simulation of bacterial metabolic responses to antibiotics, elucidation of the molecular mechanisms of antimicrobial killing and resistance, and facilitation of designing rational antimicrobial combination therapy. To the best of our knowledge, this study is the first to integrate antimicrobial pharmacology, computational biology, metabolic network and systems pharmacology to analyse large-scale datasets, in order to better understand the dynamic and complex nature of polymyxin killing and resistance. Combined with antibiotic pharmacokinetics and pharmacodynamics, *i*PAO1 offers an *in silico* platform for precision

polymyxin chemotherapy.

Potential implications

The generated collection of transcriptomics metabolomics, lipidomics and lipid A profiling data provides comprehensive datasets of *P. aeruginosa* for future integrative analysis of polymyxin systems pharmacology. As the largest curated GSMM thus far for *Pseudomonas*, *i*PAO1 represents all aspects of the cellular metabolism and may serve as the platform for integrative analysis of multi-omics data. Simulation with transcriptomics constraints in this study revealed metabolic flux changes in amino acid catabolism, tricarboxylic acid cycle, and redox turnover caused by polymyxin treatment. Correlative analysis of metabolomics and transcriptomics with the constraint-based modelling is necessary for delineating the regulatory effects on metabolism. The methodology of using GSMMs to analyse multi-level omics data is applicable to other areas beyond antimicrobial pharmacology. Further integration with antimicrobial pharmacokinetics and pharmacodynamics will not only provide better pharmacological understanding, but also empower the model to quantitatively predict the bacterial responses to antimicrobial therapy in the context of complex interplay of signalling, transcriptional regulation and metabolism. In summary, our GSMM approach provides a powerful systems tool to elucidate the complex mode of action of antibiotics and will paradigm shift antimicrobial pharmacology.

Methods

Strain, media and BIOLOG experiments

P. aeruginosa PAO1 was cultured in Luria-Bertani (LB) media and subcultured on nutrient agar. Cells were swapped into sterile capped tube containing 16 mL IF-0 solution (Cell

Biosciences, West Heidelberg, Australia) till the turbidity achieved 42% transmittance in a Turbidimeter (Pacificlab, Blackburn, Australia). The cell suspension was then diluted 5 times with IF-0 solution and dye (Cell Biosciences) to final 85% transmittance. BIOLOG PM 1-3 (Cell Biosciences, Heidelberg, Australia) were used to investigate the carbon and nitrogen utilisation with two independent biological replicates. Sodium succinate was used as the carbon source for examining nitrogen utilization. Growth was detected after 24-h incubation at 37°C, using an Infinite M200 microplate reader (Tecan, Mannedorf, Switzerland) at 595 nm. Readings that were ≥1.5-fold of the negative control (i.e. growth media without bacteria) indicated the utilisation of nutrients.

Development of a GSMM for *P. aeruginosa* PAO1

To expedite the model development, two curated models for PAO1 with the same identifier systems from Model SEED [32], iMO1056 [28] and Opt20896434 [29] were merged. Databases including KEGG [44], MetaCyc [45], Pseudomonas Genome DB [48] and the literature were employed to complete the model with missing components. The identifiers of metabolites and reactions were kept consistent with Model SEED [29], and cross-referred to MetaCyc, KEGG, PubChem [64], ChEBI [66], ChemSpider [65] and BiGG [67]. The PAO1 genome annotation from Pseudomonas Genome DB [48] was employed to construct 'gene to protein to reaction' (GPR) associations [91]. A periplasm compartment was incorporated into the model. Reactions and metabolites were then assigned to cytoplasm, periplasm and external environment according to the localisation prediction of metabolic enzymes by PSORTb 3.0 [92]. Transport reactions were generated to enable material exchange across membranes according to TCBD [46] and TransporterDB [47]. The model was constructed using the Systems Biology Markup Language (SBML) [93, 94]. VANTED [95] was employed for visualisation and analysis of the metabolic network. For each metabolite in the model, specific features including compartment localisation, mass, charge, formula, formation free energy,

database identifiers and source were added (**Additional file 14**). Each reaction entered into the model was checked with elementary and charge balance. Reversibility was determined first from the primary literature for each particular enzyme or reaction, if available. Further curation on reaction reversibility and directions was conducted based on change of free energy and knowledge about the physiological direction of a reaction in a pathway.

The Gapfind function from the COBRA toolbox [96] was employed to identify the isolated and dead-end metabolites in the model. Candidate reactions from KEGG, MetaCyc and BiGG were manually inspected for relevance and homology evidence using BLASTp; reactions catalysed by homologous enzymes (E-value<1×10^{-5}, identity≥35%, coverage≥50%) were added to the model to eliminate the gaps. Mispredictions of BIOLOG growth phenotypes were employed to refine the draft model (*i*PAO1_draft2). Further curation was performed to represent the complex biosynthesis pathways of macromolecules (e.g. peptidoglycan, GPL and LPS).

The biomass formation equation consisting of necessary building blocks for bacterial growth was created using the one from iMO1086 [30], with slight modifications on compositions of ions, peptidoglycans, GPL and LPS (**Additional file 17**). The growth and non-growth associated maintenance was from iMO1086 [30].

Growth prediction in BIOLOG media

*i*PAO1 was employed to predict the growth phenotypes on chemically-defined media with 190 carbon and 95 nitrogen sources (**Additional file 18**) using the FBA method [24]. The objective function of biomass formation was maximised with the specific nutrient uptake rate set at 10 mmol·gDW^{-1}·h^{-1} under aerobic condition.

$$\max \quad v_{\mathrm{biomass}}$$

$$s.t. \quad \mathbf{Sv} = 0$$

$$a_i \le v_i \le b_i, i = 1,2,\cdots,n$$

where v_{biomass} denotes the biomass formation flux, **S** represents the stoichiometric matrix and each metabolic flux v_i was constrained by lower and upper bound a_i and b_i, respectively. All modelling procedures were performed with the COBRA toolbox [96] in MATLAB. The calculated specific growth rates v_{biomass} were then compared to the BIOLOG PM data to assess the prediction accuracy using Fisher's exact test.

Gene essentiality prediction

In silico single-gene deletion was performed using the CORBRA toolbox and the mutant models were then used to predict the specific growth rate in LB broth [32] using FBA. Genes with 99% reduction of the specific growth rate relative to the wild type were defined as essential for cell growth; otherwise, they were considered as semi-essential (1-99% reduction) and non-essential (<1% reduction). Two existing PAO1 transposon insertion mutant libraries, (i) two-allele mutant library [50, 68] and (ii) mini-Tn5 insertion mutant library [49], were employed to assess the overall prediction accuracy with Fisher's exact test. To determine polymyxin-specific essential genes, transcriptomic constrains were imposed (below) before conducting *in silico* single-gene deletion simulations. The calculated essential genes identified in polymyxin treatment alone but not in the control were considered as polymyxin-specific.

Simulation of bacterial growth and metabolic phenotype changes in response to lipid A modifications

The LPS stoichiometric coefficients in the biomass formula under the control and lipid A modification conditions were set according to the measured lipid A compositions in the wild-type *P. aeruginosa* PAK and its polymyxin-resistant mutant PAK*pmrB6*, respectively (**Table 2**) [14]. For PAKpmrB6, a missense mutation (L243Q) in *pmrB* resulted in constitutive activation of the PmrAB system and induced expression of the regulated genes regardless of polymyxin, including *arn* operon and *pagL* [42, 97]. Aerobic growth was simulated on minimal

media with glucose uptake at 10 mmol·gDW^{-1}·h^{-1}. For each simulation, the solution space was sampled with 10,000 random points using the ll-ACHRB algorithm [98]. Flux samples of the control and lipid A modification were then compared. Significantly perturbed metabolic fluxes were identified using a *Z*-score based approach [71].

To further analyse the metabolic impact of lipid A modifications, the proportions of all types of LPS in the biomass formula were randomly assigned and the process was repeated 1,000 times. For each repetition, the specific growth rates were calculated and solution space was sampled using the methods above. For each type of lipid A, specific physiochemical properties (f) including total atom number, partition coefficient (logP), average charge and molecular polarity were predicted at pH 7 using the cxcalc tool from ChemAxon (Budapest, Hungary). The overall apparent properties F of the OM were estimated by calculating the weighted sum.

$$F = \sum_{j=1}^{n} w_j f_j$$

where w_j represents the stoichiometric coefficient of the j-th of 288 heterogeneous LPS molecules in the biomass formula. Pairwise correlation analysis was conducted between lipid A modifications, physiochemical properties changes, bacterial growth and metabolism alterations.

Predict metabolic responses to polymyxin treatment by constraining fluxes with transcriptomics data

The RNA-Seq data from 1-h 1 mg·L^{-1} polymyxin B treatment experiment using PAO1 were employed as flux constraints for modelling [51]. For each gene under every condition, the RPKM (Reads Per Kilobase Million) value was calculated from the aligned reads using the edgeR package [99], and normalised to constrain flux upper bounds (b_i) using the E-Flux algorithm [26]. Specifically, for each reaction catalysed by a single enzyme, the upper flux

bound was set to the determined RPKM value under the respective condition. For a reaction catalysed by an enzyme complex, the upper bound was set to the minimum RPKM value of the associated genes. For a reaction catalysed by isozymes, the upper bound was set to the sum of RPKM values of the associated genes. The maximum of upper bounds was then normalised to 10,000 $mmol \cdot gDW^{-1} \cdot h^{-1}$. The lower bounds a_i were set to 0 for irreversible and $-b_i$ $mmol \cdot gDW^{-1} \cdot h^{-1}$ for reversible reactions, respectively. CAMHB was used in the RNA-Seq experiment and it is known as an undefined medium containing mainly amino acids and oligopeptides [100]. The maximum uptake rates of amino acids in *P. aeruginosa* vary between 0.26-1.44 $mmol \cdot gDW^{-1} \cdot h^{-1}$ [101-103]. Therefore, the upper bounds (b_i^{CAMHB}) of uptake rates of amino acids, vitamins and dipeptides in *i*PAO1 were constrained to 1 $mmol \cdot gDW^{-1} \cdot h^{-1}$ without loss of generality. For each condition, the solution space was sampled with 10,000 points using ll-ACHRB as above. Statistical significance of differential flux distributions was computed using the *Z*-score method above. The turnover rate for key metabolites was calculated by summing up all influxes or effluxes [104]. To assess the impact of changing nutrient uptake bounds, sensitivity analysis was conducted by randomly sampling solution space as above while varying b_i^{CAMHB} from 0.26 to 1.44 $mmol \cdot gDW^{-1} \cdot h^{-1}$.

Availability of supporting data and materials

The raw RNA-Seq data have been deposited in the NCBI Sequence Read Archive (SRA) database under the BioProject accession number PRJNA414673. The metabolomics and lipidomics data have been deposited in the Metabolight database with the accession number MTBLS630. Supporting data, also including the scripts used in this project, are available via the *GigaScience* repository GigaDB[106].

Additional files

Additional file 1 (additionalFile1.docx): Manual curation of GPL biosynthesis, LPS biosynthesis and modification pathways, and sensitivity analysis of nutrient uptake bounds.

Additional file 2 (additionalFile2.xlsx): Metabolites in the constructed draft model *i*PAO1_draft1.

Additional file 3 (additionalFile3.xlsx): Reactions in the constructed draft model *i*PAO1_draft1.

Additional file 4 (additionalFile4.xlsx): Genes in the constructed draft model *i*PAO1_draft1.

Additional file 5 (additionalFile5.xlsx). Supplemented metabolites according to previous GC-MS based metabolomics data.

Additional file 6 (additionalFile6.xlsx): Supplemented reactions according to previous GS-MS based metabolomics data.

Additional file 7 (additionalFile7.xlsx): Root gap metabolites identified using GapFind from the COBRA toolbox.

Additional file 8 (additionalFile8.xlsx): Comparison of the predicted growth phenotypes with the BIOLOG PM results.

Additional file 9 (additionalFile9.xlsx): Reactions with changed reversibility and directionality during manual curation.

Additional file 10 (additionalFile10.xlsx): Deleted metabolites during manual curation.

Additional file 11 (additionalFile11.xlsx): Deleted reactions during manual curation.

Additional file 12 (additionalFile12.xlsx): Added reactions during manual curation.

Additional file 13 (additionalFile13.xlsx): Added intermediate metabolites in GPL biosynthesis pathway.

Additional file 14 (additionalFile14.xlsx): Added reactions in GPL biosynthesis pathway.

Additional file 15 (additionalFile15.xlsx): Metabolites in the constructed model *i*PAO1.

Additional file 16 (additionalFile16.xlsx): Reactions in the constructed model *i*PAO1.

Additional file 17 (additionalFile17.xlsx): Genes in the constructed model *i*PAO1.

Additional file 18 (additionalFile18.xlsx): Biomass formation formula.

Additional file 19 (additionalFile19.xlsx): Comparison of the predicted gene essentiality with the information derived from two transposon insertion mutant libraries.

Additional file 20 (additionalFile20.xlsx): Metabolic flux changes in response to lipid A modifications using lipidomics data as stoichiometric constraints.

Additional file 21 (additionalFile21.xlsx): Randomised stoichiometric coefficients of LPS species.

Additional file 22 (additionalFile22.xlsx): Predicted physiochemical properties of lipid A molecules.

Additional file 23 (additionalFile23.xlsx): Metabolic flux changes in response to lipid A modifications with randomly assigned lipid A compositions as stoichiometric constraints.

Additional file 24 (additionalFile24.xlsx): Metabolic flux constraints calculated based on RNA-Seq data.

Additional file 25 (additionalFile25.xlsx): Metabolic flux changes in response to polymyxin treatment using RNA-Seq data as flux constraints.

Additional file 26 (additionalFile26.xlsx): Metabolite turnover rates.

Additional file 27 (additionalFile27.xlsx): Full names of the metabolite abbreviations in Figure 1.

List of abbreviations

2CS: two-component regulatory system; CAMHB: cation-adjusted Mueller-Hinton broth; COG: clusters of orthologous groups; FBA: flux balance analysis; FDR: false discovery rate; GC-MS: gas chromatography-mass spectrometry; GPL: Glycerolphospholipid; GPR: gene to protein to reaction; GSMM: genome-scale metabolic models; IM: inner membrane; KEGG: Kyoto Encyclopaedia of Genes and Genomes; L-Ara4N: 4-Amino-4-deoxy-L-arabinose; LB: Luria-Bertani; LC-MS: liquid chromatography-mass spectrometry; LPS: lipopolysaccharide; MDR: Multidrug-resistant; OM: outer membrane; PM: Phenotypic Microarray; RPKM: Reads Per Kilobase Million; SBML: Systems Biology Markup Language; SRA: Sequence Read Archive; TCDB: Transporter Classification Database.

Competing interests

The authors declare no competing interest for this work.

Funding

This study was partially supported by a Major Interdisciplinary Research Grant from Monash University and a project grant by the Australian National Health and Medical Research Council (NHMRC, APP1127948). J.L., T.V., and J.S. are supported by the National Institute of Allergy and Infectious Diseases of the National Institutes of Health (R01 AI111965). The content is solely the responsibility of the authors and does not necessarily represent the official views of the National Institute of Allergy and Infectious Diseases or the National Institutes of Health. T.V. is an Australian NHMRC Career Development Research Fellow. T.L. is an Australian Laureate Fellow supported by Australian Research Council. J.L. is an Australian NHMRC

Senior Research Fellow.

Authors' contributions

J.L. and F.S. conceived the project and Y.Z. developed the GSMM and conducted most analysis. T.C. and M.K. validated the model. J.Z., J.Lu and B.S. curated the model. T.V., T.L. and J.S. helped supervise the project. M.H. and M.H.M.M. provided the lipidomics and transcriptomics data, respectively.

Acknowledgements

The authors acknowledge the assistance of Dr Darren Creek from the Monash Institute of Pharmaceutical Sciences in LC-MS experiments.

References

1. Scales BS, Dickson RP, LiPuma JJ and Huffnagle GB. Microbiology, genomics, and clinical significance of the *Pseudomonas fluorescens* species complex, an unappreciated colonizer of humans. Clin Microbiol Rev. 2014;27:927-48.
2. Breidenstein EB, de la Fuente-Nunez C and Hancock RE. *Pseudomonas aeruginosa*: all roads lead to resistance. Trends Microbiol. 2011;19:419-26.
3. Liu YY, Wang Y, Walsh TR, Yi LX, Zhang R, Spencer J, et al. Emergence of plasmid-mediated colistin resistance mechanism MCR-1 in animals and human beings in China: a microbiological and molecular biological study. Lancet Infect Dis. 2016;16:161-8.
4. Winstanley C, O'Brien S and Brockhurst MA. *Pseudomonas aeruginosa* evolutionary adaptation and diversification in cystic fibrosis chronic lung infections. Trends Microbiol. 2016;24:327-37.
5. de Almeida Silva KCF, Calomino MA, Deutsch G, de Castilho SR, de Paula GR, Esper LMR, et al. Molecular characterization of multidrug-resistant (MDR) *Pseudomonas aeruginosa* isolated in a burn center. Burns. 2017;43:137-43.
6. Church D, Elsayed S, Reid O, Winston B and Lindsay R. Burn wound infections. Clin Microbiol Rev. 2006;19:403-34.
7. Klockgether J, Cramer N, Wiehlmann L, Davenport CF and Tummler B. *Pseudomonas aeruginosa* genomic structure and diversity. Front Microbiol. 2011;2:150.
8. Ramos JL and Filloux A. Pseudomonas: Volume 5: A model system in biology. London: Springer; 2007.
9. Nation RL, Li J, Cars O, Couet W, Dudley MN, Kaye KS, et al. Framework for optimisation of the clinical use of colistin and polymyxin B: the Prato polymyxin consensus. Lancet Infect Dis. 2015;15:225-34.

10. Pedersen MG, Jensen-Fangel S, Olesen HV, Nørskov-Lauritsen N and Wang M. 129 Colistin resistance in *Achromobacter* sp. and *Pseudomonas aeruginosa* isolated from Danish cystic fibrosis patients is not related to plasmid-mediated expression of *mcr-1*. J Cyst Fibros. 2017;16:S98.

11. Wi YM, Choi JY, Lee JY, Kang CI, Chung DR, Peck KR, et al. Emergence of colistin resistance in *Pseudomonas aeruginosa* ST235 clone in South Korea. Int J Antimicrob Agents. 2017;49:767-9.

12. Yu Z, Qin W, Lin J, Fang S and Qiu J. Antibacterial mechanisms of polymyxin and bacterial resistance. Biomed Res Int. 2015;2015:679109-19.

13. Trimble MJ, Mlynarcik P, Kolar M and Hancock RE. Polymyxin: alternative mechanisms of action and resistance. Cold Spring Harb Perspect Med. 2016;6:a025288.

14. Han M, Zhu Y, Cheah SE, Johnson MD, Yu H, Shen HH, et al. Polymyxin resistance in *Pseudomonas aeruginosa*: metabolomic changes underpin lipid A modifications. In: *ASM Microbe 2017* Boston, USA, 2016, p.491.

15. Hussein MH, Maifiah MHM, Han M, Tran TB, Zhu Y, Hancock REW, et al. Mechanisms of synergistic killing against *Pseudomonas aeruginosa* by polymyxin B and amikacin: A metabolomics study. In: *European Congress of Clinical Microbiology and Infectious Diseases* Vienna, Austria, 2017, p.EV0387. ESCMID.

16. O'Brien EJ, Monk JM and Palsson BO. Using genome-scale models to predict biological capabilities. Cell. 2015;161:971-87.

17. Hohenschuh W, Hector R and Murthy GS. A dynamic flux balance model and bottleneck identification of glucose, xylose, xylulose co-fermentation in *Saccharomyces cerevisiae*. Bioresour Technol. 2015;188:153-60.

18. Hanly TJ and Henson MA. Dynamic metabolic modeling of a microaerobic yeast co-culture: predicting and optimizing ethanol production from glucose/xylose mixtures. Biotechnol Biofuels. 2013;6:44.

19. Hanly TJ and Henson MA. Dynamic flux balance modeling of microbial co-cultures for efficient batch fermentation of glucose and xylose mixtures. Biotechnol Bioeng. 2011;108:376-85.

20. Bosi E, Monk JM, Aziz RK, Fondi M, Nizet V and Palsson BO. Comparative genome-scale modelling of *Staphylococcus aureus* strains identifies strain-specific metabolic capabilities linked to pathogenicity. Proc Natl Acad Sci U S A. 2016;113:E3801-9.

21. Kim HU, Kim SY, Jeong H, Kim TY, Kim JJ, Choy HE, et al. Integrative genome-scale metabolic analysis of *Vibrio vulnificus* for drug targeting and discovery. Mol Syst Biol. 2011;7:460.

22. Krueger AS, Munck C, Dantas G, Church GM, Galagan J, Lehar J, et al. Simulating serial-target antibacterial drug synergies using flux balance analysis. PLoS One. 2016;11:e0147651.

23. Aziz RK, Monk JM, Lewis RM, In Loh S, Mishra A, Abhay Nagle A, et al. Systems biology-guided identification of synthetic lethal gene pairs and its potential use to discover antibiotic combinations. Sci Rep. 2015;5:16025.

24. Bordbar A, Monk JM, King ZA and Palsson BO. Constraint-based models predict metabolic and associated cellular functions. Nat Rev Genet. 2014;15:107-20.

25. Presta L, Bosi E, Mansouri L, Dijkshoorn L, Fani R and Fondi M. Constraint-based modeling identifies new putative targets to fight colistin-resistant *A. baumannii* infections. Sci Rep. 2017;7:3706.

26. Colijn C, Brandes A, Zucker J, Lun DS, Weiner B, Farhat MR, et al. Interpreting expression data with metabolic flux models: predicting *Mycobacterium tuberculosis* mycolic acid production. PLoS Comput Biol. 2009;5:e1000489.

27. Navid A and Almaas E. Genome-level transcription data of *Yersinia pestis* analyzed with a new metabolic constraint-based approach. BMC Syst Biol. 2012;6:150.

28. Oberhardt MA, Puchalka J, Fryer KE, Martins dos Santos VA and Papin JA. Genome-scale metabolic network analysis of the opportunistic pathogen *Pseudomonas aeruginosa* PAO1. J Bacteriol. 2008;190:2790-803.

29. Henry CS, DeJongh M, Best AA, Frybarger PM, Linsay B and Stevens RL. High-throughput generation, optimization and analysis of genome-scale metabolic models. Nat Biotechnol. 2010;28:977-82.

30. Oberhardt MA, Puchalka J, Martins dos Santos VA and Papin JA. Reconciliation of genome-scale metabolic reconstructions for comparative systems analysis. PLoS Comput Biol. 2011;7:e1001116.

31. Bartell JA, Blazier AS, Yen P, Thogersen JC, Jelsbak L, Goldberg JB, et al. Reconstruction of the metabolic network of *Pseudomonas aeruginosa* to interrogate virulence factor synthesis. Nat Commun. 2017;8:14631.

32. Devoid S, Overbeek R, DeJongh M, Vonstein V, Best AA and Henry C. Automated genome annotation and metabolic model reconstruction in the SEED and Model SEED. Methods Mol Biol. 2013;985:17-45.

33. Oberhardt MA, Goldberg JB, Hogardt M and Papin JA. Metabolic network analysis of *Pseudomonas aeruginosa* during chronic cystic fibrosis lung infection. J Bacteriol. 2010;192:5534-48.

34. Biggs MB and Papin JA. Novel multiscale modeling tool applied to *Pseudomonas aeruginosa* biofilm formation. PLoS One. 2013;8:e78011.

35. Vital-Lopez FG, Reifman J and Wallqvist A. Biofilm formation mechanisms of *Pseudomonas aeruginosa* predicted via genome-scale kinetic models of bacterial metabolism. PLoS Comput Biol. 2015;11:e1004452.

36. Sigurdsson G, Fleming RM, Heinken A and Thiele I. A systems biology approach to drug targets in *Pseudomonas aeruginosa* biofilm. PLoS One. 2012;7:e34337.

37. Xu Z, Fang X, Wood TK and Huang ZJ. A systems-level approach for investigating *Pseudomonas aeruginosa* biofilm formation. PLoS One. 2013;8:e57050.

38. Perumal D, Samal A, Sakharkar KR and Sakharkar MK. Targeting multiple targets in *Pseudomonas aeruginosa* PAO1 using flux balance analysis of a reconstructed genome-scale metabolic network. J Drug Target. 2011;19:1-13.

39. Dalebroux ZD, Matamouros S, Whittington D, Bishop RE and Miller SI. PhoPQ regulates acidic glycerophospholipid content of the *Salmonella Typhimurium* outer membrane. Proc Natl Acad Sci U S A. 2014;111:1963-8.

40. Raetz CR, Reynolds CM, Trent MS and Bishop RE. Lipid A modification systems in Gram-negative bacteria. Annu Rev Biochem. 2007;76:295-329.

41. Maifiah MH, Creek DJ, Nation RL, Forrest A, Tsuji BT, Velkov T, et al. Untargeted metabolomics analysis reveals key pathways responsible for the synergistic killing of colistin and doripenem combination against *Acinetobacter baumannii*. Sci Rep. 2017;7:45527.

42. Han ML, Zhu Y, Cheah S-E, Johnson MD, Yu HH, Maifiah MHM, et al. Polymyxin resistance due to mutations in *pmrB* caused global metabolomics changes in *Pseudomonas aeruginosa*. In: *The Australian & New Zealand Metabolomics Conference* Melbourne, Australia, 30 March 2016, p.106.

43. Frimmersdorf E, Horatzek S, Pelnikevich A, Wiehlmann L and Schomburg D. How *Pseudomonas aeruginosa* adapts to various environments: a metabolomic approach. Environ Microbiol. 2010;12:1734-47.

44. Kanehisa M, Sato Y, Kawashima M, Furumichi M and Tanabe M. KEGG as a reference resource for gene and protein annotation. Nucleic Acids Res. 2016;44:D457-62.

45. Caspi R, Billington R, Ferrer L, Foerster H, Fulcher CA, Keseler IM, et al. The MetaCyc database of metabolic pathways and enzymes and the BioCyc collection of pathway/genome databases. Nucleic Acids Res. 2016;44:D471-80.

46. Saier MH, Jr., Reddy VS, Tsu BV, Ahmed MS, Li C and Moreno-Hagelsieb G. The Transporter Classification Database (TCDB): recent advances. Nucleic Acids Res. 2016;44:D372-9.

47. Elbourne LD, Tetu SG, Hassan KA and Paulsen IT. TransportDB 2.0: a database for exploring membrane transporters in sequenced genomes from all domains of life. Nucleic Acids Res. 2017;45:D320-D4.

48. Winsor GL, Griffiths EJ, Lo R, Dhillon BK, Shay JA and Brinkman FS. Enhanced annotations and features for comparing thousands of *Pseudomonas* genomes in the *Pseudomonas* genome database. Nucleic Acids Res. 2016;44:D646-53.

49. Lewenza S, Falsafi RK, Winsor G, Gooderham WJ, McPhee JB, Brinkman FS, et al. Construction of a mini-Tn5-*luxCDABE* mutant library in *Pseudomonas aeruginosa* PAO1: a tool for identifying differentially regulated genes. Genome Res. 2005;15:583-9.

50. Held K, Ramage E, Jacobs M, Gallagher L and Manoil C. Sequence-verified two-allele transposon mutant library for *Pseudomonas aeruginosa* PAO1. J Bacteriol. 2012;194:6387-9.

51. Maifiah MHM. *Deciphering the modes of action of polymyxins and the synergistic combinations against multidrug-resistant Gram-negative bacteria: a systems pharmacology approach*. PhD thesis, Monash University, Australia, 2017.

52. Liao Y, Smyth GK and Shi W. The Subread aligner: fast, accurate and scalable read mapping by seed-and-vote. Nucleic Acids Res. 2013;41:e108.

53. Powell D: Degust. http://degust.erc.monash.edu/ (2017). Accessed Oct 18 2017.

54. Creek DJ, Jankevics A, Burgess KE, Breitling R and Barrett MP. IDEOM: an Excel interface for analysis of LC-MS-based metabolomics data. Bioinformatics. 2012;28:1048-9.

55. Satish Kumar V, Dasika MS and Maranas CD. Optimization based automated curation of metabolic reconstructions. BMC Bioinformatics. 2007;8:212.

56. Finn RD, Coggill P, Eberhardt RY, Eddy SR, Mistry J, Mitchell AL, et al. The Pfam protein families database: towards a more sustainable future. Nucleic Acids Res. 2016;44:D279-85.

57. Zdobnov EM, Tegenfeldt F, Kuznetsov D, Waterhouse RM, Simao FA, Ioannidis P, et al. OrthoDB v9.1: cataloging evolutionary and functional annotations for animal, fungal, plant, archaeal, bacterial and viral orthologs. Nucleic Acids Res. 2017;45:D744-D9.

58. Hempel N, Görisch H and Mern DS. Gene *ercA*, encoding a putative iron-containing alcohol dehydrogenase, is involved in regulation of ethanol utilization in *Pseudomonas aeruginosa*. J Bacteriol. 2013;195:3925-32.

59. Kohanski MA, Dwyer DJ and Collins JJ. How antibiotics kill bacteria: from targets to networks. Nat Rev Microbiol. 2010;8:423-35.

60. Typas A, Banzhaf M, Gross CA and Vollmer W. From the regulation of peptidoglycan synthesis to bacterial growth and morphology. Nat Rev Microbiol. 2011;10:123-36.

61. Cox E, Michalak A, Pagentine S, Seaton P and Pokorny A. Lysylated phospholipids stabilize models of bacterial lipid bilayers and protect against antimicrobial peptides. Biochim Biophys Acta. 2014;1838:2198-204.

62. Kondakova T, D'Heygere F, Feuilloley MJ, Orange N, Heipieper HJ and Duclairoir Poc C. Glycerophospholipid synthesis and functions in *Pseudomonas*. Chem Phys Lipids. 2015;190:27-42.

63. Velkov T, Thompson PE, Nation RL and Li J. Structure−activity relationships of polymyxin antibiotics. J Med Chem. 2010;53:1898-916.

64. Kim S, Thiessen PA, Bolton EE, Chen J, Fu G, Gindulyte A, et al. PubChem Substance and Compound databases. Nucleic Acids Res. 2016;44:D1202-13.

65. Pence HE and Williams A. ChemSpider: an online chemical information resource. J Chem Educ. 2010;87:1123-4.

66. Hastings J, de Matos P, Dekker A, Ennis M, Harsha B, Kale N, et al. The ChEBI reference database and ontology for biologically relevant chemistry: enhancements for 2013. Nucleic Acids Res. 2013;41:D456-63.

67. King ZA, Lu J, Drager A, Miller P, Federowicz S, Lerman JA, et al. BiGG Models: A platform for integrating, standardizing and sharing genome-scale models. Nucleic Acids Res. 2016;44:D515-22.

68. Jacobs MA, Alwood A, Thaipisuttikul I, Spencer D, Haugen E, Ernst S, et al. Comprehensive transposon mutant library of *Pseudomonas aeruginosa*. Proc Natl Acad Sci U S A. 2003;100:14339-44.

69. Gutu AD, Sgambati N, Strasbourger P, Brannon MK, Jacobs MA, Haugen E, et al. Polymyxin resistance of *Pseudomonas aeruginosa phoQ* mutants is dependent on additional two-component regulatory systems. Antimicrob Agents Chemother. 2013;57:2204-15.

70. Olaitan AO, Morand S and Rolain JM. Mechanisms of polymyxin resistance: acquired and intrinsic resistance in bacteria. Front Microbiol. 2014;5:643.

71. Mo ML, Palsson BO and Herrgard MJ. Connecting extracellular metabolomic measurements to intracellular flux states in yeast. BMC Syst Biol. 2009;3:37.

72. Graham LL, Beveridge TJ and Nanninga N. Periplasmic space and the concept of the periplasm. Trends Biochem Sci. 1991;16:328-9.

73. Silhavy TJ, Kahne D and Walker S. The bacterial cell envelope. Cold Spring Harb Perspect Biol. 2010;2:a000414.

74. Han M, Shen HH, Zhu Y, Roberts KD, Le Brun AP, Moskowitz SM, et al. Deciphering the mechanisms of polymyxin resistance in *Pseudomonas aeruginosa*: A systems pharmacology and neutron reflectometry approach. In: *Solutions for Drug-Resistant Infections (SDRI) 2017* Brisbane, Australia, 3-5, April 2017, p.P23. SDRI 2017.

75. Maifiah MH, Cheah SE, Johnson MD, Han ML, Boyce JD, Thamlikitkul V, et al. Global metabolic analyses identify key differences in metabolite levels between polymyxin-susceptible and polymyxin-resistant *Acinetobacter baumannii*. Sci Rep. 2016;6:22287.

76. Henry R, Crane B, Powell D, Deveson Lucas D, Li Z, Aranda J, et al. The transcriptomic response of *Acinetobacter baumannii* to colistin and doripenem alone and in combination in an *in vitro* pharmacokinetics/pharmacodynamics model. J Antimicrob Chemother. 2015;70:1303-13.

77. Abdul Rahim N, Cheah S, Zhu Y, Johnson M, Boyce J, Yu H, et al. Integrative multi-omics network analysis of the synergistic killing of polymyxin B and chloramphenicol combination against an NDM-producing *Klebsiella pneumoniae* isolate. In: *2016 European Congress of Clinical Microbiology and Infectious Diseases (ECCMID)* Amsterdam, Netherland, 2016, p.EV0651. ESCMID.

78. Machado D and Herrgard M. Systematic evaluation of methods for integration of transcriptomic data into constraint-based models of metabolism. PLoS Comput Biol. 2014;10:e1003580.

79. Blazier AS and Papin JA. Integration of expression data in genome-scale metabolic network reconstructions. Front Physiol. 2012;3:299.

80. Segre D, Vitkup D and Church GM. Analysis of optimality in natural and perturbed metabolic networks. Proc Natl Acad Sci U S A. 2002;99:15112-7.

81. Kohanski MA, Dwyer DJ, Hayete B, Lawrence CA and Collins JJ. A common mechanism of cellular death induced by bactericidal antibiotics. Cell. 2007;130:797-810.

82. Johnson L, Mulcahy H, Kanevets U, Shi Y and Lewenza S. Surface-localized spermidine protects the *Pseudomonas aeruginosa* outer membrane from antibiotic treatment and oxidative stress. J Bacteriol. 2012;194:813-26.

83. Fernandez L, Jenssen H, Bains M, Wiegand I, Gooderham WJ and Hancock RE. The two-component system CprRS senses cationic peptides and triggers adaptive resistance in *Pseudomonas aeruginosa* independently of ParRS. Antimicrob Agents Chemother. 2012;56:6212-22.

84. Barrow K and Kwon DH. Alterations in two-component regulatory systems of *phoPQ* and *pmrAB* are associated with polymyxin B resistance in clinical isolates of *Pseudomonas aeruginosa*. Antimicrob Agents Chemother. 2009;53:5150-4.

85. Owusu-Anim D and Kwon DH. Differential role of two-component regulatory systems (*phoPQ* and *pmrAB*) in polymyxin B susceptibility of *Pseudomonas aeruginosa*. Adv Microbiol. 2012;2:31-6.

86. Moskowitz SM, Ernst RK and Miller SI. PmrAB, a two-component regulatory system of *Pseudomonas aeruginosa* that modulates resistance to cationic antimicrobial peptides and addition of aminoarabinose to lipid A. J Bacteriol. 2004;186:575-9.

87. Winfield MD and Groisman EA. Phenotypic differences between *Salmonella* and *Escherichia coli* resulting from the disparate regulation of homologous genes. Proc Natl Acad Sci U S A. 2004;101:17162-7.

88. McPhee JB, Bains M, Winsor G, Lewenza S, Kwasnicka A, Brazas MD, et al. Contribution of the PhoP-PhoQ and PmrA-PmrB two-component regulatory systems to Mg^{2+}-induced gene regulation in *Pseudomonas aeruginosa*. J Bacteriol. 2006;188:3995-4006.

89. Thaipisuttikul I, Hittle LE, Chandra R, Zangari D, Dixon CL, Garrett TA, et al. A divergent *Pseudomonas aeruginosa* palmitoyltransferase essential for cystic fibrosis-specific lipid A. Mol Microbiol. 2014;91:158-74.

90. Fernández L, Gooderham WJ, Bains M, McPhee JB, Wiegand I and Hancock REW. Adaptive resistance to the “last hope” antibiotics polymyxin B and colistin in *Pseudomonas aeruginosa* is mediated by the novel two-component regulatory system ParR-ParS. Antimicrob Agents Chemother. 2010;54:3372-82.

91. Thiele I and Palsson BO. A protocol for generating a high-quality genome-scale metabolic reconstruction. Nat Protoc. 2010;5:93-121.

92. Yu NY, Wagner JR, Laird MR, Melli G, Rey S, Lo R, et al. PSORTb 3.0: improved protein subcellular localization prediction with refined localization subcategories and predictive capabilities for all prokaryotes. Bioinformatics. 2010;26:1608-15.

93. Hucka M, Finney A, Sauro HM, Bolouri H, Doyle JC, Kitano H, et al. The systems biology markup language (SBML): a medium for representation and exchange of biochemical network models. Bioinformatics. 2003;19:524-31.

94. Hucka M and Finney AM: Systems Biology Markup Language (SBML) Level 2: Structures and Facilities for Model Definitions. http://identifiers.org/combine.specifications/sbml.level-2.version-1 (2003). Accessed Oct 18 2017.

95. Rohn H, Junker A, Hartmann A, Grafahrend-Belau E, Treutler H, Klapperstuck M, et al. VANTED v2: a framework for systems biology applications. BMC Syst Biol. 2012;6:139.

96. Schellenberger J, Que R, Fleming RM, Thiele I, Orth JD, Feist AM, et al. Quantitative prediction of cellular metabolism with constraint-based models: the COBRA Toolbox v2.0. Nat Protoc. 2011;6:1290-307.

97. Moskowitz SM, Brannon MK, Dasgupta N, Pier M, Sgambati N, Miller AK, et al. PmrB mutations promote polymyxin resistance of *Pseudomonas aeruginosa* isolated from colistin-treated cystic fibrosis patients. Antimicrob Agents Chemother. 2012;56:1019-30.

98. Saa PA and Nielsen LK. ll-ACHRB: a scalable algorithm for sampling the feasible solution space of metabolic networks. Bioinformatics. 2016;32:2330-7.

99. McCarthy DJ, Chen Y and Smyth GK. Differential expression analysis of multifactor RNA-Seq experiments with respect to biological variation. Nucleic Acids Res. 2012;40:4288-97.

100. Mueller JH and Hinton J. A protein-free medium for primary isolation of the *Gonococcus* and Meningococcus. Proc Soc Exp Biol Med. 1941;48:330-3.

101. Hoshino T. Transport systems for branched-chain amino acids in *Pseudomonas aeruginosa*. J Bacteriol. 1979;139:705-12.

102. Yoshimura F and Nikaido H. Permeability of *Pseudomonas aeruginosa* outer membrane to hydrophilic solutes. J Bacteriol. 1982;152:636-42.

103. Kay WW and Gronlund AF. Transport of aromatic amino acids by *Pseudomonas aeruginosa*. J Bacteriol. 1971;105:1039-46.

104. Kim PJ, Lee DY, Kim TY, Lee KH, Jeong H, Lee SY, et al. Metabolite essentiality elucidates robustness of *Escherichia coli* metabolism. Proc Natl Acad Sci U S A. 2007;104:13638-42.

105. Benjamini Y and Hochberg Y. Controlling the false discovery rate: A practical and powerful approach to multiple testing. J Roy Stat Soc B Met. 1995;57:289-300.

106 Zhu Y, Czauderna T, Zhao J, Klapperstueck M, Maifiah MH, Han ML et al. Supporting data for "Genome-scale metabolic modelling of responses to polymyxins in Pseudomonas aeruginosa." *Gigascience Database* 2018. http://dx.doi.org/10.5524/100414

Figure legends

Figure 1. The curated GPL biosynthesis in *i*PAO1. [c], intracellular metabolites; [p], periplasmic metabolites; [e], external metabolites. Blue arrows indicate transport reactions. Full names of metabolite classes are listed in **Additional file 27.**

Figure 2. LPS biosynthesis and modification in *i*PAO1. (A) VANTED diagram showing the biosynthesis of different LPS molecules. (B) LPS biosynthesis pathway; lipid A and LPS are indicated in the same colour as in (A).

Figure 3. Constitutional genes, reactions and metabolites in *i*PAO1. (A) Sources of *i*PAO1 components. (B) Radar map showing the percentages of metabolites and reactions with valid database identifiers. (C) The COG functional classification of the involved genes in iMO1056, Opt208964 and *i*PAO1. Percentages given in the middle indicate the coverages of COG groups. The proportions of the curated reactions (D), reaction-to-gene ratio (E) and predicted

subcellular localisations of the involved proteins (F) are shown for each pathway or COG group. In panel D, red bars indicate the curated reactions; whereas blue bars indicate the reactions from previous model. In panel D and E, pathways with the highest curation proportion or reaction-to-gene ratio are highlighted in red.

Figure 4. Comparison of the BIOLOG result (left columns) and model prediction (right columns). Blue indicates growth; whereas yellow indicates no growth.

Figure 5. Simulation of the impact of lipid A modifications on bacterial growth, metabolism and OM physiochemical properties. The significant correlation ($P<0.05$) of paired items is indicated in red.

Figure 6. Polymyxin B induced metabolic perturbations. The distributions of metabolic fluxes and metabolite turnover rates are shown in subgraphs with red indicating control and blue indicating polymyxin B treatment.

Figure S1. Sensitivity analysis of the mean metabolic fluxes (A) and metabolite turnover rates to the variation of nutrient uptake upper bounds. Red indicates the control and blue indicates polymyxin B treatment.

Tables and their legends

Table 1. Components in model iMO1056, Opt208964 and *i*PAO1.

		***i*PAO1**	**iMO1056**	**Opt208964**
Genes		1,458	1,042	1,021
Reactions		4,365	992	1,609
	Cytoplasmic metabolic reactions	1,716	730	1,132
	Periplasmic metabolic reactions	403	0	0
	External metabolic reactions	40	0	0
	Transport reactions	960	150	253
	Transport across IM	519	0	0
	Transport across OM	441	0	0
	Transport from cytoplasm to extracellular space	0	150	253
	Boundary reactions	352	112	223
	Reactions without associated genes	628	159	374
	Sink reactions	0	0	1
Metabolites		3,022	858	1,344
	Cytosol	1,519	746	1,121
	Periplasm	698	0	0
	Extracellular space	805	112	223

Pathways	109	-[a]	117

[a] Pathway information is not available in iMO1056 from the Model SEED database.

1027 **Table 2.** Lipid A composition (%) in the outer leaflet of the OM in PAO1 [14].

Lipid A species	Control	Polymyxin B treated
Hexa-lipid A	42.5±0.46	11.7±1.13
Penta-lipid A	57.5±0.46	67.7±3.16
L-Aminoarabinosylated hexa-LA	0	1.24±0.31
L-Aminoarabinosylated penta-LA	0	19.4±3.44
Total	100	100

1028

1029

Table 3. Specific growth rate, significantly altered major exchange fluxes (>1 mmol·gDW^{-1}·h^{-1}), respiration quotient and the fluxes through F_0F_1-ATPase calculated using the RNA-Seq data [51] as flux constraints.

**Exchange flux (mmol·gDW^{-1}·h^{-1})**	**Control**	**Polymyxin B treatment**	***Z*-score**	**FDR[a]**
Specific growth rate (h^{-1})	0.82±0.00	0.67±0.00	10,201.3	0.00
H_2O	46.9±21.8	53.0±19.0	20.37	0.00
O_2	-106.0±23.0	-113.4±19.8	24.30	0.00
CO_2	109.2±22.6	115.8±19.3	22.62	0.00
NH_4^+	36.6±9.29	38.0±8.77	10.94	0.00
Glycine	2.15±4.76	1.92±4.46	3.05	0.00
L-Alanine	1.21±5.01	-0.52±2.20	31.77	0.00
Succinate	2.08±4.19	2.52±4.42	7.27	0.00
H^+	-41.5±14.1	-40.4±11.9	6.44	0.00
Methanethiol	1.53±0.82	1.34±1.11	12.62	0.00
H_2S	1.66±1.74	1.41±2.18	9.29	0.00
Respiration Quotient (RQ)	1.03±0.10	1.02±0.10	7.63	0.00
ATPase (mmol·gDW^{-1}·h^{-1})	-188.6±52.4	-167.6±48.4	29.62	0.00

[a] FDR was calculated using the Benjamini-Hochberg method [105].

Figure 1

Click here to download Figure Figure 1.eps

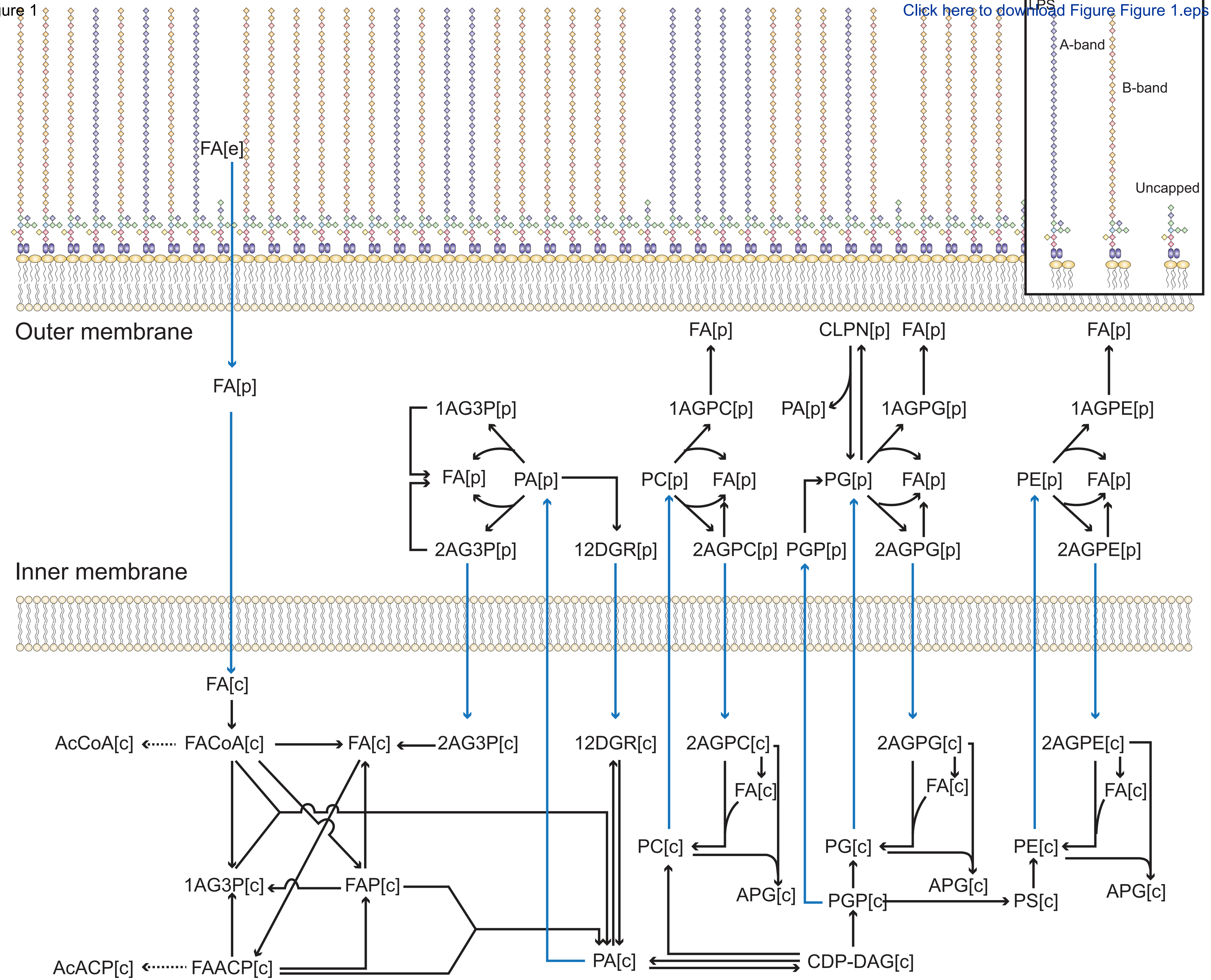

Figure 2

Click here to download Figure Figure 2.eps

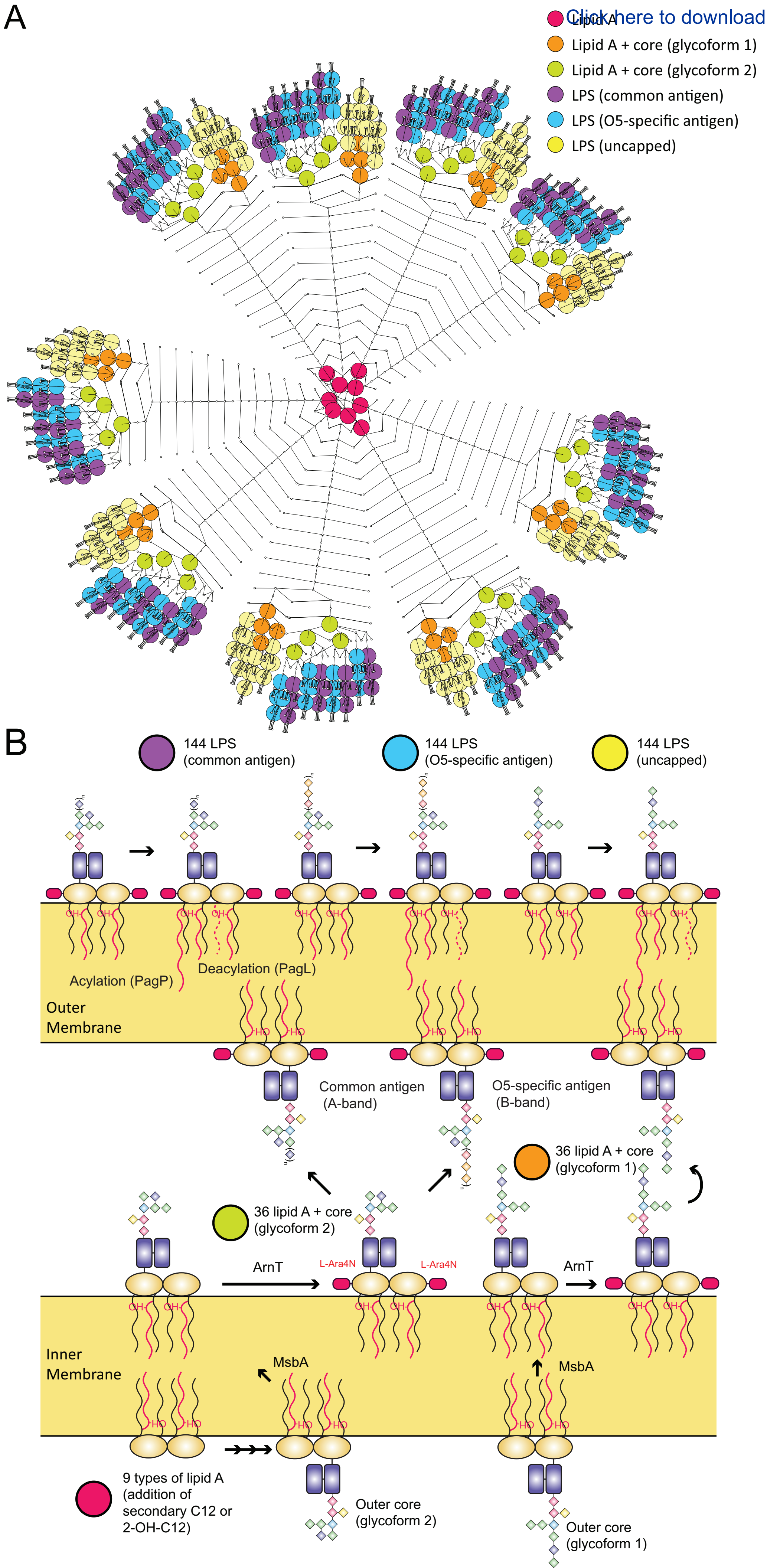

Figure 3A-C

Click here to download Figure Figure 3A-C.eps

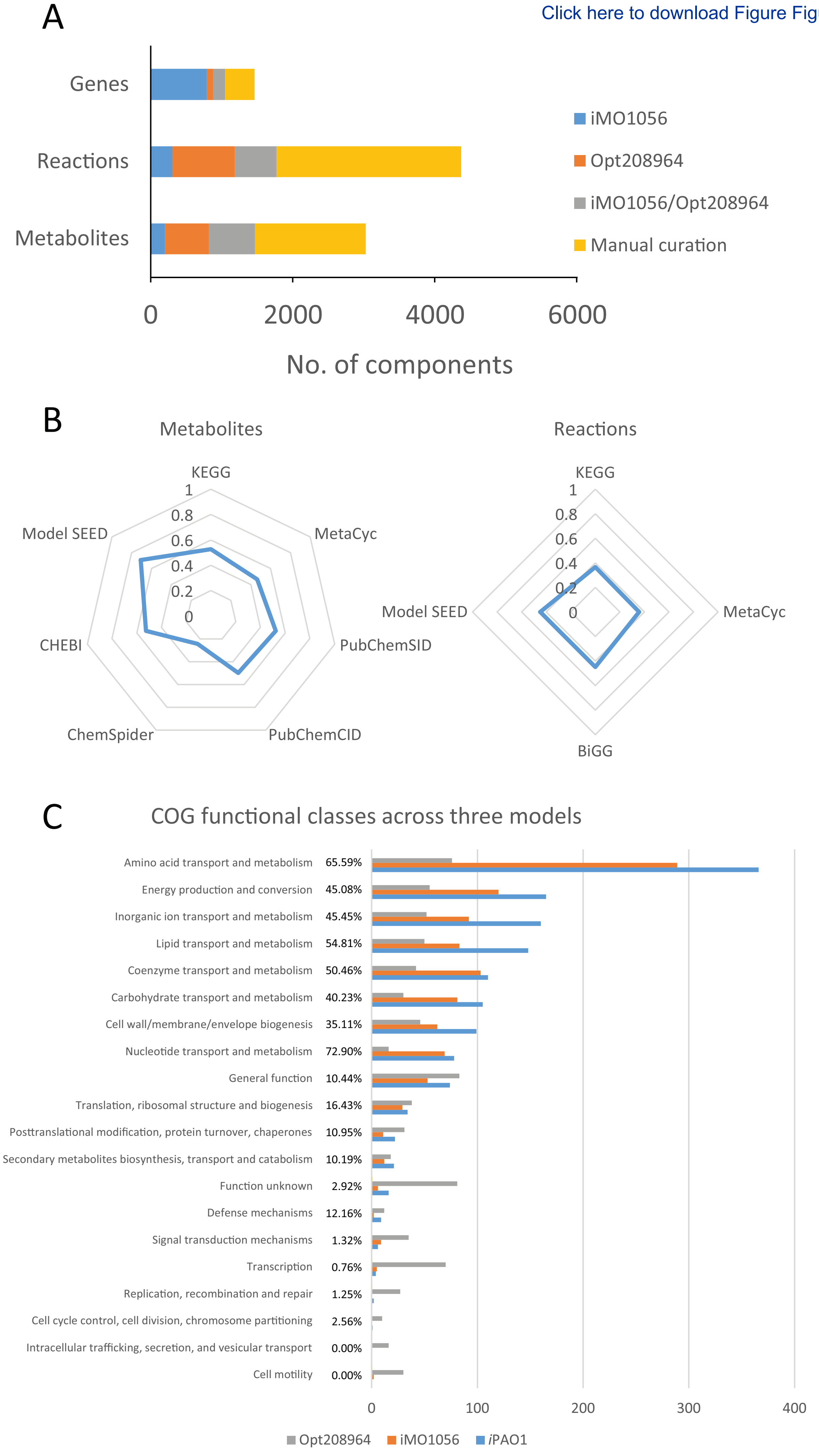

Figure 3D-F

Click here to download Figure Figure 3D-F.eps

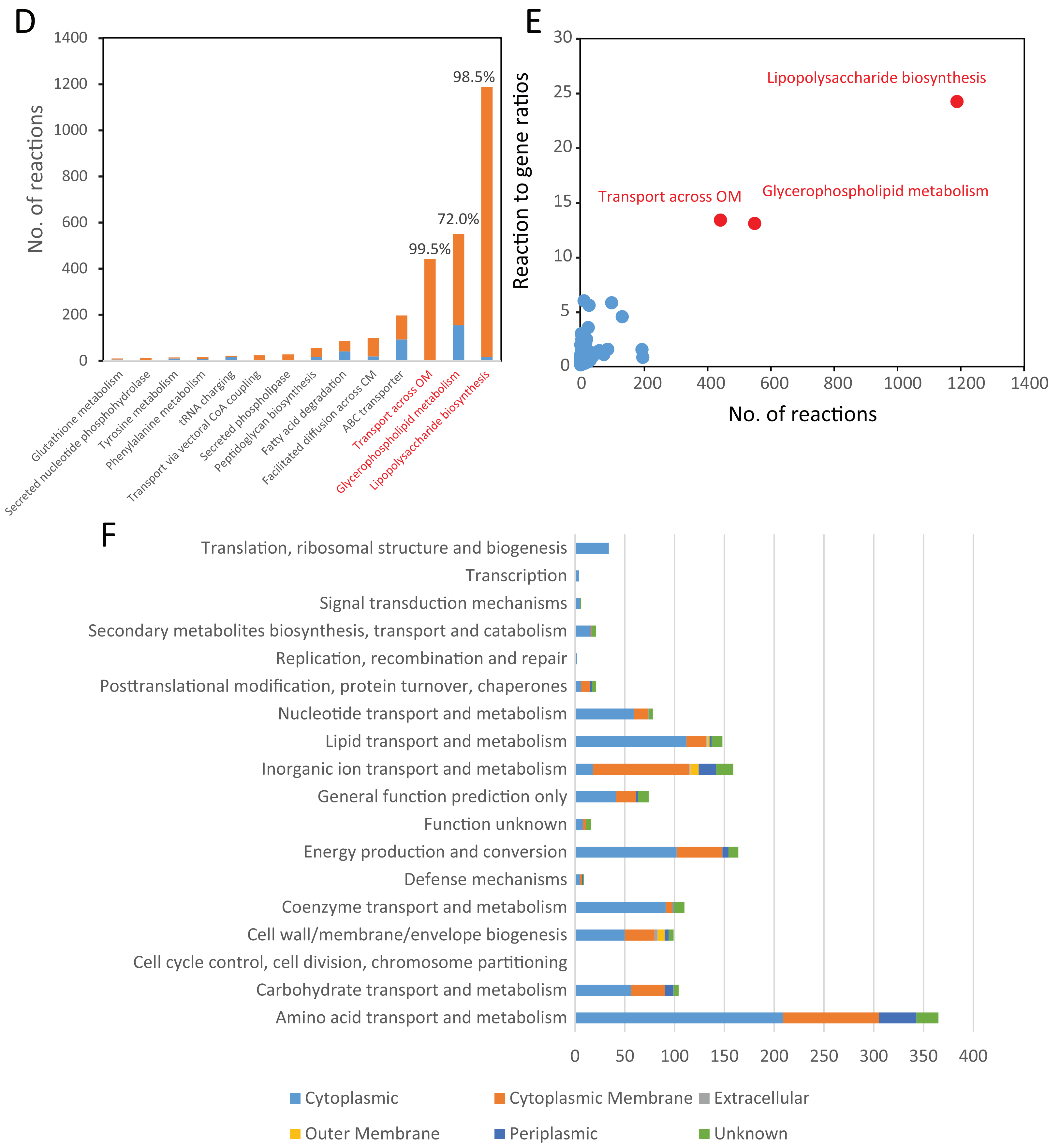

Figure 4

Click here to download Figure Figure 4.eps

PM1 (Carbon)	Biolog	*i*PAO1
L-Arabinose	×	×
N-Acetyl-D-Glucosamine	√	√
D-Saccharic Acid	×	×
Succinic Acid	√	√
D-Galactose	×	×
L-Aspartic Acid	√	√
L-Proline	√	√
D-Alanine	√	√
D-Trehalose	×	√
D-Mannose	×	×
Dulcitol	×	×
D-Serine	×	√
D-Sorbitol	×	×
Glycerol	√	√
L-Fucose	×	×
D-Glucuronic Acid	×	×
D-Gluconic Acid	√	√
D,L-a-Glycerol Phosphate	×	√
D-Xylose	×	×
L-Lactic Acid	√	√
Formic Acid	√	√
D-Mannitol	√	√
L-Glutamic Acid	√	√
D-Glucose-6-Phosphate	×	√
D-Galactonic Acid-g-Lactone	×	×
D,L-Malic Acid	√	√
D-Ribose	√	√
Tween 20	√	×
L-Rhamnose	×	×
D-Fructose	√	√
Acetic Acid	√	√
D-(+)-Glucose	√	√
Maltose	×	×
D-Melibiose	×	×
Thymidine	×	×
L-Asparagine	√	√
D-Aspartic Acid	×	×
D-Glucosaminic Acid	×	×
1,2-Propanediol	√	√
Tween 40	√	×
a-Keto-Gutaric Acid	√	√
a-Ketobutyric Acid	√	√
a-Methyl-D-Galactoside	×	×
a-D-Lactose	×	×
Lactulose	×	×
Sucrose	×	×
Uridine	×	√
L-Glutamine	√	√
m-Tartaric Acid	×	×
D-Glucose-1-Phosphate	×	√
D-Fructose-6-Phosphate	×	×
Tween 80	√	×
a-Hydroxy Glutaric Acid-g-Lactone	×	×
D,L-a-Hydroxy-Butyric Acid	√	√
b-Methyl-D-Glucoside	×	×
Adonitol	×	×
Maltotriose	×	×
2'-Deoxy Adenosine	×	×
Adenosine	√	√
Glycyl-L-Aspartic Acid	×	√
Citric Acid	√	√
m-Inositol	×	×
D-Threonine	×	×
Fumaric Acid	√	√
Bromo Succinic Acid	√	×
Propionic Acid	√	√
Mucic Acid	×	×
Glycolic Acid	×	×
Glyoxylic Acid	×	×
D-Cellobiose	×	×
Inosine	√	√
Glycyl-L-Glutamic Acid	×	√
Tricarballylic Acid	×	×
L-Serine	√	√
L-Threonine	×	√
L-Alanine	√	√
Ala-Gly	×	√
Acetoacetic Acid	√	√
N-Acetyl-D-Mannosamine	×	×
Mono Methyl Succinate	√	√
Methyl Pyruvate	√	×
D-Malic Acid	×	×
L-Malic Acid	√	√
Glycyl-L-Proline	√	√
p-Hydroxy Phenyl Acetic Acid	√	√
M-Hydroxy Phenyl Acetic Acid	×	×
Tyramine	√	√
D-Psicose	×	×
L-Lyxose	×	×
Glucuronamide	×	×
Pyruvic Acid	√	√
L-Galactonic Acid-g-Lactone	×	×
D-Galacturonic Acid	×	×
b-Phenylethylamine	×	×
2-Aminoethanol	√	√

PM2 (Carbon)	Biolog	*i*PAO1
Chondroitin Sulfate C	×	×
a-Cyclodextrin	×	×
b-Cyclodextrin	×	×
g-Cyclodextrin	×	×
Dextrin	×	×
Gelatin	×	×
Glycogen	×	×
Inulin	×	×
Laminarin	×	×
Mannan	×	×
Pectin	×	×
N-Acetyl-D-Galactosamine	×	×
N-Acetyl-Neuraminic Acid	×	×
b-D-Allose	×	×
D-Amygdalin	×	×
D-Arabinose	×	×
D-Arabitol	×	×
L-Arabitol	×	×
Arbutin	×	×
2-Deoxy-D-Ribose	×	×
i-Erythritol	×	×
D-Fucose	×	×
3-0-b-D-Galacto-pyranosyl-D-Arabinose	×	×
Gentiobiose	×	×
L-Glucose	×	×
Lactitol	×	×
D-Melezitose	×	×
Maltitol	×	×
a-Methyl-D-Galactoside	×	×
b-Methyl-D-Galactoside	×	×
3-Methyl Glucose	×	×
b-Methyl-D-Glucuronic Acid	×	×
a-Methyl-D-Mannoside	×	×
b-Methyl-D-Xyloside	×	×
Palatinose	×	×
D-Raffinose	×	×
Salicin	×	×
Sedoheptulosan	×	×
L-Sorbose	×	×
Stachyose	×	×
D-Tagatose	×	×
Turanose	×	×
Xylitol	×	×
N-Acetyl-D-glucosaminitol	×	×
g-Amino Butyric Acid	√	√
d-Amino Valeric Acid	√	√
Butyric Acid	√	√
Capric Acid	×	√
Caproic Acid	√	√
Citraconic Acid	×	×
Citramalic Acid	√	√
D-Glucosamine	×	×
2-Hydroxybenzoic acid	×	×
4-Hydroxy Benzoic Acid Sodium	√	√
b-Hydroxy Butyric Acid	√	√
g-Hydroxy Butyric Acid	×	√
2-Oxovaleric acid	×	×
Itaconic Acid	√	√
5-Keto-D-Gluconic Acid	×	×
D-Lactic Acid Methyl Ester	×	×
Malonic Acid	√	√
Melibionic Acid	×	×
Oxolic Acid	×	×
Oxalomalic Acid	×	×
Quinic Acid	√	√
D-Ribono-1,4-Lactone	×	×
Sebacic Acid	×	×
Sorbic acid	√	√
Succinamic Acid	√	√
D-Tartaric Acid	×	×
L-Tartaric Acid	×	×
Acetamide	×	√
L-Alaninamide	√	√
N-Acetyl-L-Glutamic Acid	√	√
L-Arginine	√	√
Glycine	√	√
L-Histidine	√	√
L-Homoserine	×	√
Hydroxy-L-Proline	√	√
L-Isoleucine	√	√
L-Leucine	√	√
L-Lysine	×	√
L-Methionine	×	√
L-Ornithine	√	√
L-Phenylalanine	×	√
L-Pyroglutamic Acid	√	√
L-Valine	×	√
D,L-Carnitine	√	√
Sec-Butylamine	×	×
D.L-Octopamine	√	√
Putrescine	√	√
Dihydroxy Acetone	×	×
2,3-Butanediol	√	√
Diacetyl	×	×
3-Hydroxy 2-Butanone	×	×

PM3 (Nitrogen)	Biolog	*i*PAO1
Ammonia	√	√
Nitrite	√	√
Sodium Nitrate	√	√
Urea	√	√
Biuret	×	×
L-Alanine	√	√
L-Arginine	√	√
L-Asparagine	√	√
L-Aspartic Acid	√	√
L-Cysteine	√	√
L-Glutamic Acid	√	√
L-Glutamine	√	√
Glycine	√	√
L-Histidine	√	√
L-Isoleucine	√	√
L-Leucine	√	√
L-Lysine	√	√
L-Methionine	√	√
L-Phenylalanine	√	√
L-Proline	√	√
L-Serine	√	√
L-Threonine	√	√
L-Tryptophan	√	√
L-Tyrosine	√	√
L-Valine	√	√
D-Alanine	√	√
D-Asparagine	√	√
D-Aspartic Acid	×	×
D-Glutamic Acid	√	√
D-Lysine	√	√
D-Serine	√	√
D-Valine	√	√
L-Citrulline	√	√
L-Homoserine	×	√
L-Ornithine	√	√
N-Acetyl-L-Glutamic Acid	√	√
N-Phthaloyl-L-Glutamic Acid	√	×
L-Pyroglutamic Acid	√	√
Hydroxylamine	×	√
Methylamine	×	×
N-Amylamine	×	×
N-Butylamine	×	×
Ethylamine	×	×
Ethanolamine	√	×
Ethylenediamine	×	×
Putrescine	√	√
Agmatine	√	√
Histamine	√	√
b-Phenylethylamine	√	√
Tyramine	√	√
Acetamide	√	√
Formamide	×	×
Glucuronamide	√	√
D,L-Lactamide	√	√
D-Glucosamine	×	×
D-Galactosamine	×	×
D-Mannosamine	×	×
N-Acetyl-D-Glucosamine	√	√
N-Acetyl-D-Galactosamine	√	√
N-Acetyl-D-Mannosamine	×	×
Adenine	√	√
Adenosine	√	√
Cytidine	√	√
Cytosine	√	√
Guanine	√	√
Guanosine	√	√
Thymine	√	√
Thymidine	×	×
Uracil	√	√
Uridine	√	√
Inosine	√	√
Xanthine	√	√
Xanthosine	√	√
Uric Acid	√	√
Alloxan	√	×
Allantoin	√	√
Parabanic Acid	√	×
D,L-a-Amino-N-Butyric Acid	×	×
g-Amino Butyric Acid	√	√
e-Amino-N-Caproic Acid	×	×
D,L-a-Amino- Caprylic Acid	×	×
d-Amino-N-Valeric Acid	√	√
a-Amino-N-Valeric Acid	√	×
Ala-Asp	√	√
Ala-Gln	√	√
Ala-Glu	√	√
Ala-Gly	√	√
Ala-His	√	√
Ala-Leu	√	√
Ala-Thr	√	√
Gly-Asn	√	√
Gly-Gln	√	√
Gly-Glu	√	√
Gly-Met	×	√
Met-Ala	√	√

Figure 5

Click here to download Figure Figure 5.pdf

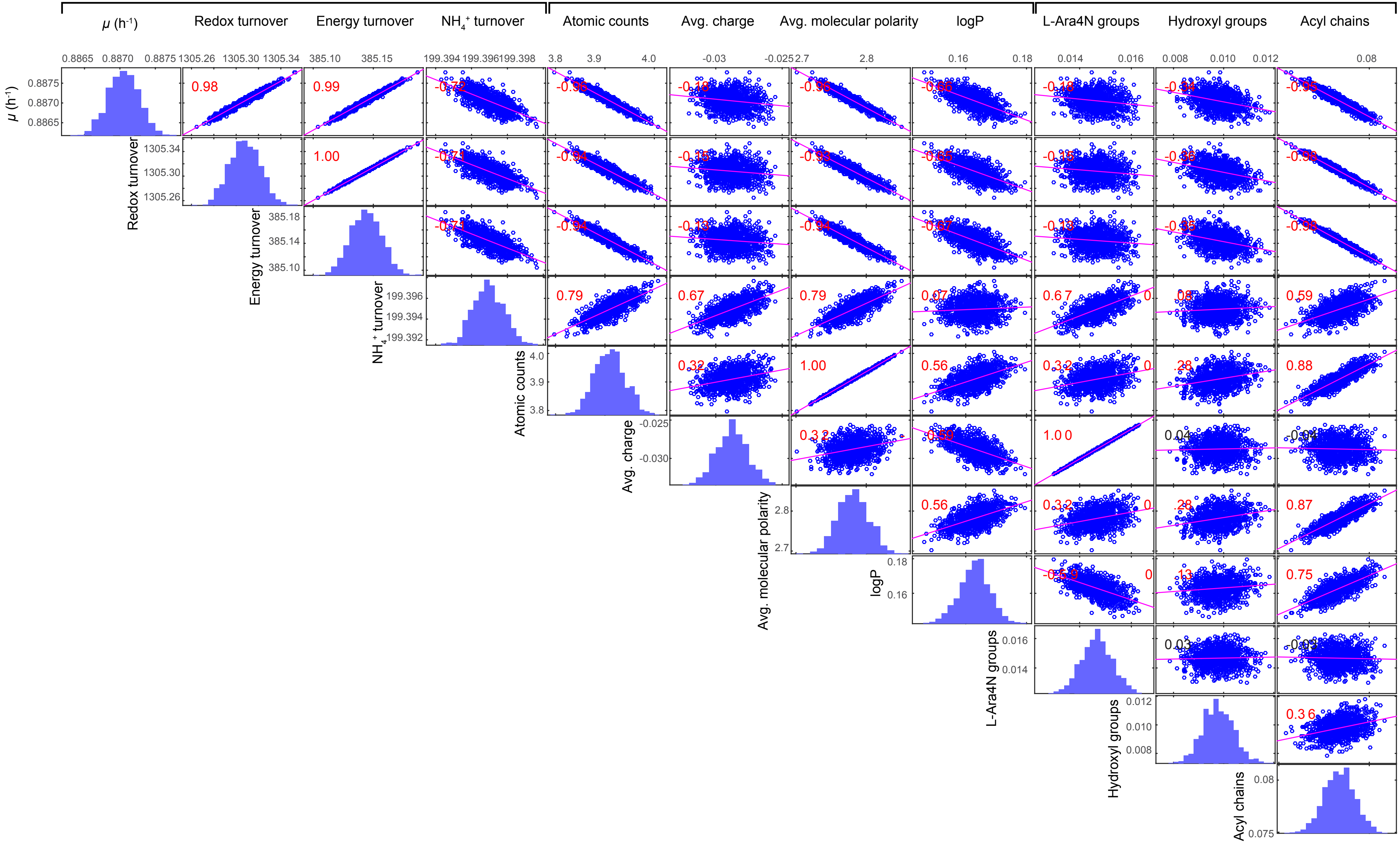

Figure 6

Click here to download Figure Figure 6.eps

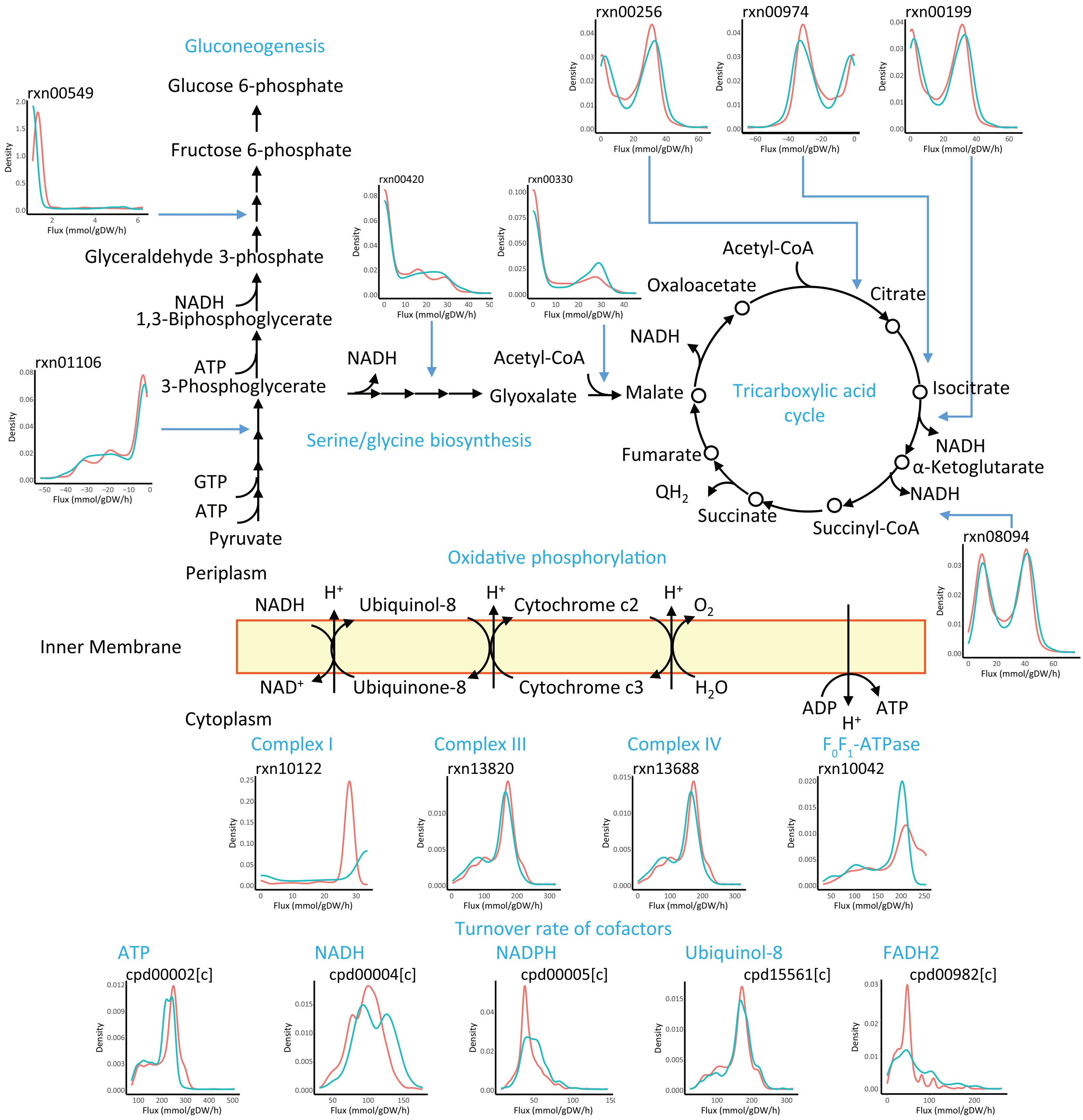

Figure S1

Click here to download Figure Figure S1.eps

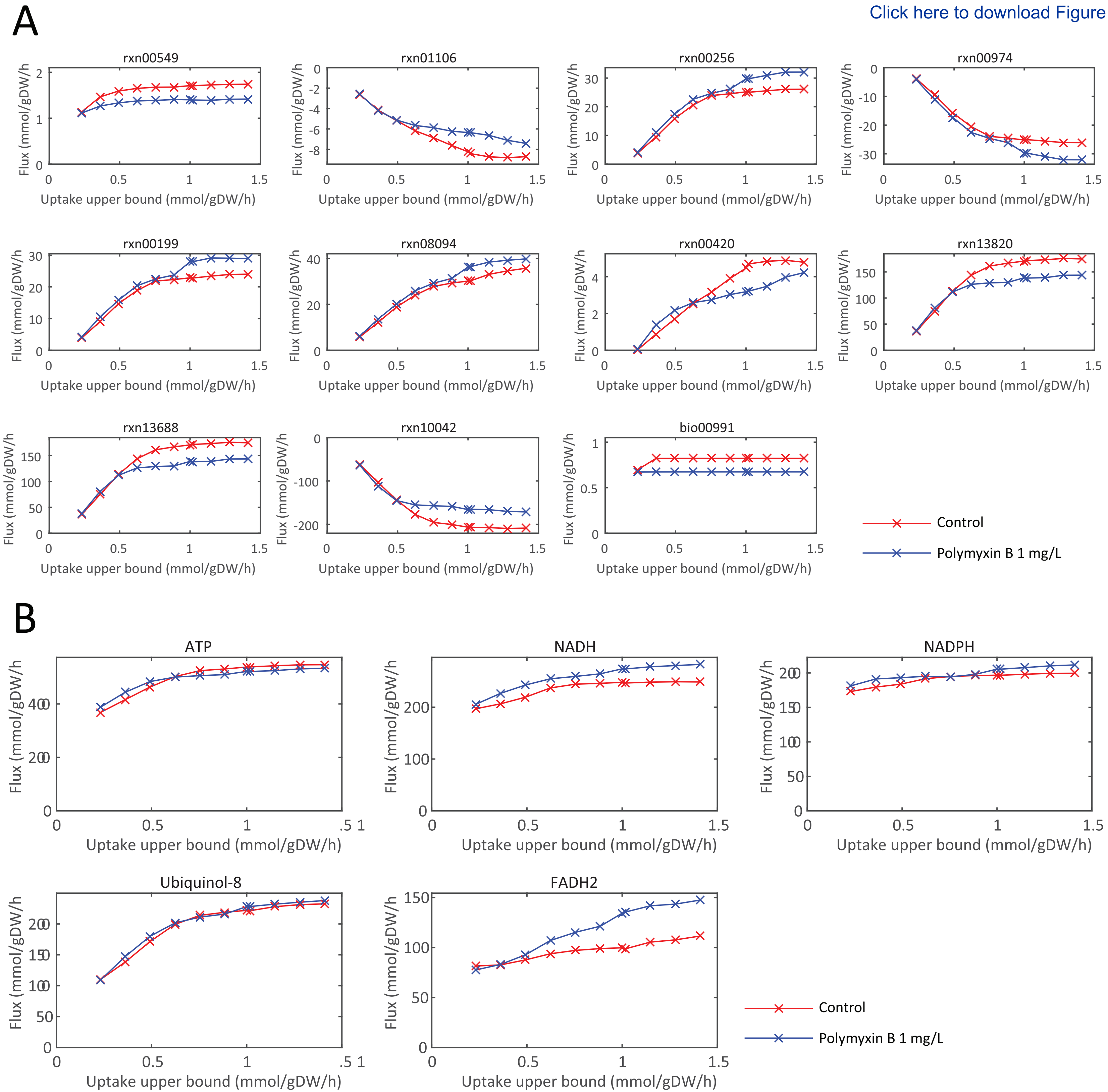

Additional file 1

Click here to access/download
Supplementary Material
additionalFile1.docx

Additional file 2

Click here to access/download
Supplementary Material
additionalFile2.xlsx

Additional file 3

Click here to access/download
Supplementary Material
additionalFile3.xlsx

Additional file 4

Click here to access/download
Supplementary Material
additionalFile4.xlsx

Additional file 5

Click here to access/download
Supplementary Material
additionalFile5.xlsx

Additional file 6

Click here to access/download
Supplementary Material
additionalFile6.xlsx

Additional file 7

Click here to access/download
Supplementary Material
additionalFile7.xlsx

Additional file 8

Click here to access/download

Supplementary Material

additionalFile8.xlsx

Additional file 9

Click here to access/download
Supplementary Material
additionalFile9.xlsx

Additional file 10

Click here to access/download
Supplementary Material
additionalFile10.xlsx

Additional file 11

Click here to access/download
Supplementary Material
additionalFile11.xlsx

Additional file 12

Click here to access/download
Supplementary Material
additionalFile12.xlsx

Additional file 13

Click here to access/download
Supplementary Material
additionalFile13.xlsx

Additional file 14

Click here to access/download
Supplementary Material
additionalFile14.xlsx

Additional file 15

Click here to access/download

Supplementary Material

additionalFile15.xlsx

Additional file 16

Click here to access/download
Supplementary Material
additionalFile16.xlsx

Additional file 17

Click here to access/download
Supplementary Material
additionalFile17.xlsx

Additional file 18

Click here to access/download

Supplementary Material

additionalFile18.xlsx

Additional file 19

Click here to access/download
Supplementary Material
additionalFile19.xlsx

Additional file 20

Click here to access/download
Supplementary Material
additionalFile20.xlsx

Additional file 21

Click here to access/download
Supplementary Material
additionalFile21.xlsx

Additional file 22

Click here to access/download
Supplementary Material
additionalFile22.xlsx

Additional file 23

Click here to access/download
Supplementary Material
additionalFile23.xlsx

Additional file 24

Click here to access/download
Supplementary Material
additionalFile24.xlsx

Additional file 25

Click here to access/download
Supplementary Material
additionalFile25.xlsx

Additional file 26

Click here to access/download
Supplementary Material
additionalFile26.xlsx

Additional file 27

Click here to access/download
Supplementary Material
additionalFile27.xlsx

MONASH University

Professor Jian Li
Head, Laboratory of Antimicrobial Systems Pharmacology
Monash Biomedicine Discovery Institute

Dr Laurie Goodman
Editor-in-Chief
GigaScience

16 October 2017

Dear Dr Goodman,

We are pleased to submit our manuscript entitled "*Genome-scale metabolic modelling of responses to polymyxins in Pseudomonas aeruginosa*" for your consideration as an original Research Article in *GigaScience*.

Antimicrobial resistance has become one of the greatest threats to global health today. Multidrug-resistant (MDR) *P. aeruginosa* has been categorised by the World Health Organization as a "Critical" Gram-negative 'superbug' against which no new antibiotics will be available in the near future. Polymyxins are 'old' antibiotics firstly discovered in 1947, but have been abandoned since the 1970s. Over the last decade polymyxins have been revived as the last-line therapy against Gram-negative 'superbugs', including *P. aeruginosa*, which are resistant to all other antibiotics. However, the mechanism of their antibacterial activity remains largely unknown.

Here we report the construction of a superior genome-scale metabolic model (GSMM) *i*PAO1 for *P. aeruginosa* PAO1 which represents *the largest genome-scale metabolic model thus far for any Gram-negative bacteria*. *i*PAO1 provides a powerful systems pharmacology tool to elucidate the complex mode of action of antibiotics and shift the paradigm of the "one-gene, one-receptor, one-mechanism" approach. It is able to quantitatively simulate complex bacterial cellular responses in response to antibiotic treatments.

To date, there are four curated GSMMs for *P. aeruginosa*, iMO1056 (developed in 2008), Opt20896429 (2010), iMO1086 and iPae1146 (two minor updated versions of iMO1056 developed in 2011 and 2017, respectively), and all are for the strain PAO1. *Unfortunately, none of these four GSMMs incorporates the periplasmic space, and glycerolphosholipid (GPL) and lipopolysaccharide (LPS) biosynthesis is very poorly represented.* These shortcomings significantly limit their usefulness for antimicrobial pharmacology. Growth prediction with *i*PAO1 on 190 carbon and 95 nitrogen nutrients outperformed all the previous models with an accuracy of 89.1%. Prediction of the essential genes for growth on rich media achieved a high accuracy of 87.9%. Specifically, the significant advantages of our *i*PAO1 include: (1) incorporation of the periplasmic space; (2) addition of detailed GPL and LPS biosynthesis pathways supported by our own metabolomics and lipidomics data; and (3) significant expansion of the modelling scale with a high prediction accuracy. For the first time, metabolic simulation using *i*PAO1 showed that lipid A modifications exert limited impacts on bacterial growth and metabolism, but remarkably change the physiochemical properties of bacterial outer membrane. Modelling with transcriptomics constraints revealed a broad range of metabolic responses to polymyxin treatment, including reduced biomass formation, upregulated amino acids catabolism, induced tricarboxylic acid cycle, and increased redox turnover. Overall,

Department of Microbiology
19 Innovation Walk
Monash University
VIC 3800, Australia
Telephone: (+61 3) 990 39702 Facsimile: (+61 3) 990 29222 Email: Jian.Li@monash.edu
Web: www.monash.edu/pharm/research/areas/drug-delivery/labs/li-lab

Unintended recipient: please notify as soon as possible and destroy all pages received

MONASH University

Professor Jian Li
Head, Laboratory of Antimicrobial Systems Pharmacology
Monash Biomedicine Discovery Institute

our GSMM approach has a significant potential in accelerating antimicrobial pharmacological discovery against Gram-negative ‘superbugs’.

To the best of our knowledge, this study is the first to integrate antimicrobial pharmacology, computational biology, metabolic network and systems pharmacology to analyse large-scale datasets, in order to better understand the dynamic and complex nature of polymyxin killing and resistance. We believe this manuscript perfectly matches the theme of *GigaScience* and will be of broad interest to microbiologists, bioinformaticians and antimicrobial researchers.

We confirm that our submission comprises original and unpublished material which is not currently under consideration for publication elsewhere, and has been approved by all authors. Thank you for considering our work for publication in *GigaScience*. We look forward to your correspondence.

Yours sincerely,

Jian Li PhD

Falk Schreiber PhD

Department of Microbiology
19 Innovation Walk
Monash University
VIC 3800, Australia
Telephone: (+61 3) 990 39702 Facsimile: (+61 3) 990 29222 Email: Jian.Li@monash.edu
Web: www.monash.edu/pharm/research/areas/drug-delivery/labs/li-lab

Unintended recipient: please notify as soon as possible and destroy all pages received
