# Supplementary material for: Genome-scale metabolic modeling of responses to polymyxins in Pseudomonas aeruginosa
Source: Gigascience. 2018 Mar 13;7(4):giy021. doi: 10.1093/gigascience/giy021 (PMC6333913; doi:10.1093/gigascience/giy021)
Supplement: Supplemental material [file giy021_supp.zip › additionalFile1.docx]

**Additional file 1**

**Supplementary Methods**

**Manual curation of glycerophospholipid biosynthesis pathway.** GPLs are key components of bacterial IM and OM. Alterations of the compositions of GPLs in bacterial membranes due to antibiotic treatments have been demonstrated [1, 2]. Previous research [3] and our lipidomics studies [4, 5] showed diverse GPL species in *P. aeruginosa*, which include 1,2-diacyl-*sn*-glycerol 3-phosphate (PA), phosphatidylethanolamine (PE), phosphatidylcholine (PC), phosphatidylglycerol (PG), phosphatidylglycerophosphate (PGP), phosphatidylserine (PS) and cardiolipin (CLPN); and the intermediates 1- or 2-acyl-*sn*-glycerol-phosphate (lysoPA), 1- or 2-acyl-*sn*-glycero-3-phosphoethanolamine (lysoPE), 1- or 2-acyl-*sn*-glycero-3-phosphocholine (lysoPC), 1- or 2-acyl-*sn*-glycero-3-phosphoglycerol (lysoPG) and CDP-diacylglycerol (CDP-DG). Detailed biosynthesis and interconversion reactions of GPL species were added for each possible acyl chain including saturated (C12:0, C14:0, C16:0 and C18:0), unsaturated (C14:1, C16:1 and C18:1), and branched structures (iso-C14:0, iso-C15:0, anteiso-C15:0, iso-C16:0, iso-C17:0 and anteico-C17:0) (**Fig 1, Additional files 13 and 14**). Overall, 386 unique metabolites (i.e. 66.2% of the 583 metabolites in the GPL metabolism pathway) and 367 reactions (66.7% of the 550 reactions in the GPL metabolism pathway) were incorporated.

**Manual curation of lipopolysaccharide biosynthesis and modification pathways.** Specifically, the biosynthesis of the lipid A backbone starts from acylation of UDP-GlcNAc (LpxA, PA3644), followed by formation of the lipid A disaccharide and incorporation of keto-deoxyoctulosonate (KDO) residues [6]. Modifications to lipid A in *P. aeruginosa* include (1) secondary palmitoylation of the GlcN^II^ (PagP, PA1343); (2) deacylation of the primary 10:0 (3-OH) group at position 3 of GlcN^I^ (PagL, PA4661); (3) non-stoichiometric addition of 4-amino-4-deoxy-L-arabinose (L-Ara4N) to the terminal phosphate groups at GlcN^I^ and GlcN^II^ (ArnT, PA3556); (4) non-stoichiometric 2-hydroxylation of the secondary lauroyl group acyl chains (LpxO1 and LpxO2, encoded by PA4512 and PA0936, respectively); and (5) non-stoichiometric addition of the secondary lauroyl group (LpxL1 and LpxL2, encoded by PA3242 and PA0011, respectively) [7].

There are two types of outer-core glycoforms in *P. aeruginosa* [8], of which only glycoform 2 binds to *O*-antigens. There are two types of *O*-antigens in PAO1, the common antigen and O5 antigen [7]. Both the nascent LPS core (inner core plus outer core) and *O*-antigen precursors are synthesised within the cytoplasm, flipped to periplasm through the trans-membrane transporters MsbA (PA4997) and Wzx (PA3153), respectively. *O*-antigen oligosaccharides are polymerised on the periplasmic surface of the inner membrane by Wzy (PA3154) and then transferred to nascent core-lipid A by WaaL (PA4999). The newly formed LPS is then incorporated into the outer surface of OM via OstA and LptE (PA0595 and PA3988) [9]. The detailed representation of LPS biosynthesis and modifications is in **Fig 2**.

**Supplementary Results**

**Sensitivity analysis of nutrient uptake bounds**

Sensitivity analysis was conducted to assess the impact of nutrient uptake bounds to bacterial growth and metabolism in CAMHB media. Within 0.9-1.4 mmol⋅gDW^-1^⋅h^-1^, the fluxes via gluconeogenesis (rxn00549 and rxn01106), serine biosynthesis (rxn00420), electron transport chain (rxn13820 and rxn13688), ATPase (rxn10042) and biomass formation (bio00991) distributions were consistently downregulated upon polymyxin B treatment. The fluxes through TCA cycle (rxn00256, rxn00974, rxn00199 and rxn08094) were upregulated. The turnover of major redox equivalents (e.g. ATP, NADH, NADPH, ubiquinol-8, FADH2) was upregulated, while the ATP turnover was downregulated (**Fig S1**). Further reducing uptake upper bounds from 0.9 to 0.23 mmol⋅gDW^-1^⋅h^-1^ caused remarkable impacts on most of the fluxes and metabolite turnovers (**Fig S1**). Taken together, constraining the uptake of CaMHB ingredients to 1 mmol⋅gDW^-1^⋅h^-1^ allows *i*PAO1 to make robust predictions of metabolic fluxes.

**References**

1. Dalebroux ZD, Matamouros S, Whittington D, Bishop RE and Miller SI. PhoPQ regulates acidic glycerophospholipid content of the *Salmonella Typhimurium* outer membrane. Proc Natl Acad Sci U S A. 2014;111:1963-8.

2. Cox E, Michalak A, Pagentine S, Seaton P and Pokorny A. Lysylated phospholipids stabilize models of bacterial lipid bilayers and protect against antimicrobial peptides. Biochim Biophys Acta. 2014;1838:2198-204.

3. Kondakova T, D'Heygere F, Feuilloley MJ, Orange N, Heipieper HJ and Duclairoir Poc C. Glycerophospholipid synthesis and functions in *Pseudomonas*. Chem Phys Lipids. 2015;190:27-42.

4. Han M, Zhu Y, Cheah SE, Johnson MD, Yu H, Shen HH, et al. Polymyxin resistance in *Pseudomonas aeruginosa*: metabolomic changes underpin lipid A modifications. In: *ASM Microbe 2017* Boston, USA, 2016, p.491.

5. Han ML, Zhu Y, Cheah S-E, Johnson MD, Yu HH, Maifiah MHM, et al. Polymyxin resistance due to mutations in *pmrB* caused global metabolomics changes in *Pseudomonas aeruginosa*. In: *The Australian & New Zealand Metabolomics Conference* Melbourne, Australia, 30 March 2016, p.106.

6. Raetz CR, Reynolds CM, Trent MS and Bishop RE. Lipid A modification systems in Gram-negative bacteria. Annu Rev Biochem. 2007;76:295-329.

7. Lam JS, Taylor VL, Islam ST, Hao Y and Kocincova D. Genetic and functional diversity of *Pseudomonas aeruginosa* lipopolysaccharide. Front Microbiol. 2011;2:118.

8. Pier GB. *Pseudomonas aeruginosa* lipopolysaccharide: a major virulence factor, initiator of inflammation and target for effective immunity. Int J Med Microbiol. 2007;297:277-95.

9. Werneburg M, Zerbe K, Juhas M, Bigler L, Stalder U, Kaech A, et al. Inhibition of lipopolysaccharide transport to the outer membrane in *Pseudomonas aeruginosa* by peptidomimetic antibiotics. Chembiochem. 2012;13:1767-75.
