# Supplementary material for: Genome-scale metabolic modeling of responses to polymyxins in Pseudomonas aeruginosa
Source: Gigascience. 2018 Mar 13;7(4):giy021. doi: 10.1093/gigascience/giy021 (PMC6333913; doi:10.1093/gigascience/giy021)
Supplement: Response_to_Reviewer_Comments_Original_Submission.pdf [file giy021_response_to_reviewer_comments_original_submission.pdf]

Author's Response To Reviewer Comments

Dr Laurie Goodman
Editor-in-Chief
GigaScience

Dear Dr Goodman,
RE: Manuscript ID: GIGA-D-17-00272: “Genome-scale metabolic modelling of responses to polymyxins in Pseudomonas aeruginosa"

Thank you for the opportunity to revise our manuscript. Please find below a point-by-point response to the reviewers’ comments. All major changes have been highlighted in yellow in the ‘marked’ version of the revised manuscript.

The raw data have been submitted to Sequence Read Archive (SRA) and MetaboLights databases and will be made publicly available hopefully by 31 Jan, 2018.
We look forward to your correspondence and thank you very much.

Best regards,

Jian Li
Falk Schreiber

Monash Biomedicine Discovery Institute, Department of Microbiology, Faculty of Medicine, Nursing and Health Sciences, Monash University, Melbourne 3800, Australia

Department of Computer and Information Science, University of Konstanz, Konstanz 78457, Germany

Reviewer #1:

1. Introduction section. I would specify that the specific approach of applying gene expression constraints to obtain condition-specific GEMs have been previously used for other MDR bacteria (e.g. A. baumannii doi:10.1038/s41598-017-03416-2).

Response: The manuscript has been revised accordingly (Pages 3-4, Lines 74-77).

2. Lines 217-218: I would remove the sentence "Therefore, iPAO1 is a well-defined, metabolism-dedicated model.", in that it is included in the definition of "metabolic reconstruction". The presence of genes associated to non-metabolic COG categories is, in my opinion, due to the presence of misannotated (for what concerns the COG categories) genes. Honestly, I wouldn't use the distribution of such categories as a measure of a model goodness, especially considering that some genes can be associated to multiple categories. All the other comparisons the authors made already highlighted how this reconstruction is the best one.

Response: The sentence was removed in the revised manuscript as suggested (Page 9, Line 221).

3. Lines 253-258: "this is possibly due to the incorporation of new genes (30.5% increase compared to Opt208964; 27.2% increase compared to iPae1146) whose metabolic functions were previously misannotated." This is not clear... do the authors mean that the addition of new genes brought alternative routes to bypass previously essential gene deletion? This should be rephrased, and, if possible, the proposed explanation should be tested.

Response: The sentence was rephrased as suggested. In the revised manuscript an example was provided to delineate that incorporating isozymes altered previous essentiality prediction results. Please refer Page 11, Lines 256-264 in the revised manuscript.

4. Section "Elucidating the mechanisms of metabolic responses to polymyxin treatment":
In this section the authors use the previously presented model to describe the changes at a systems level of the metabolism in presence of polymyxin treatment. I have two issues concerning this section: The way the authors computed the flux distribution in presence of antibiotics. Given the non-optimal state of such condition, I feel that MOMA is more appropriate. I suggest the authors to test this and compare the results with the current ones.

Response: We respectfully disagree with the reviewer. Minimisation Of Metabolic Adjustment (MOMA) was developed to predict the metabolic flux redistributions in gene knockout mutants. MOMA hypothesises that metabolism of the mutant tends to approximate the wild-type (Segre et al., 2002, Proc Natl Acad Sci. 99(23):15112-7), which is distinct from the antibiotic treatment scenario. For instance, our metabolomics data have demonstrated that polymyxin treatment caused dramatic metabolic changes in bacteria (e.g. Maifiah et al., 2017, Sci Rep, 7: 45527). Therefore, metabolic fluxes with and without antibiotic treatment should not be calculated with MOMA, but FBA (see e.g. Colijn et al., 2009, PLoS Comput Biol, 5(8): e10004). Please refer Pages 17, Lines 414-420 in our revised manuscript.

5. Although a description of the systemic changes induced by antibiotics is important, I think that the authors are missing an important point, that is the condition-specific essential genes. In my opinion this is very important and interesting, also considering that a selling point of the manuscript is that "iPAO1 offers an in silico platform for precision antimicrobial pharmacology therapy".

Response: We appreciate reviewer’s suggestion. The methods and results on the condition-specific essential genes were included in the revised manuscript (Page 23, Lines 552-555; Page 11, Lines 264-269).

Reviewer #2:
1. On page 13 line 260 in the section on lipid A modification the authors mention changes in fluxes. They state that fluxes were calculated using FBA. However, in the Methods section I see that the authors used sampling to explore the solution space. The authors must use sampling to compare fluxes between conditions. If the author's used sampling here to the authors must specify so in the main text.

Response: We employed sampling in our original study and have specified the sampling methods in the revised manuscript as suggested. Please refer Page 12, Lines 274, 286-287.

2. Page 14 line 286 - the authors must state how the RNAseq was used to constrain the model. They mention it in the Discussion section (E-FLUX method). However, this must be stated in the Results section as well.

Response: E-Flux method was employed to constrain the model with the RNAseq data, which has been specified in the Methods (Pages 24-25, Lines 586-592) and Results sections as suggested (Page 13, Line 302).

3. Page 14 line 295 - I have a major question about how the authors simulate for growth in CAMHB media? This is an undefined media type and in the Methods section they describe that they set the uptake rates to 1 mmol*gDW*hr^-1 for major carbon sources. The authors must explain why this uptake rate is justified. Did the authors perform any sensitivity analysis on these uptake rates? It's very reasonable to assume that changes in these rates would dramatically affect the fluxes described by the authors in this section. Some justification and or sensitivity analysis must be added here to explain the validity of these uptake rates for growth in this condition.

Response: Previous measurements showed that P. aeruginosa cells uptake amino acids at a rate ranging from 0.26 to 1.44 mmol·gDW^-1·h^-1 (J Bacteriol, 105(3): 1039-46; J Bacteriol, 152(2): 636-42). The import of CAMHB ingredients was thus constrained to 1 mmol·gDW^-1·h^-1 without loss of generality. Sensitivity analysis was conducted as suggested (Methods section, Page 25, Lines 592-597 and 600-602) and the results showed that the changes in nutrient uptake bounds did not dramatically affect the key metabolic fluxes. Our sensitivity analysis results have been provided in the Results section (Page 13, Lines 304-305), Additional File 1 and Figure S1.

4. Page 15 line 303 - the authors must state what the "control" condition is. Is this compared to PAO1 growing in CAMHB without polymyxin treatment? Or compared to growth on a different media type, i.e. M9 minimal media + glucose?

Response: The control condition was specified in the revised manuscript as suggested (Page 13 Line 321).

5. The authors state that their model is "the most comprehensive for a gram-negative organism to date". On what basis is this claim made? We would recommend tempering this statement or perhaps limiting it to Pseudomonas models.

Response: The statement was limited to Pseudomonas and was modified in the revised manuscript (Page 2, Line 46; Page 4, Line 98; Page 20, Line 472).
