# Supplementary material for: Genome-scale metabolic modeling of responses to polymyxins in Pseudomonas aeruginosa
Source: Gigascience. 2018 Mar 13;7(4):giy021. doi: 10.1093/gigascience/giy021 (PMC6333913; doi:10.1093/gigascience/giy021)
Supplement: Reviewer_1_Report_(Original_Submission) -- Emanuele Bosi — 28 Nov 2017 Reviewed [file giy021_reviewer_1_report_(original_submission).pdf]

Reviewer Report

Title: Genome-scale metabolic modelling of responses to polymyxins in Pseudomonas aeruginosa

Version: Original Submission **Date:** 28 Nov 2017

Reviewer name: Emanuele Bosi

Reviewer Comments to Author:

In this work Zhu et colleagues release a high-quality metabolic reconstruction for the bacterium P. aeruginosa PAO1. The model has been validated and used to achieve an understanding of the adaptation to antibiotics.
Given the global threat posed by MDR bacteria, I sincerely think systematic approaches (as this one) to identify potential weak points in troublesome strains are to be encouraged.
Overall, the manuscript is well written and the conclusions are supported by the amount of data reported, and therefore should be published after some modifications. Below are reported my specific comments, divided for each section.

Introduction:

1) I would specify that the specific approach of applying gene expression constraints to obtain condition-specific GEMs have been previously used for other MDR bacteria (e.g. A. baumannii doi:10.1038/s41598-017-03416-2).

Analyses:

1) lines 217-218: I would remove the sentence "Therefore, iPAO1 is a well-defined, metabolism-dedicated model.", in that it is included in the definition of "metabolic reconstruction". The presence of genes associated to non-metabolic COG categories is, in my opinion, due to the presence of misannotated (for what concerns the COG categories) genes. Honestly, I wouldn't use the distribution of such categories as a measure of a model goodness, especially considering that some genes can be associated to multiple categories. All the other comparisons the authors made already highlighted how this reconstruction is the best one.

2) lines 253-258: "this is possibly due to the incorporation of new genes (30.5% increase compared to Opt208964; 27.2% increase compared to iPae1146) whose metabolic functions were previously misannotated."
This is not clear... do the authors mean that the addition of new genes brought alternative routes to bypass previously essential gene deletion? This should be rephrased, and, if possible, the proposed explanation should be tested.

3) section "Elucidating the mechanisms of metabolic responses to polymyxin treatment":

In this section the authors use the previously presented model to describe the changes at a systems level of the metabolism in presence of polymixin treatment.
I have two issues concerning this section:
3.1) The way the authors computed the flux distribution in presence of antibiotics. Given the non-optimal state of such condition, I feel that MOMA is more appropriate. I suggest the authors to test this and compare the results with the current ones.
3.2) Although a description of the systemic changes induced by antibiotics is important, I think that the authors are missing an important point, that is the condition-specific essential genes. In my opinion this is very important and interesting, also considering that a selling point of the manuscript is that "iPAO1 offers an in silico platform for precision antimicrobial pharmacology therapy"

Methods

Are the methods appropriate to the aims of the study, are they well described, and are necessary controls included? Yes

Conclusions

Are the conclusions adequately supported by the data shown? Yes

Reporting Standards

Does the manuscript adhere to the journal's guidelines on minimum standards of reporting? Yes

Statistics

Are you able to assess all statistics in the manuscript, including the appropriateness of statistical tests used? Yes, and I have assessed the statistics in my report.

Quality of Written English

Please indicate the quality of language in the manuscript: Acceptable

Declaration of Competing Interests

Please complete a declaration of competing interests, considering the following questions:

- Have you in the past five years received reimbursements, fees, funding, or salary from an organisation that may in any way gain or lose financially from the publication of this manuscript, either now or in the future?
- Do you hold any stocks or shares in an organisation that may in any way gain or lose financially from the publication of this manuscript, either now or in the future?

- Do you hold or are you currently applying for any patents relating to the content of the manuscript?
- Have you received reimbursements, fees, funding, or salary from an organization that holds or has applied for patents relating to the content of the manuscript?
- Do you have any other financial competing interests?
- Do you have any non-financial competing interests in relation to this paper?

If you can answer no to all of the above, write 'I declare that I have no competing interests' below. If your reply is yes to any, please give details below.

I declare that I have no competing interests

I agree to the open peer review policy of the journal. I understand that my name will be included on my report to the authors and, if the manuscript is accepted for publication, my named report including any attachments I upload will be posted on the website along with the authors' responses. I agree for my report to be made available under an Open Access Creative Commons CC-BY license (http://creativecommons.org/licenses/by/4.0/). I understand that any comments which I do not wish to be included in my named report can be included as confidential comments to the editors, which will not be published.

I agree to the open peer review policy of the journal

To further support our reviewers, we have joined with Publons, where you can gain additional credit to further highlight your hard work (see: https://publons.com/journal/530/gigascience). On publication of this paper, your review will be automatically added to Publons, you can then choose whether or not to claim your Publons credit. I understand this statement.

Yes
