# Supplementary material for: Genome-scale metabolic modeling of responses to polymyxins in Pseudomonas aeruginosa
Source: Gigascience. 2018 Mar 13;7(4):giy021. doi: 10.1093/gigascience/giy021 (PMC6333913; doi:10.1093/gigascience/giy021)
Supplement: Reviewer_2_Report_(Original_Submission) -- Jonathan Monk — 12 Dec 2017 Reviewed [file giy021_reviewer_2_report_(original_submission).pdf]

Reviewer Report

Title: Genome-scale metabolic modelling of responses to polymyxins in Pseudomonas aeruginosa

Version: Original Submission **Date:** 12 Dec 2017

Reviewer name: Jonathan Monk

Reviewer Comments to Author:

We have read the study entitled "Genome-scale metabolic modelling of responses to polymyxins in Pseudomonas aeruginosa" by Zhu et al. The authors constructed a new genome-scale model for P. aeruginosa PAO1 that has 1458 genes and achieves an accuracy of 89.1% on growth sources and 87.9% for gene essentiality. The authors added several new pathways to the model compared to previous reconstructions and included a new periplasmic compartment. We commend the authors on adding the periplasmic space as well as pathways for LPS and GPL metabolism. The authors used this genome-scale model to study the effect of polymyxin treatment on this strain. Overall, we feel this is a well conducted study and that the new model will be a valuable contribution to the community. We do have a few comments and questions on the application to polymyxin treatment that need to be addressed before publication.

Major Comments

1. On page 13 line 260 in the section on lipid A modification the authors mention changes in fluxes. They state that fluxes were calculated using FBA. However, in the methods section I see that the authors used sampling to explore the solution space. The authors must use sampling to compare fluxes between conditions. If the author's used sampling here to the authors must specify so in the main text.
2. Page 14 line 286 - the authors must state how the RNAseq was used to constrain the model. They mention it in the Discussion section (E-FLUX method). However this must be stated in the results section as well.
3. Page 14 line 295 - I have a major question about how the authors simulate for growth in CAMHB media? This is an undefined media type and in the methods section they describe that they set the uptake rates to 1 mmol*gDW*hr^-1 for major carbon sources. The authors must explain why this uptake rate is justified. Did the authors perform any sensitivity analysis on these uptake rates? It's very reasonable to assume that changes in these rates would dramatically affect the fluxes described by the authors in this section. Some justification and or sensitivity analysis must be added here to explain the validity of these uptake rates for growth in this condition.
4. Page 15 line 303 - the authors must state what the "control" condition is. Is this compared to PAO1 growing in CAMHB without polymyxin treatment? Or compared to growth on a different media type, i.e. M9 minimal media + glucose?
5. The authors state that their model is "the most comprehensive for a gram-negative organism to date". On what basis is this claim made? We would recommend tempering this statement or perhaps limiting it to Pseudomonas models.

Methods

Are the methods appropriate to the aims of the study, are they well described, and are necessary controls included? Yes

Conclusions

Are the conclusions adequately supported by the data shown? Yes

Reporting Standards

Does the manuscript adhere to the journal's guidelines on minimum standards of reporting? Yes

Statistics

Are you able to assess all statistics in the manuscript, including the appropriateness of statistical tests used? Yes, and I have assessed the statistics in my report.

Quality of Written English

Please indicate the quality of language in the manuscript: Acceptable

Declaration of Competing Interests

Please complete a declaration of competing interests, considering the following questions:

- Have you in the past five years received reimbursements, fees, funding, or salary from an organisation that may in any way gain or lose financially from the publication of this manuscript, either now or in the future?
- Do you hold any stocks or shares in an organisation that may in any way gain or lose financially from the publication of this manuscript, either now or in the future?
- Do you hold or are you currently applying for any patents relating to the content of the manuscript?
- Have you received reimbursements, fees, funding, or salary from an organization that holds or has applied for patents relating to the content of the manuscript?
- Do you have any other financial competing interests?
- Do you have any non-financial competing interests in relation to this paper?

If you can answer no to all of the above, write 'I declare that I have no competing interests' below. If your reply is yes to any, please give details below.

I declare that I have no competing interests

I agree to the open peer review policy of the journal. I understand that my name will be included on my report to the authors and, if the manuscript is accepted for publication, my named report including any attachments I upload will be posted on the website along with the authors' responses. I agree for my report to be made available under an Open Access Creative Commons CC-BY license (http://creativecommons.org/licenses/by/4.0/). I understand that any comments which I do not wish to be included in my named report can be included as confidential comments to the editors, which will not be published.

I agree to the open peer review policy of the journal

To further support our reviewers, we have joined with Publons, where you can gain additional credit to further highlight your hard work (see: https://publons.com/journal/530/gigascience). On publication of this paper, your review will be automatically added to Publons, you can then choose whether or not to claim your Publons credit. I understand this statement.

Yes
